# Pd-Catalyzed, Highly Selective C(sp^2^)-Br Bond Coupling Reactions of *o*-(or *m*-, or *p*-) Chloromethyl Bromobenzene with Arylboronic Acids

**DOI:** 10.3390/molecules23020433

**Published:** 2018-02-15

**Authors:** Ming-ming Pei, Ping Liu, Yan Liu, Xin-ming Lv, Xiao-wei Ma, Bin Dai

**Affiliations:** School of Chemistry and Chemical Engineering/Key Laboratory for Green Processing of Chemical Engineering of Xinjiang Bingtuan, Shihezi University, Shihezi 832003, China; peimming@163.com (M.-m.P.); liuyan1979810@aliyun.com (Y.L.); ciqlxm@163.com (X.-m.L.); db_tea@shzu.edu.cn (B.D.)

**Keywords:** Pd-catalyzed, selective, dihalogenated hydrocarbon, arylboronic acids, coupling reaction

## Abstract

Highly selective C(sp^2^)–C(sp^2^) cross-coupling of dihalogenated hydrocarbons comprising C(sp^2^)–Br and C(sp^3^)–Cl bonds with arylboronic acids is reported. This highly selective coupling reaction of the C(sp^2^)–Br bond is successfully achieved using Pd(OAc)_2_ and PCy_3_·HBF_4_ as the palladium source and ligand, respectively. A series of chloromethyl-1,1′-biphenyl compounds are obtained in moderate-to-excellent yields. Moreover, this protocol can be extended to the one-pot dual arylation of 1-bromo-4-(chloromethyl)benzene with two arylboronic acids, leading to diverse unsymmetrical 4-benzyl-1,1′-biphenyl derivatives.

## 1. Introduction

**Scheme 1:**
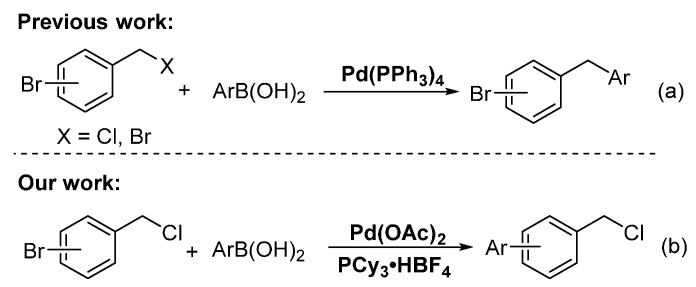
Selective palladium-catalyzed Suzuki-Miyaura coupling reaction. (a) C(sp^3^)–C(sp^2^) coupling; (b) C(sp^2^)–C(sp^2^) coupling.

Transition-metal-catalyzed cross-coupling reactions between electrophiles and arylboronic acids are an important method in the C–C bond formation [1,2,3]. Over the past decades, aryl and benzyl halides have been used as electrophiles for constructing C(sp^2^)–C(sp^2^) and C(sp^3^)–C(sp^2^) bonds, respectively [4,5,6,7,8,9,10]. To the best of our knowledge, the selective reaction of dihalogenated hydrocarbons containing the C(sp^2^)–X and C(sp^3^)–X bonds with arylboronic acids in the presence of palladium catalyst has been rarely reported [11,12,13,14,15,16]. Duchêne and Thibonnet reported the Pd(PPh_3_)_4_-catalyzed selective coupling reaction of C(sp^3^)–Br at the benzylic position of the starting bromobenzyl bromides with arylboronic acids, affording C(sp^3^)–C(sp^2^) as the coupling products (Scheme 1a) [11,12]. Gueiffier also developed a Pd(PPh_3_)_4_-catalyzed one-pot two-step reaction of bromobenzyl chloride with arylboronic acids by first C(sp^3^)–C(sp^2^) coupling and subsequent C(sp^2^)–C(sp^2^) coupling, affording numerous new unsymmetrical methylene-linked biaryl systems (Scheme 1a)[13]. Çetinkaya et al. achieved the palladium-catalyzed C(sp^2^)–C(sp^2^) coupling reactions with 1-bromo-4-(bromomethyl)benzene and arylboronic acids by using saturated *N*-heterocarbene ligands [17]. In 2010, Maseras conducted an experimental and theoretical study on the role of phosphine ligands in palladium-catalyzed Suzuki cross-coupling of competitive and selective C(sp^3^)–Br versus C(sp^2^)–Br bond activation [18]. Their results indicated that as a less-hindered phosphine, PPh_3_ is associated with a bisligated form of the catalyst, which favors the activation of the C(sp^3^)–Br bond of the α-bromosulfoxide side. As the more hindered phosphine, P(1-napthyl)_3_ is related to the monoligated form of the catalyst, which promotes the activation of the C(sp^2^)–Br bond of the bromoaryl moiety.

Inspired by this study and based on our previous studies [19,20,21,22,23,24,25], we reported Pd-catalyzed, highly selective C(sp^2^)-Br bond coupling reactions of *o*-(or *m*-, or *p*-)chloromethyl bromobenzene with arylboronic acids in the presence of the ligand PCy_3_·HBF_4_, which does not afford C(sp^3^)–C(sp^2^) coupling products; instead, the reaction provided highly selective of C(sp^2^)–C(sp^2^) coupling products (Scheme 1b).

## 2. Results and Discussion

Initially, 1-bromo-4-(chloromethyl)benzene and *p*-tolylboronic acid were selected as model substrates to optimize the reaction conditions. Table 1 summarizes the results obtained. The screened bases were examined by using PCy_3_·HBF_4_ and Pd(OAc)_2_ as the ligand and palladium source, respectively, in toluene/water (1/0.1) at 80 °C for 2 h; Cs_2_CO_3_ was the most effective base, affording the desired product in 99% yield (entry 5). On the other hand, other bases such as K_2_CO_3_, K_3_PO_4_·3H_2_O, NaOH, and NEt_3_ afforded the desired products in 16–84% yields (entries 1–4). Remarkably, the ligand was found to play an important role in this reaction, and PPh_3_ was not effective for this selective C(sp^2^)–C(sp^2^) coupling reaction (entry 6). Moreover, with the decrease in the reaction temperature to 60 °C, the product was obtained in only 74% yield (entry 7). With the decrease in the catalyst amount from 1 mol % to 0.2 mol %, the desired product was still obtained in a gas chromatography-mass spectrometer (GC–MS) yield of 99% (entries 8–10). However, with the decrease in the catalyst loading to 0.1 mol %, the yield was significantly reduced (entry 11). Finally, the combination of Pd(OAc)_2_ (0.2 mol %)/PCy_3_·HBF_4_ (0.4 mol %) and Cs_2_CO_3_ (2 equiv.) at 80 °C for 2 h in toluene/water (1 mL/0.1 mL) was found to constitute the optimum reaction conditions.

With the optimized reaction conditions in hand, the substrate scope of this selective Suzuki–Miyaura reaction was examined. First, the coupling reactions of 1-bromo-4-(chloromethyl)benzene with arylboronic acid were explored under the optimized reaction conditions. 4-Substituted arylboronic acids bearing electron-donating or electron-withdrawing groups selectively underwent the coupling reaction, affording corresponding products **3b**–**3g** in 75–93% yields (Table 2). Furthermore, the selective coupling reaction of 1-bromo-4-(chloromethyl)benzene, with *m*-tolylboronic acid, and (3-chlorophenyl)boronic acid afforded the desired products **3h** and **3i** in 98% and 73% yields, respectively. Sterically demanding ortho substituents, such as *o*-tolylboronic acid, did not impair the coupling reaction, affording the desired product **3j** in 90% yield. However, (2,3-difluorophenyl)boronic acid and (2,6-dimethylphenyl)boronic acid as substrates afforded coupling products **3k** and **3l**, respectively, in low yields. In addition, thiophen-3-ylboronic acid, naphthalen-2-ylboronic acid, and 4-vinylphenylboronic acid were tolerated, affording desired products **3n**–**3p** in 57–86% yields.

Next, the selective coupling reactions of 1-bromo-3-(chloromethyl)benzene with various arylboronic acids were investigated (Table 3). The results indicated that neither the electronic property nor the steric hindrance of the substrates clearly affects the coupling reaction: The desired products **4a**–**4h** were obtained in 73–95% yields.

To further ascertain the application scope of the catalytic system, the reaction of 1-bromo-2-(chloromethyl)benzene with arylboronic acids was examined. The present catalytic method can be applied for the selective coupling of 1-bromo-2-(chloromethyl)benzene with arylboronic acids, affording the desired products **5a**–**5h** in yields of 80–95% (Table 4).

A one-pot dual Suzuki coupling reaction for successively substituting 4-bromobenzyl chloride with distinct aryl groups was contemplated, which could provide a straightforward route for obtaining diverse 4-benzyl-1,1′-biphenyl derivatives (Table 5). First, 4-bromobenzyl chloride was treated with 1.1 equivalent of *p*-tolylboronic acid in the presence of 2 mol % of Pd(OAc)_2_, 0.4 mol % of PCy_3_·HBF_4_, and 5.0 equiv. of Cs_2_CO_3_ in a mixture of toluene and water (10:1). After heating for 2 h at 80 °C, 1.0 equivalent of arylboronic acid and 4.0 mol % of PPh_3_ were added to the reaction system. The reaction mixture was stirred for 5 h at 80 °C, affording the desired products **6a**–**6d** in 57–96% yields.

**Scheme 2:**
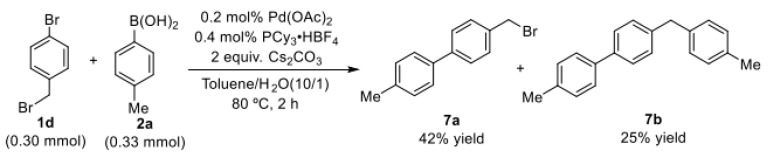
Selective coupling reaction of 1-bromo-4-(bromo methyl)benzene with *p*-tolylboronic acid.

Next, the coupling reactions of 1-bromo-4-(bromomethyl)benzene (**1d**) with p-tolylboronic acid (**2a**) were investigated under the optimized conditions. We found that the selective reaction of C(sp^2^)–Br bond with arylboronic acids could proceed smoothly to give 42% of the desired product **7a**, also accompanied by 25% yield of bis-coupling product **7b** (Scheme 2). This experimental result shows that the selectivity of the reaction depends not only on the regulatory effect of the phosphine ligand but also on the structure of the C(sp^3^)–X bond.

The selectivity depended exclusively on the palladium ligands.

**Scheme 3:**
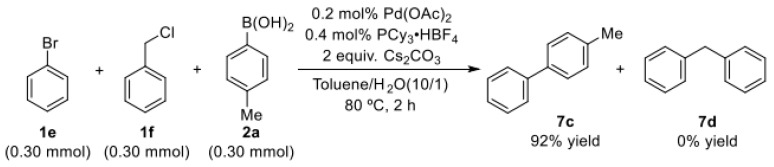
Selective coupling reaction of bromobenzene or (chloromethyl)benzene with *p*-tolylboronic acid.

To demonstrate the importance of phosphine ligand for palladium-catalyzed selective coupling reactions of C(sp^2^)–Br bond or C(sp^3^)–Cl bond with arylboronic acid, the external competition experiment was performed. To this end, we designed an experiment in which mixtures of bromobenzene and (chloromethyl)benzene were allowed to react with *p*-tolylboronic acid (Scheme 3). As expected, the formation of the Csp^2^–Csp^2^ cross-coupling product (**7c**) was achieved in the competitive experiment when PCy_3_∙HBF_4_ was used as the phosphine ligand in the palladium catalyst.

## 3. Materials and Methods

Chemicals were obtained commercially and used as received. Nuclear magnetic resonance (NMR) spectra were recorded on a Bruker DPX–400 spectrometer (Bruker Co., Billerica, MA, USA) using tetramethylsilane (TMS) as the internal standard. Electric impact ionization (EI)–Mass spectrum was measured on a gas chromatography time of flight high resolution mass spectrometry (GCTOF-HRMS) (Waters Co, Milford, MA, USA). or GC-MS (Agilent 7890A/5975C, Santa Clara, CA, USA) instrument. Electrospray ionization (ESI)–Mass spectrum was measured on a matrix-assisted laser desorption/ionization time of flight mass spectrometry (MALDI-TOF MS) (Bruker Co., Bremen, Germany). To all copies of 1H NMR, ^13^C NMR and HRMS spectra, please see Figures S2–S45 in Supplementary Materials. All products were isolated by short chromatography on a silica gel (200–300 mesh) column using petroleum ether (60–90 °C), unless otherwise noted. Arylboronic acids and o-(or m-, or p-)chloromethyl bromobenzene were of analytical grade quality, purchased from Adamas-beta Pharmaceuticals, Inc. (Shanghai, China).

### 3.1. General Procedure for the Selective Coupling Reaction of o-(or m-, or p-)chloromethyl Bromobenzene with Arylboronic Acid

A Schlenk tube (20 mL) was charged with *o*-(or *m*-, or *p*-)chloromethyl bromobenzene (0.3 mmol), arylboronic acid (0.33 mmol), Pd(OAc)_2_ (0.2 mol %), PCy_3_·HBF_4_ (0.4 mol %), and Cs_2_CO_3_ (2 equiv.). The tube was degassed for 30 s and then was filled with argon. This operation was repeated three times. After toluene (1.0 mL) and H_2_O (0.1 mL) were added under argon atmosphere, the resulting reaction mixture was stirred at 80 °C for 2 h under argon. After the completion of the reaction, the reaction mixture was allowed to cool to room temperature. The solution was quenched with water (10 mL) and extracted with EtOAc (3 × 10 mL). The combined EtOAc extracts were dried over anhydrous Na_2_SO_4_ and filtered, followed by solvent removal under reduced pressure. The residue was purified by flash column chromatography on silica gel using petroleum ether/EtOAc as the eluent.

### 3.2. General Procedure for One-Pot Dual Arylations of 1-Bromo-4-(chloromethyl)benzene

A Schlenk tube (20 mL) was charged with 1-bromo-4-(chloromethyl)benzene (0.3 mmol), arylboronic acid (0.33 mmol), 2 mol % Pd(OAc)_2_, 0.4 mol % PCy_3_·HBF_4_, and 5 equiv. Cs_2_CO_3_. The tube was degassed for 30 s and then was filled with argon. This operation was repeated for three times. After toluene (1.0 mL) and H_2_O (0.1 mL) were added under argon atmosphere, the resulting reaction mixture was stirred at 80 °C for 2 h under argon. After the completion of the reaction, the solution was allowed to cool to room temperature. Then, another arylboronic acid (0.33 mmol) and 4 mol % PPh_3_ were introduced under argon. The reaction mixture was heated at 80 °C for 5 h. The solution was quenched with water (10 mL) and extracted with EtOAc (3 × 10 mL). The combined EtOAc extracts were dried over anhydrous Na_2_SO_4_, filtrated, and then the solvent was removed under reduced pressure. The residue was purified by flash column chromatography on silica gel with PE/EtOAc as the eluent.

*4-*(*chloromethyl*)*-4′-methyl-1,1′-biphenyl* (**3a**) [26]: Colorless oil (64.3 mg, 99%). ^1^H-NMR (400 MHz, CDCl_3_) δ 7.57 (d, *J* = 8.4 Hz, 2H), 7.48 (d, *J* = 8.4 Hz, 2H), 7.44 (d, *J* = 8.0 Hz, 2H), 7.25 (d, *J* = 8.0 Hz, 2H), 4.63 (s, 2H), 2.40 (s, 3H). ^13^C-NMR (100 MHz, CDCl_3_) δ 141.48, 137.75, 137.53, 136.28, 129.69, 129.18, 127.43, 127.10, 46.27, 21.27.

*4-*(*chloromethyl*)*-1,1′-biphenyl* (**3b**) [27]: Yellow oil (47.9 mg, 79%). ^1^H-NMR (400 MHz, CDCl_3_) *δ* 7.58 (d, *J* = 7.2 Hz, 4H), 7.51–7.40 (m, 4H), 7.39–7.32 (m, 1H), 4.63 (s, 2H). ^13^C-NMR (100 MHz, CDCl_3_) δ 141.53, 140.63, 136.58, 129.19, 128.96, 127.67, 127.63, 127.26, 46.19.

*4-*(*chloromethyl*)*-4′-methoxy-1,1′-biphenyl* (**3c**) [28]: Colorless oil (60.7 mg, 87%). ^1^H-NMR (400 MHz, CDCl_3_) δ 7.54 (d, *J* = 8.8 Hz, 2H), 7.52 (d, *J* = 8.8 Hz, 2H), 7.44 (d, *J* = 8.4 Hz, 2H), 6.98 (d, *J* = 8.8 Hz, 2H), 4.63 (s, 2H), 3.85 (s, 3H). ^13^C-NMR (100 MHz, CDCl_3_) δ 159.50, 141.16, 135.94, 133.15, 129.19, 128.29, 127.17, 114.41, 55.51, 46.30.

*4-*(*chloromethyl*)*-4′-propyl-1,1′-biphenyl* (**3d**): Colorless oil (60.9 mg, 83%). ^1^H-NMR (400 MHz, CDCl_3_) δ 7.57 (d, *J* = 8.4 Hz, 2H), 7.50 (d, *J* = 8.4 Hz, 2H), 7.44 (d, *J* = 8.4 Hz, 2H), 7.26 (s, 1H), 7.24 (d, *J* = 2.8 Hz, 1H), 4.63 (s, 2H), 2.65–2.60 (m, 2H), 1.68 (dq, *J* = 14.8, 7.2 Hz, 2H), 0.97 (t, *J* = 7.3 Hz, 3H). ^13^C-NMR (100 MHz, CDCl_3_) δ 142.33, 141.51, 137.97, 136.24, 129.16, 129.09, 127.44, 127.07, 46.27, 37.84, 24.69, 14.03. HRMS (ESI) *m*/*z* calcd for C_16_H_17_ClNa^+^ (M + Na)^+^ 267.09110, found 267.09195.

*4-*(*chloromethyl*)*-4′-pentyl-1,1′-bipheny* (**3e**) [29]: White solid (75.8 mg, 93%). ^1^H-NMR (400 MHz, CDCl_3_) δ 7.57 (d, *J* = 8.4 Hz, 2H), 7.50 (d, *J* = 8.0 Hz, 2H), 7.44 (d, *J* = 8.4 Hz, 2H), 7.26 (s, 1H), 7.25 (d, *J* = 3.2 Hz, 1H), 4.63 (s, 2H), 2.67–2.61 (m, 2H), 1.65 (p, *J* = 7.4 Hz, 2H), 1.39–1.32 (m, 4H), 0.93–0.88 (m, 3H). ^13^C-NMR (100 MHz, CDCl_3_) δ 142.60, 141.51, 137.93, 136.24, 129.16, 129.03, 127.44, 127.09, 46.28, 35.73, 31.70, 31.31, 22.71, 14.19.

*4-*(*chloromethyl*)*-4′-fluoro-1,1′-biphenyl* (**3f**) [30]: Colorless oil (54.9 mg, 83%). ^1^H-NMR (400 MHz, CDCl_3_) δ 7.55 (d, *J* = 3.2 Hz, 1H), 7.54–7.51 (m, 3H), 7.45 (d, *J* = 8.0 Hz, 2H), 7.12 (t, *J* = 8.8 Hz, 2H), 4.63 (s, 2H). ^13^C-NMR (100 MHz, CDCl_3_) δ 162.74, 140.53, 136.63, 129.41, 129.25, 128.82, 127.48, 115.85, 46.10.

*4-*(*chloromethyl*)*-4′-*(*trifluoromethyl*)*-1,1′-biphenyl* (**3g**) [31]: Colorless oil (60.9 mg, 75%). ^1^H-NMR (400 MHz, CDCl_3_) δ 7.69 (d, *J* = 1.6 Hz, 4H), 7.59 (d, *J* = 8.4 Hz, 2H), 7.50 (d, *J* = 8.0 Hz, 2H), 4.64 (s, 2H). ^13^C-NMR (100 MHz, CDCl_3_) δ 144.14, 140.03, 137.63, 129.95–129.57 (m), 129.39, 127.79, 127.55, 125.93 (q, *J* = 4.0 Hz),123.78 (dd, *J* =420.0, 271.0 Hz) 45.94.

*4′-*(*chloromethyl*)*-3-methyl-1,1′-biphenyl* (**3h**): Colorless oil (63.7 mg, 98%). ^1^H-NMR (400 MHz, CDCl_3_) δ 7.56 (d, *J* = 8.4 Hz, 2H), 7.42 (d, *J* = 8.4 Hz, 2H), 7.37 (d, *J* = 8.8 Hz, 2H), 7.31 (t, *J* = 7.6 Hz, 1H), 7.16 (d, *J* = 7.6 Hz, 1H), 4.61 (s, 2H), 2.40 (s, 3H). ^13^C-NMR (100 MHz, CDCl_3_) δ 141.61, 140.59, 138.52, 136.45, 129.11, 128.85, 128.40, 128.02, 127.59, 124.35, 46.20, 21.66. HRMS (EI): *m*/*z* calcd for C_14_H_13_Cl [M]: 216.0723, found [M]: 216.0723.

*3-chloro-4′-*(*chloromethyl*)*-1,1′-biphenyl* (**3i**): Colorless oil (51.9 mg, 73%). ^1^H-NMR (400 MHz, CDCl_3_) δ 7.54 (d, *J* = 8.4 Hz, 3H), 7.47–7.42 (m, 3H), 7.38–7.30 (m, 2H), 4.62 (s, 2H). ^13^C-NMR (100 MHz, CDCl_3_) δ 142.42, 140.02, 137.24, 134.84, 130.18, 129.28, 127.66, 127.56, 127.35, 125.37, 45.99. HRMS (EI): *m*/*z* calcd for C_13_H_10_Cl_2_ [M]: 236.0169, found [M]: 236.0160.

*4′-*(*chloromethyl*)*-2-methyl-1,1′-biphenyl* (**3j**) [32]: Colorless oil (58.5 mg, 90%). ^1^H-NMR (400 MHz, CDCl_3_) δ 7.43 (d, *J* = 8.0 Hz, 2H), 7.32 (d, *J* = 8.4 Hz, 2H), 7.28–7.25 (m, 2H), 7.25–7.19 (m, 2H), 4.64 (s, 2H), 2.27 (s, 3H). ^13^C-NMR (100 MHz, CDCl_3_) δ 142.29, 141.35, 136.06, 135.44, 130.52, 129.86, 129.71, 128.49, 127.60, 125.97, 46.28, 20.59.

*4′-*(*chloromethyl*)*-2,3-difluoro-1,1′-biphenyl* (**3k**): Yellow oil (33.6 mg, 47%). ^1^H-NMR (400 MHz, CDCl_3_) δ 7.54 (d, *J* = 6.8 Hz, 2H), 7.48 (d, *J* = 8.4 Hz, 2H), 7.21–7.08 (m, 3H), 4.63 (s, 2H). ^13^C-NMR (100 MHz, CDCl_3_) δ 151.25 (dd, *J* = 247.0, 14.0 Hz), 148.12 (dd, *J* = 249.0, 14.0 Hz), 137.55, 134.96 (d, *J* = 4.0 Hz), 130.75 (d, *J* = 10.0 Hz), 129.45 (d, *J* = 3.0 Hz), 128.95, 125.46–125.27 (m), 124.29 (dd, *J* = 7.0, 5.0 Hz), 116.46 (d, *J* = 20.0 Hz), 45.96. HRMS (EI): *m*/*z* calcd for C_13_H_9_ClF_2_ [M]: 238.0369, found [M]: 238.0361.

*4′-*(*chloromethyl*)*-2,6-dimethyl-1,1′-biphenyl* (**3l**): Colorless oil (34.6 mg, 50%). ^1^H-NMR (400 MHz, CDCl_3_) δ 7.44 (d, *J* = 8.0 Hz, 2H), 7.15 (t, *J* = 2.9 Hz, 2H), 7.13 (s, 1H), 7.10 (d, *J* = 7.6 Hz, 2H), 4.65 (s, 2H), 2.02 (s, 6H). ^13^C-NMR (100 MHz, CDCl_3_) δ 140.29, 139.54, 137.04, 132.09, 129.33, 128.84, 127.43, 122.00, 46.05, 29.85. HRMS (ESI) *m*/*z* calcd for C_15_H_16_Cl^+^ (M + H)^+^ 231.09350, found 231.09262.

*4′-*(*chloromethyl*)*-3,4,5-trifluoro-1,1′-biphenyl* (**3m**) [33]: Colorless oil (56.2 mg, 73%). ^1^H-NMR (400 MHz, CDCl_3_) δ 7.48 (d, *J* = 1.6 Hz, 4H), 7.17 (dd, *J* = 6.4, 6.4 Hz, 2H), 4.62 (s, 2H). ^13^C-NMR (100 MHz, CDCl_3_) δ 151.60 (ddd, *J* = 248.0, 10.0, 4.0 Hz), 141.05–138.04 (m), 138.43, 137.88, 136.74 (td, *J* = 7.7, 4.6 Hz), 129.46, 127.3, 111.34–111.04 (m), 45.77.

*3-*(*4-*(*chloromethyl*)*phenyl*)*thiophene* (**3n**) [34]: Colorless oil (48.2 mg, 77%). ^1^H-NMR (400 MHz, CDCl_3_) δ 7.58 (d, *J* = 8.4 Hz, 2H), 7.45 (dd, *J* = 1.6, 1.6 Hz, 1H), 7.40 (d, *J* = 8.4 Hz, 2H), 7.38 (d, *J* = 1.2 Hz, 1H), 7.37 (s, 1H), 4.60 (s, 2H). ^13^C-NMR (100 MHz, CDCl_3_) δ 141.78, 136.3, 136.13, 129.26, 126.88, 126.52, 126.38, 120.84, 46.21.

*2-*(*4-*(*chloromethyl*)*phenyl*)*naphthalene* (**3o**) [35]: Colorless oil (65.2 mg, 86%). ^1^H-NMR (400 MHz, CDCl_3_) δ 8.00 (s, 1H), 7.90–7.82 (m, 3H), 7.71–7.66 (m, 3H), 7.51–7.44 (m, 4H), 4.62 (s, 2H). ^13^C-NMR (100 MHz, CDCl_3_) δ 141.43, 137.93, 136.68, 133.76, 132.86, 129.30, 128.66, 128.36, 127.89, 127.79, 126.53, 126.24, 126.02, 125.53, 46.21.

*4-*(*chloromethyl*)*-4′-vinyl-1,1′-biphenyl* (**3p**): Colorless oil (39.1 mg, 57%). ^1^H-NMR (400 MHz, CDCl_3_) δ 7.59 (d, *J* = 8.0 Hz, 2H), 7.56 (d, *J* = 8.0 Hz, 2H), 7.49 (s, 1H), 7.47 (d, *J* = 2.8 Hz, 2H), 7.45 (s, 1H), 6.76 (dd, *J* = 10.8, 10.8 Hz, 1H), 5.80 (d, *J* = 17.6 Hz, 1H), 5.28 (d, *J* = 10.8 Hz, 1H), 4.64 (s, 2H). ^13^C-NMR (100 MHz, CDCl_3_) δ 140.98, 139.91, 137.00, 136.62, 136.44, 129.21, 127.39, 127.31, 126.83, 114.24, 46.17. HRMS (ESI) *m*/*z* calcd for C_15_H_13_ClNa^+^ (M + Na)^+^ 251.05980, found 251.06100.

*3-*(*chloromethyl*)*-1,1′-biphenyl* (**4a**) [36]: Yellow solid (57.8 mg, 95%). ^1^H-NMR (400 MHz, CDCl_3_) δ 7.62–7.59 (m, 2H), 7.58 (d, *J* = 0.8 Hz, 1H), 7.55 (dd, *J* = 7.6, 1.2 Hz, 1H), 7.47–7.41 (m, 3H), 7.38 (s, 1H), 7.36 (d, *J* = 1.6 Hz, 1H), 4.65 (s, 2H). ^13^C-NMR (100 MHz, CDCl_3_) δ 141.96, 140.73, 138.10, 129.32, 128.96, 127.69, 127.56, 127.54, 127.36, 127.31, 46.41.

*3-*(*chloromethyl*)*-4′-methyl-1,1′-biphenyl* (**4b**) [36]: Colorless oil (58.5 mg, 90%). ^1^H-NMR (400 MHz, CDCl_3_) δ 7.58 (s, 1H), 7.51 (d, *J* = 7.6 Hz, 1H), 7.47 (d, *J* = 8.0 Hz, 2H), 7.40 (t, *J* = 7.6 Hz, 1H), 7.33 (d, *J* = 7.6 Hz, 1H), 7.24 (d, *J* = 7.6 Hz, 2H), 4.62 (s, 2H), 2.38 (s, 3H). ^13^C-NMR (100 MHz, CDCl_3_) δ 141.87, 138.04, 137.83, 137.50, 129.67, 129.27, 127.35, 127.26, 127.16, 127.13, 46.46, 21.25.

*3-*(*chloromethyl*)*-4′-*(*trifluoromethyl*)*-1,1′-biphenyl* (**4c**) [37]: Colorless oil (74.7 mg, 92%). ^1^H-NMR (400 MHz, CDCl_3_) δ 7.70–7.64 (m, 4H), 7.59 (s, 1H), 7.52 (dt, *J* = 7.4, 1.6 Hz, 1H), 7.44 (t, *J* = 7.6 Hz, 1H), 7.41 (d, *J* = 7.6 Hz, 1H), 4.63 (s, 2H). ^13^C-NMR (100 MHz, CDCl_3_) δ 144.23, 140.49, 138.44, 129.78 (d, *J* = 32.0 Hz), 129.57, 128.45, 127.63, 127.60, 127.46, 125.91 (q, *J* = 4.0 Hz), 124.38 (q, *J* = 270.0 Hz), 46.15.

*3-*(*chloromethyl*)*-3′-methyl-1,1′-biphenyl* (**4d**): Colorless oil (60.4 mg, 93%). ^1^H-NMR (400 MHz, CDCl_3_) δ 7.59 (s, 1H), 7.52 (d, *J* = 7.6 Hz, *1*H), 7.40 (q, *J* = 7.6 Hz, 3H), 7.33 (dd, *J* = 13.6, 5.6 Hz, 2H), 7.17 (d, *J* = 7.2 Hz, 1H), 4.63 (s, 2H), 2.41 (s, 3H). ^13^C-NMR (100 MHz, CDCl_3_) δ 142.06, 140.70, 138.55, 138.02, 129.24, 128.85, 128.42, 128.08, 127.55, 127.44, 127.36, 124.40, 46.43, 1.66. GC-MS (*m*/*z*): 217.

*3-chloro-3′-*(*chloromethyl*)*-1,1′-biphenyl* (**4e**): Colorless oil (65.4 mg, 92%). ^1^H-NMR (400 MHz, CDCl_3_) δ 7.55 (d, *J* = 4.0 Hz, 2H), 7.49 (d, *J* = 7.2 Hz, 1H), 7.44 (d, *J* = 7.2 Hz, 1H), 7.41 (d, *J* = 7.6 Hz, 1H), 7.39–7.34 (m, 2H), 7.33–7.31 (m, 1H), 4.62 (s, 2H). ^13^C-NMR (100 MHz, CDCl_3_) δ 142.56, 140.55, 138.32, 134.87, 130.20, 129.48, 128.14, 127.71, 127.50, 127.45, 127.31, 125.47, 46.23. HRMS (EI): *m*/*z* calcd for C_13_H_10_Cl_2_ [M]: 236.0172, found [M]: 236.0160.

*3′-*(*chloromethyl*)*-2-methyl-1,1′-biphenyl* (**4f**) [32]: Colorless oil (56.6 mg, 87%). ^1^H-NMR (400 MHz, CDCl_3_) δ 7.36 (dd, *J* = 12.4, 7.2 Hz, 3H), 7.27 (d, *J* = 9.6 Hz, 3H), 7.22 (t, *J* = 5.4 Hz, 2H), 4.61 (s, 2H), 2.26 (s, 3H). ^13^C-NMR (100 MHz, CDCl_3_) δ 142.59, 141.37, 137.43, 135.41, 130.50, 129.84, 129.54, 129.36, 128.60, 127.61, 127.07, 125.95, 46.38, 20.56.

*3-*(*3-*(*chloromethyl*)*phenyl*)*thiophene* (**4g**) [32]: Colorless oil (59.5 mg, 95%). ^1^H-NMR (400 MHz, CDCl_3_) δ 7.61 (s, 1H), 7.55 (dt, *J* = 7.6, 1.6 Hz, 1H), 7.47 (dd, *J* = 2.4, 2.0 Hz, 2H), 7.43–7.34 (m, 4H), 7.31 (d, *J* = 8.0 Hz, 1H), 4.62 (s, 2H). ^13^C-NMR (100 MHz, CDCl_3_) δ 141.83, 138.13, 136.52, 129.35, 127.38, 126.79, 126.61, 126.52, 126.39, 120.86, 46.35. 

*2-*(*3-*(*chloromethyl*)*phenyl*)*naphthalene* (**4h**): Yellow oil (55.3 mg, 73%). ^1^H-NMR (400 MHz, CDCl_3_) δ 8.02 (s, 1H), 7.89 (t, *J* = 7.2 Hz, 2H), 7.85 (d, *J* = 7.2 Hz, 1H), 7.71 (d, *J* = 6.4 Hz, 2H), 7.65 (d, *J* = 7.6 Hz, 1H), 7.52–7.46 (m, 2H), 7.44 (d, *J* = 7.6 Hz, 1H), 7.38 (d, *J* = 7.6 Hz, 1H), 4.66 (s, 2H). ^13^C-NMR (100 MHz, CDCl_3_) δ 141.86, 138.22, 138.03, 133.76, 132.86, 129.43, 128.66, 128.35, 127.79, 127.64, 126.53, 126.24, 126.07, 125.58, 46.44. HRMS (EI): *m*/*z* calcd for C_17_H_10_Cl [M]: 252.0723, found [M]: 252.0706.

*2-*(*chloromethyl*)*-1,1′-biphenyl* (**5a**) [38]: Yellow oil (58.9 mg, 97%). ^1^H-NMR (400 MHz, CDCl_3_) δ 7.55–7.51 (m, 1H), 7.44 (d, *J* = 8.8 Hz, 1H), 7.42–7.33 (m, 6H), 7.27 (d, *J* = 9.2 Hz, 1H), 4.52 (s, 2H). ^13^C-NMR (100 MHz, CDCl_3_) δ 142.21, 140.31, 135.05, 130.64, 130.46, 129.28, 128.64, 128.44, 128.07, 127.59, 44.60.

*2-*(*chloromethyl*)*-4′-methyl-1,1′-biphenyl* (**5b**) [39]: Yellow oil (61.7 mg, 95%). ^1^H-NMR (400 MHz, CDCl_3_) δ 7.55–7.50 (m, 1H), 7.38–7.33 (m, 2H), 7.31 (d, *J* = 8.2 Hz, 2H), 7.28–7.22 (m, 3H), 4.53 (s, 2H), 2.41 (s, 3H). ^13^C-NMR (100 MHz, CDCl_3_) δ 142.19, 137.38, 137.29, 135.08, 130.61, 130.50, 129.15, 129.13, 128.61, 127.86, 44.68, 21.33.

*2-*(*chloromethyl*)*-4′-*(*trifluoromethyl*)*-1,1′-biphenyl* (**5c**) [39]: Colorless oil (72.3 mg, 89%). ^1^H-NMR (400 MHz, CDCl_3_) δ 7.71 (d, *J* = 8.4 Hz, 2H), 7.56 (d, *J* = 7.6 Hz, 3H), 7.46–7.38 (m, 2H), 7.27 (d, *J* = 10.0 Hz, 1H), 4.48 (s, 2H). ^13^C-NMR (100 MHz, CDCl_3_) δ 143.98, 140.80, 135.04, 130.92, 130.28,129.89 (dt, *J* = 95.0, 30 Hz), 129.68, 128.91, 128.79, 125.43 (q, *J* = 4.0 Hz), 124.34 (q, *J* = 270.0 Hz), 44.25.

*2-*(*chloromethyl*)*-3′-methyl-1,1′-biphenyl* (**5d**) [39]: Yellow oil (59.8 mg, 92%). ^1^H-NMR (400 MHz, CDCl_3_) δ 7.56–7.51 (m, 1H), 7.38–7.34 (m, 2H), 7.31 (d, *J* = 7.2 Hz, 1H), 7.29–7.25 (m, 1H), 7.21 (d, *J* = 6.8 Hz, 5H), 4.53 (s, 2H), 2.41 (s, 3H). ^13^C-NMR (100 MHz, CDCl_3_) δ 142.32, 140.25, 138.06, 135.03, 130.58, 130.40, 130.03, 128.56, 128.31, 128.29, 127.95, 126.33, 44.64, 21.62.

*3′-chloro-2-*(*chloromethyl*)*-1,1′-biphenyl* (**5e**) [39]: Yellow oil (62.3 mg, 88%). ^1^H-NMR (400 MHz, CDCl_3_) δ 7.53 (dd, *J* = 7.6, 2.0 Hz, 1H), 7.40 (d, *J* = 1.2 Hz, 1H), 7.38 (dd, *J* = 4.8, 1.6 Hz, 1H), 7.37–7.32 (m, 3H), 7.32–7.29 (m, 1H), 7.25–7.22 (m, 1H), 4.49 (s, 2H). ^13^C-NMR (100 MHz, CDCl_3_) δ 142.04, 140.74, 135.02, 134.33, 130.76, 130.29, 129.67, 129.37, 128.78, 128.55, 127.79, 127.49, 44.31.

*2-*(*chloromethyl*)*-2′-methyl-1,1′-biphenyl* (**5f**) [40]: Colorless oil (56.5 mg, 87%). ^1^H-NMR (400 MHz, CDCl_3_) δ 7.55 (d, *J* = 6.8 Hz, 1H), 7.42–7.30 (m, 3H), 7.30–7.20 (m, 3H), 7.20–7.11 (m, 2H), 4.43–4.26 (m, 2H), 2.07 (s, 3H). ^13^C-NMR (100 MHz, CDCl_3_) δ 141.57, 139.60, 136.16, 135.41, 130.17, 130.09, 129.69, 128.41, 128.01, 127.92, 125.64, 44.26, 20.30.

*3-*(*2-*(*chloromethyl*)*phenyl*)*thiophene* (**5g**) [41]: Colorless oil (59.4 mg, 95%). ^1^H-NMR (400 MHz, CDCl_3_) δ 7.51 (dd, *J* = 7.2 3.6 Hz, 1H), 7.42 (dd, *J* = 2.8, 1.2 Hz, 1H), 7.39 (dd, *J* = 5.2, 3.2 Hz, 1H), 7.35 (d, *J* = 3.2 Hz, 3H), 7.26–7.22 (m, 1H), 4.58 (s, 2H). ^13^C-NMR (100 MHz, CDCl_3_) δ 140.44, 136.90, 135.18, 130.92, 130.41, 128.98, 128.80, 128.08, 125.76, 123.44, 44.95.

*2-*(*2-*(*chloromethyl*)*phenyl*)*naphthalene* (**5h**) [42]: Yellow oil (60.6 mg, 80%). ^1^H-NMR (400 MHz, CDCl_3_) δ 7.90–7.83 (m, 4H), 7.57–7.51 (m, 2H), 7.51–7.46 (m, 2H), 7.38 (dd, *J* = 2.8, 2.0 Hz, 1H), 7.37–7.33 (m, 2H), 4.54 (s, 2H). ^13^C-NMR (100 MHz, CDCl_3_) δ 142.13, 137.76, 135.26, 133.28, 130.72, 130.66, 128.68, 128.29, 128.16, 128.04, 127.84, 127.49, 126.54

*4-benzyl-4′-methyl-1,1′-biphenyl* (**6a**) [43]: Yellow oil (60.4 mg, 78%). ^1^H-NMR (400 MHz, CDCl_3_) δ 7.51 (d, *J* = 2.0 Hz, 1H), 7.48 (dd, *J* = 3.6, 2.0 Hz, 2H), 7.45 (d, *J* = 2.0 Hz, 1H), 7.30 (t, *J* = 6.4 Hz, 2H), 7.26–7.20 (m, 7H), 4.01 (s, 2H), 2.38 (s, 3H). ^13^C-NMR (100 MHz, CDCl_3_) δ 141.20, 140.07, 139.11, 138.26, 136.96, 129.59, 129.42, 129.11, 128.65, 127.16, 126.98, 126.26, 41.73, 21.24.

*4-*(*4-methoxybenzyl*)*-4′-methyl-1,1′-biphenyl* (**6b**) [44]: Yellow oil (83.1 mg, 96%). ^1^H-NMR (400 MHz, CDCl_3_) δ 7.49 (s, 1H), 7.47 (d, *J* = 2.0 Hz, 2H), 7.45 (s, 1H), 7.23 (s, 2H), 7.21 (s, 2H), 7.14 (s, 1H), 7.12 (s, 1H), 6.85 (s, 1H), 6.83 (s, 1H), 3.95 (s, 2H), 3.77 (s, 3H), 2.37 (s, 3H). ^13^C-NMR (100 MHz, CDCl_3_) δ 158.14, 140.54, 139.02, 138.29, 136.93, 133.32, 130.03, 129.57, 129.28, 127.13, 126.97, 114.06, 55.41, 40.82, 21.23.

*4-*(*4-fluorobenzyl*)*-4′-methyl-1,1′-biphenyl* (**6c**): Yellow oil (73.7 mg, 89%).^1^H-NMR (400 MHz, CDCl_3_) δ 7.50 (d, *J* = 8.0 Hz, 2H), 7.46 (d, *J* = 8.0 Hz, 2H), 7.24–7.20 (m, 4H), 7.17 (dd, *J* = 8.8, 5.6 Hz, 2H), 6.98 (t, *J* = 8.8 Hz, 2H), 3.97 (s, 2H), 2.38 (s, 3H). ^13^C-NMR (100 MHz, CDCl_3_) δ 161.59 (d, *J* = 243.9 Hz), 139.86 (d, *J* = 1.4 Hz), 139.26, 138.16, 137.04, 136.85 (d, *J* = 3.3 Hz), 130.45 (d, *J* = 7.8 Hz), 129.60, 129.31, 127.22, 126.98, 115.40 (d, *J* = 21.2 Hz), 40.86, 21.24. HRMS (ESI) *m*/*z* calcd for C_20_H_19_FK^+^ (M + K)^+^ 315.09459, found 315.09357.

*3-((4′-methyl-[1,1′-biphenyl]-4-yl)methyl)thiophene* (**6d**): Colorless oil (45.2 mg, 57%). ^1^H-NMR (400 MHz, CDCl_3_) δ 7.50 (d, *J* = 8.4 Hz, 2H), 7.47 (d, *J* = 8.0 Hz, 2H), 7.26 (t, *J* = 2.4 Hz, 2H), 7.23 (d, *J* = 8.0 Hz, 3H), 6.95 (dd, *J* = 4.8, 2.8 Hz, 2H), 4.01 (s, 2H), 2.38 (s, 3H). ^13^C-NMR (100 MHz, CDCl_3_) δ 139.53, 139.20, 136.99, 129.59, 129.24, 128.61, 127.17, 126.98, 125.81, 121.44, 36.30, 21.24. HRMS (ESI) *m*/*z* calcd for C_18_H_17_S^+^ (M + H)^+^ 265.10455, found 265.10495.

*4-*(*bromomethyl*)*-4′-methyl-1,1′-biphenyl* (**7a**) [45]: White solid (32.6 mg, 42%). ^1^H-NMR (400 MHz, CDCl_3_) δ 7.55 (d, *J* = 8.4 Hz, 2H), 7.48 (d, *J* = 8.0 Hz, 2H), 7.45 (d, *J* = 8.4 Hz, 2H), 7.25 (d, *J* = 6.4Hz, 2H), 4.55 (s, 2H), 2.40 (s, 3H). ^13^C-NMR (100 MHz, CDCl_3_) δ 141.48, 137.69,137.56, 137.58, 129.70, 129.61, 127.48, 127.08, 33.63, 21.27.

*4-methyl-4′-*(*4-methylbenzyl*)*-1,1′-biphenyl* (**7b**) [46]: White solid, MP: 77–78 °C (20.4 mg, 25%). ^1^H-NMR (400 MHz, CDCl_3_) δ 7.51–7.44 (m, 4H), 7.23 (dd, *J* = 8.0 3.6 Hz, 4H), 7.11 (s, 4H), 3.97 (s, 2H), 2.38 (s, 3H), 2.32 (s, 3H). ^13^C-NMR (100 MHz, CDCl_3_) δ 140.38, 139.02, 138.31, 138.16, 136.92, 135.74, 129.57, 129.34, 128.97, 127.13, 126.98, 41.30, 21.23, 21.17. 

*4-methyl-1,1′-biphenyl* (**7c**) [25]: White solid (46.8 mg, 92%). ^1^H-NMR (400 MHz, CDCl_3_) δ 7.57 (dd, *J* = 8.4, 1.6 Hz, 2H), 7.48 (d, *J* = 8.4 Hz, 2H), 7.41 (t, *J* = 7.6 Hz, 2H), 7.31 (t, *J* = 7.6 Hz, 1H), 7.24 (d, *J* = 8.0 Hz, 3H), 2.38 (s, 3H).

## 4. Conclusions

In conclusion, an efficient method for the selective Suzuki–Miyaura coupling of *o*-(or *m*-, or *p*-)chloromethyl bromobenzene with arylboronic acids was described. This Pd-catalyzed highly selective coupling reaction of the C(sp^2^)–Br bond was achieved by using PCy_3_·HBF_4_ as the ligand and Cs_2_CO_3_ as the base in a mixture of toluene/water (10:1). A series of chloromethyl-1,1′-biphenyl compounds were obtained in moderate-to-excellent yields. Importantly, the catalytic system exhibited a wide substrate scope and good functional group tolerance. Moreover, this protocol was extended to the one-pot dual arylation of 1-bromo-4-(chloromethyl)benzene, affording numerous unsymmetrical methylene-linked biaryl derivatives.

## Figures and Tables

**Table 1:**
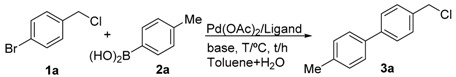
Optimized reaction conditions ^a^.

Entry	Pd(OAc)_2_	PCy_3_∙HBF_4_	Base/Equiv.	T/°C	Yield/% ^b^
1	2 mol %	4 mol %	NEt_3_	80	(37) ^c^
2	2 mol %	4 mol %	NaOH	80	(14) ^c^
3	2 mol %	4 mol %	K_3_PO_4_∙3H_2_O	80	(69) ^c^
4	2 mol %	4 mol %	K_2_CO_3_	80	(84) ^c^
5	2 mol %	4 mol %	Cs_2_CO_3_	80	99(97) ^c^
6	2 mol %	PPh_3_/4 mol %	Cs_2_CO_3_	80	0
7	2 mol %	4 mol %	Cs_2_CO_3_	60	74
8	1 mol %	2 mol %	Cs_2_CO_3_	80	99
9	0.5 mol %	1 mol %	Cs_2_CO_3_	80	99
10	0.2 mol %	0.4 mol %	Cs_2_CO_3_	80	99
11	0.1 mol %	0.2 mol %	Cs_2_CO_3_	80	23

^a^ Reaction conditions: 1-bromo-4-(chloromethyl)benzene 0.30 mmol, *p*-tolylboronic acid 0.33 mmol, base 2 equiv., 1.0 mL toluene and 0.1 mL H_2_O, 2 h, Ar protection. ^b^ Gas chromatography-mass spectrometry (GC–MS) yield. ^c^ Isolated yield.

**Table 2:** Selective coupling reaction of 1-bromo-4-(chloromethyl)benzene with arylboronic acid ^a^.

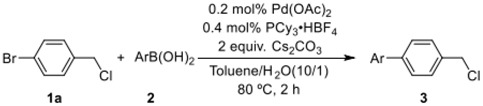		
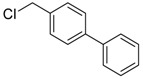 **3b**, 79%	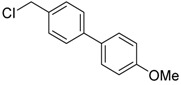 **3c**, 87%	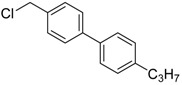 **3d**, 83%
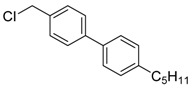 **3e**, 93%	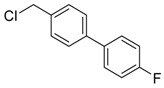 **3f**, 83%	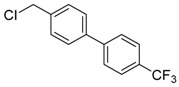 **3g**, 75%
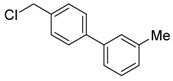 **3h**, 98%	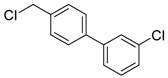 **3i**, 73%	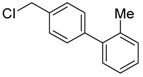 **3j**, 90%
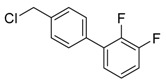 **3k**, 47%	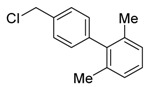 **3l**, 50%	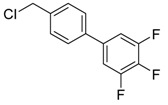 **3m**, 73%
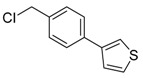 **3n**, 77%	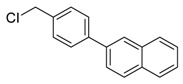 **3o**, 86%	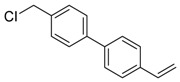 **3p**, 57%

^a^ Reaction conditions: 1-bromo-4-(chloromethyl)benzene 0.30 mmol, arylboronic acid 0.33 mmol, 0.2 mol % Pd(OAc)_2_, 0.4 mol % PCy_3_∙HBF_4_, 2 equiv. Cs_2_CO_3_, 1.0 mL toluene and 0.1 mL H_2_O, 80 °C, 2 h, Ar protection. The yields of isolated products are given.

**Table 3:** Selective coupling reaction of arylboronic acid with 1-bromo-3-(chloromethyl)benzene ^a^.

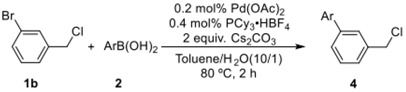			
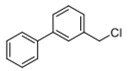 **4a**, 95%	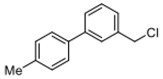 **4b**, 90%	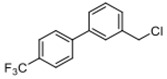 **4c**, 92%	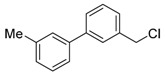 **4d**, 93%
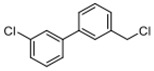 **4e**, 92%	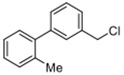 **4f**, 87%	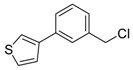 **4g**, 95%	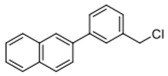 **4h**, 73%

^a^ Reaction conditions: 1-bromo-3-(chloromethyl)benzene 0.30 mmol, arylboronic acid 0.33 mmol, 0.2 mol % Pd(OAc)_2_, 0.4 mol % PCy_3_∙HBF_4_, 2 equiv. Cs_2_CO_3_, 1.0 mL toluene and 0.1 mL H_2_O, 80 °C, 2 h, Ar protection. The yields of isolated products are given.

**Table 4:** Selective coupling reaction of arylboronic acid with 1-bromo-2-(chloromethyl)benzene ^a.^

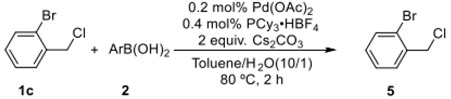			
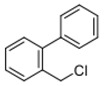 **5a**, 97%	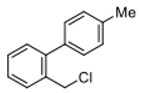 **5b**, 95%	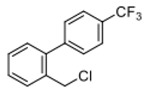 **5c**, 89%	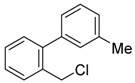 **5d**, 92%
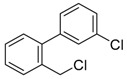 **5e**, 88%	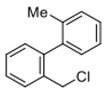 **5f**, 87%	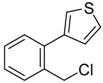 **5g**, 95%	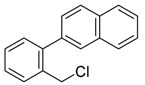 **5h**, 80%

^a^ Reaction conditions: 1-bromo-3-(chloromethyl)benzene 0.30 mmol, arylboronic acid 0.33 mmol, 0.2 mol % Pd(OAc)_2_, 0.4 mol % PCy_3_∙HBF_4_, 2 equiv. Cs_2_CO_3_, 1.0 mL toluene and 0.1 mL H_2_O, 80 °C, 2 h, Ar protection. The yields of isolated products are given.

**Table 5:** One-pot dual arylations of 1-bromo-4-(chloromethyl)benzene ^a.^

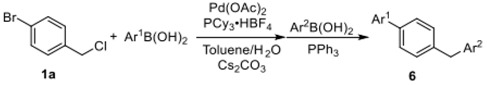	
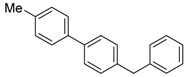 **6a**, 78%	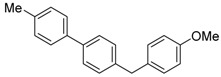 **6b**, 96%
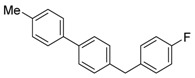 **6c**, 89%	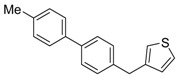 **6d**, 57%

^a^ Reaction conditions: (1) 1-bromo-4-(chloromethyl)benzene 0.30 mmol, arylboronic acid 0.33 mmol, 2 mol % Pd(OAc)_2_, 0.4 mol % PCy_3_∙HBF_4_, 5 equiv. Cs_2_CO_3_, 1.0 mL toluene and 0.1 mL H_2_O, 80 °C, 2 h, Ar protection; (2) 4.0 mol % PPh_3_, arylboronic acid 0.33 mmol, 80 °C, 5 h, Ar protection. The yields of isolated products are given.

## Supplementary Materials

Supplementary materials are available online. Figures S2–S46: ^1^H-, ^13^C-NMR, and HRMS of products.
