# Supplementary material for: Pd-Catalyzed, Highly Selective C(sp2)-Br Bond Coupling Reactions of o-(or m-, or p-) Chloromethyl Bromobenzene with Arylboronic Acids
Source: Molecules. 2018 Feb 15;23(2):433. doi: 10.3390/molecules23020433 (PMC6017369; doi:10.3390/molecules23020433)

Supporting Information for

Pd-Catalyzed Highly Selective C(sp^2)-Br Bond Coupling Reactions of *o*-(or *m*-, or *p*-) Chloromethyl Bromobenzene with Arylboronic Acids

Ming-ming Pei, Ping Liu*, Yan Liu, Xin-ming Lv, Xiao-wei Ma*, and Bin Dai

School of Chemistry and Chemical Engineering/Key Laboratory for Green Processing of Chemical Engineering of Xinjiang Bingtuan, Shihezi University, Shihezi 832003, China; peimming@163.com; liuyan1979810@aliyun.com; ciqlxm@163.com; db_tea@shzu.edu.cn.

* Correspondence: e-mail: liuping1979112@aliyun.com, mxw_tea@shzu.edu.cn. Tel.: +86 0993 2057213.

Copies of ^{1}H NMR, ^{13}C NMR and HRMS Spectra…………S2–S45

4-(chloromethyl)-4'-methyl-1,1'-biphenyl[3a]

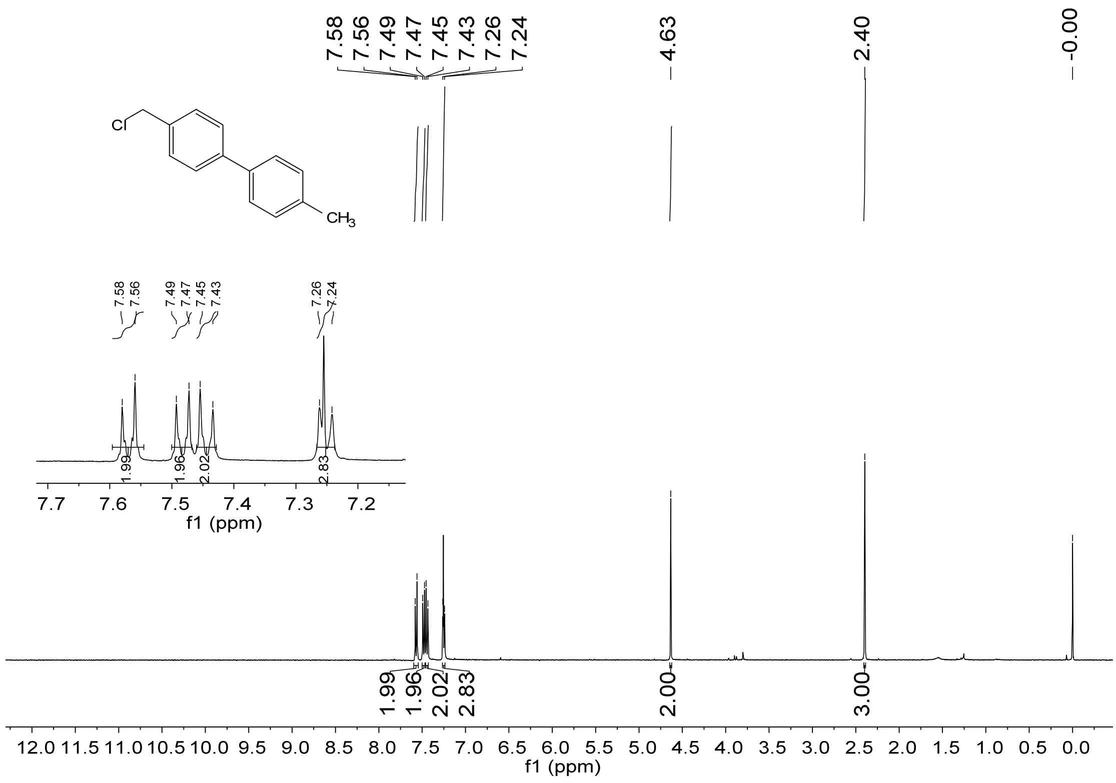

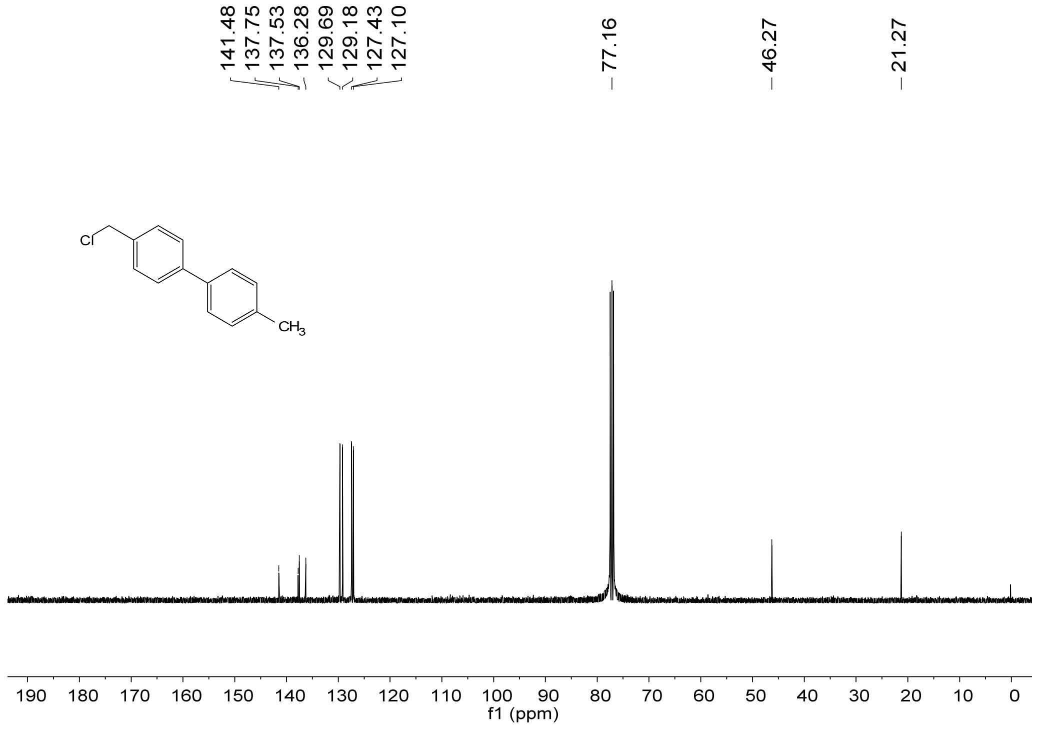

4-(chloromethyl)-1,1'-biphenyl[3b]

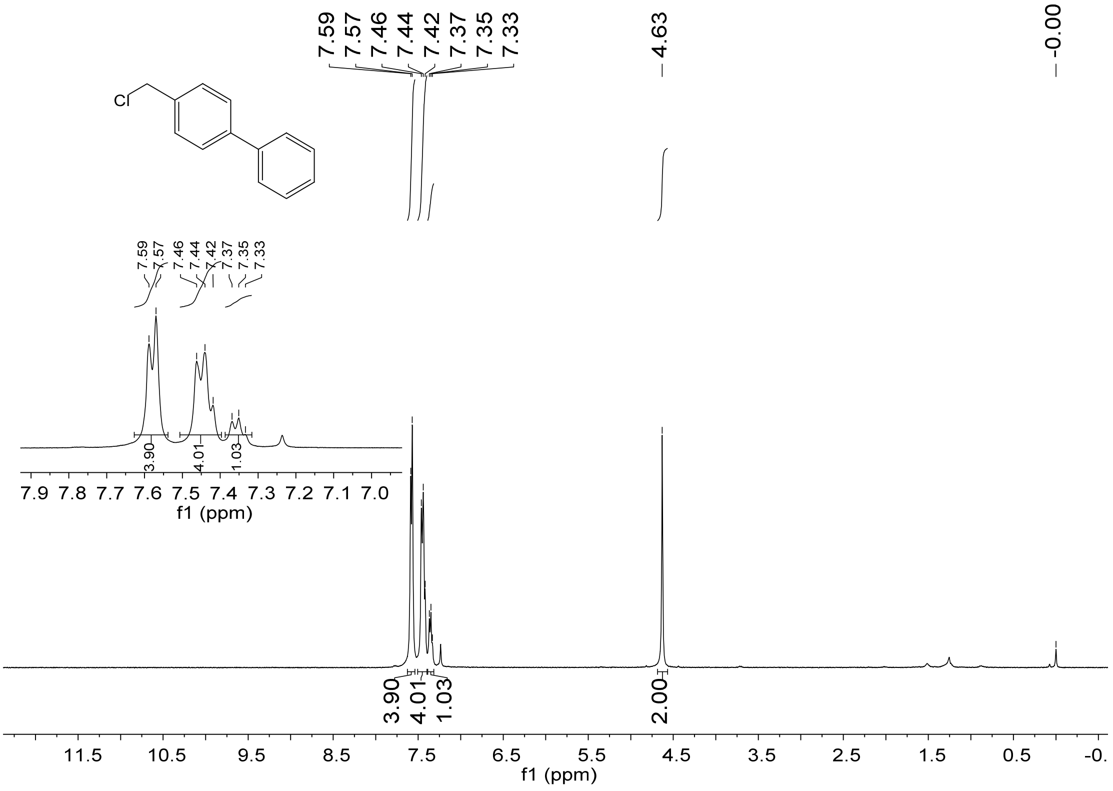

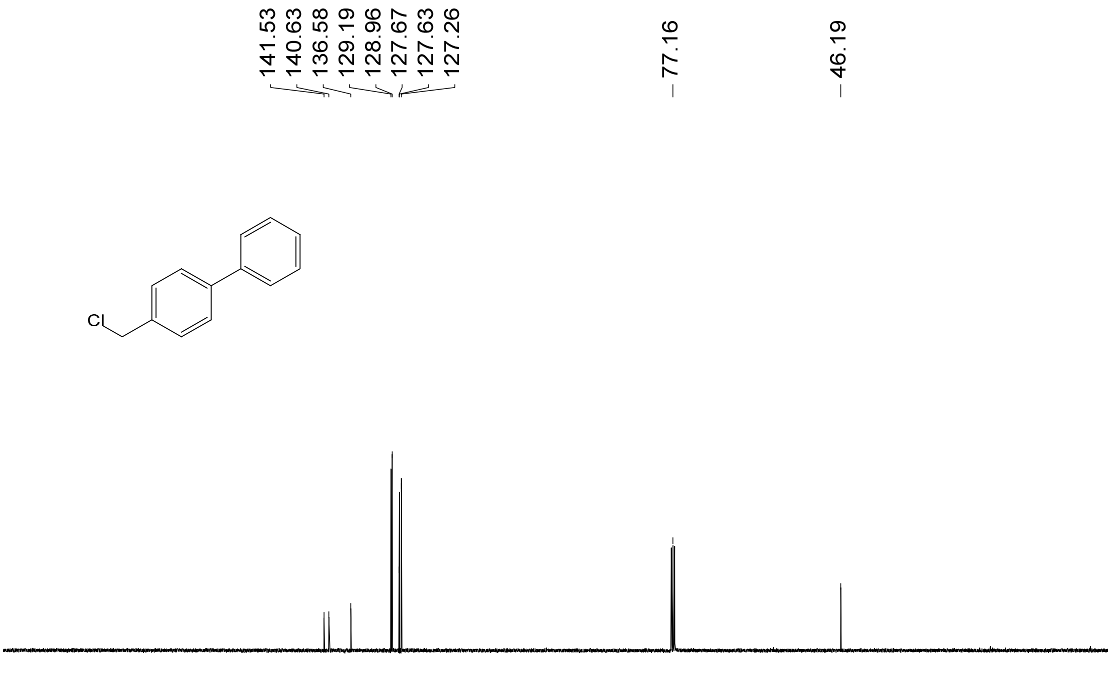

4-(chloromethyl)-4'-methoxy-1,1'-biphenyl[3c]

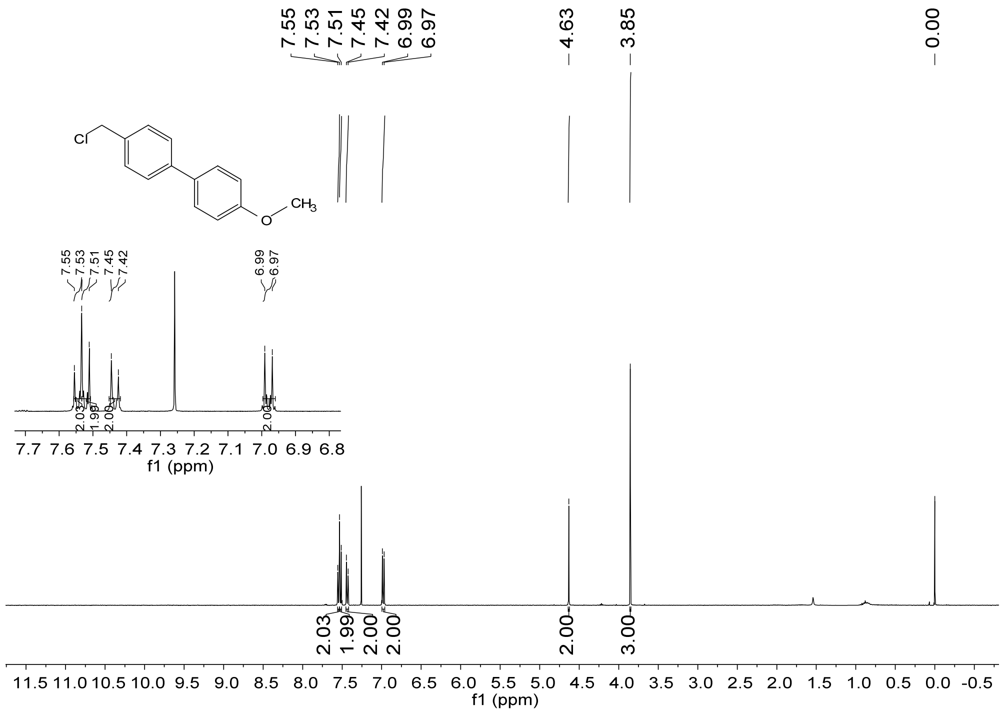

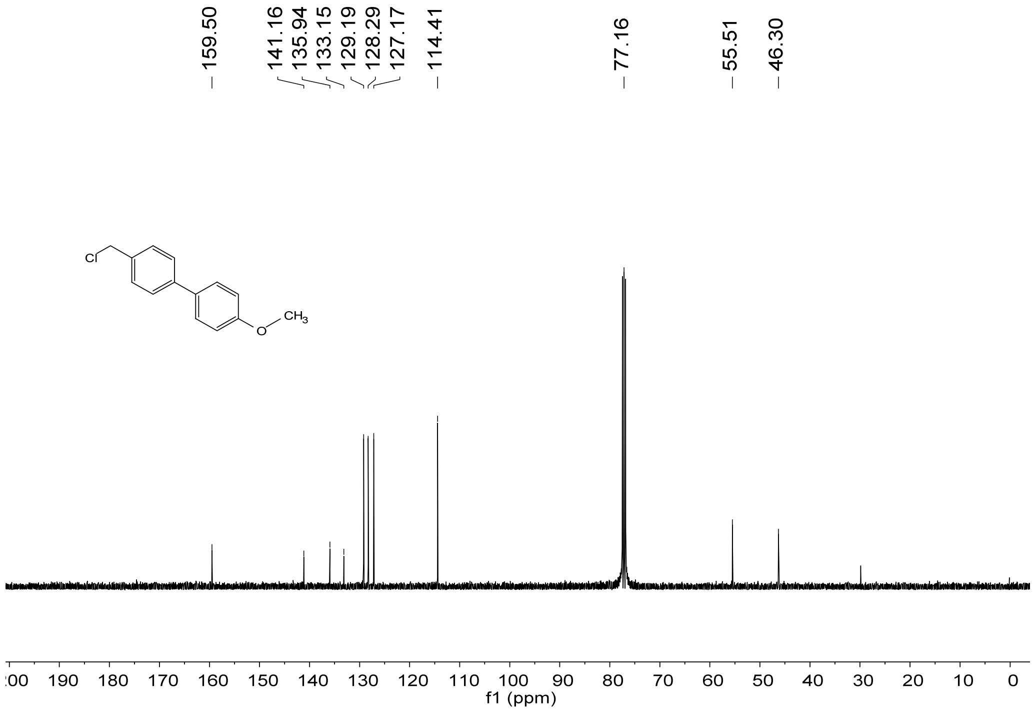

4-(chloromethyl)-4'-propyl-1,1'-biphenyl[3d]

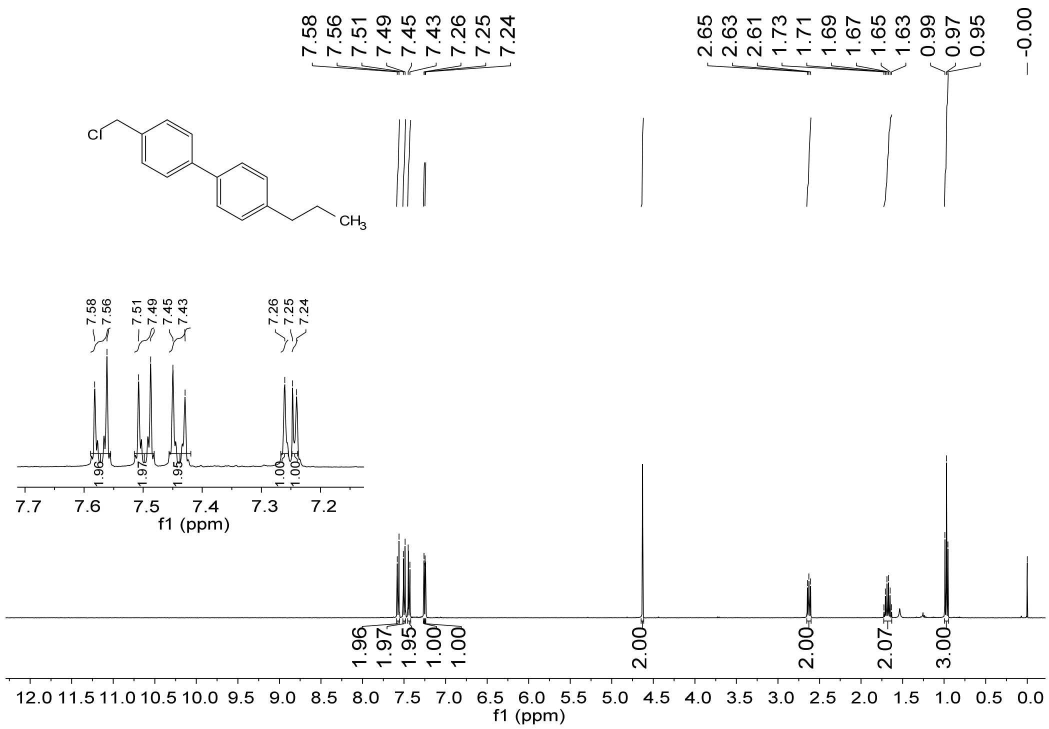

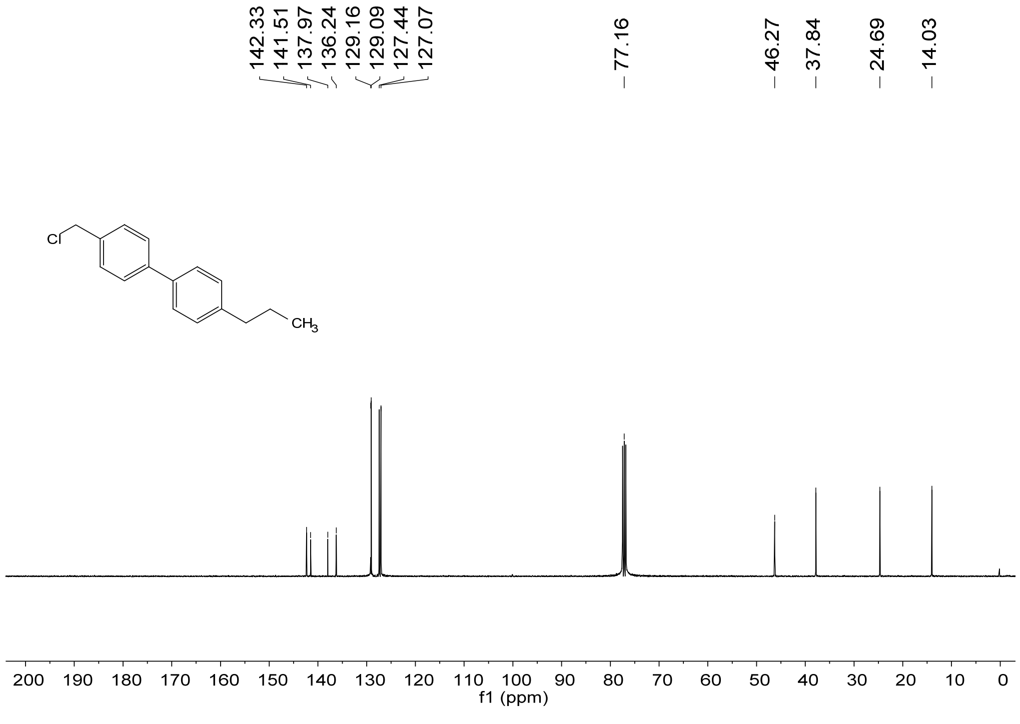

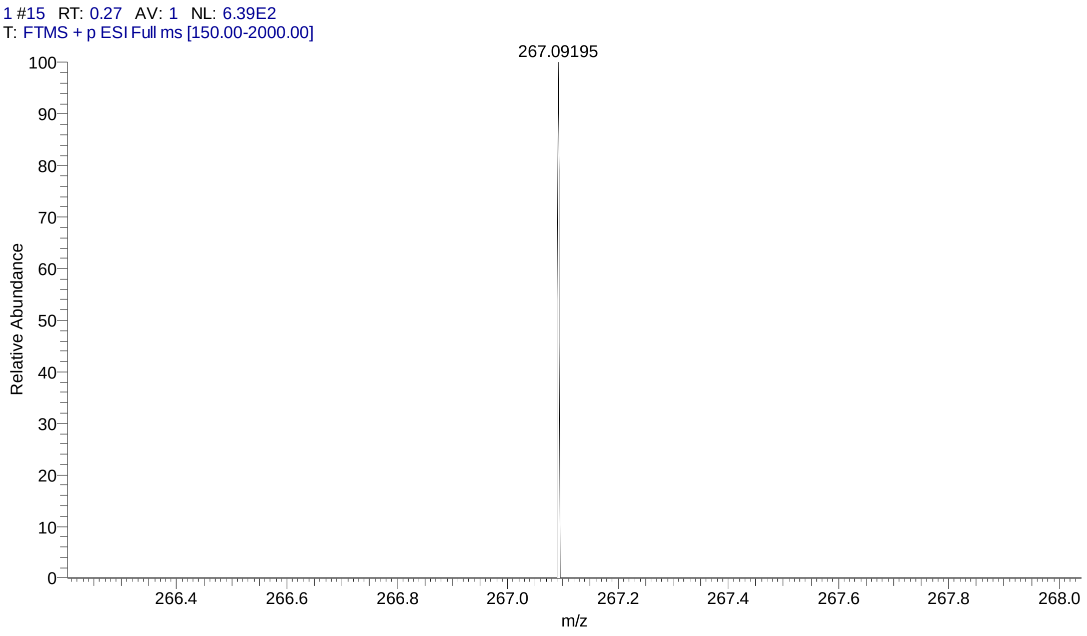

4-(chloromethyl)-4'-pentyl-1,1'-biphenyl[3e]

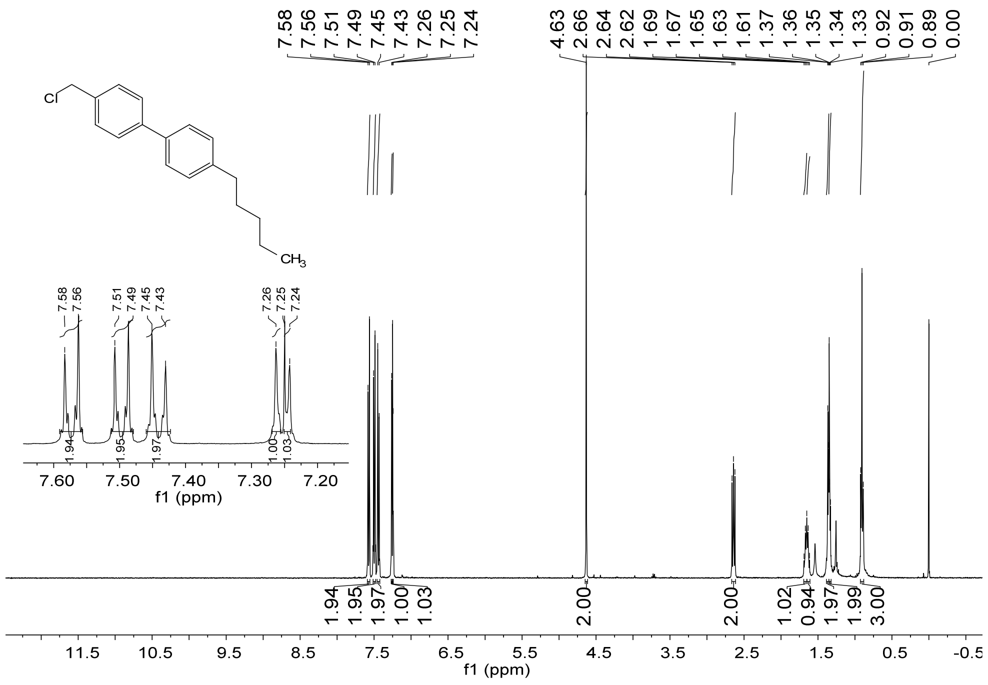

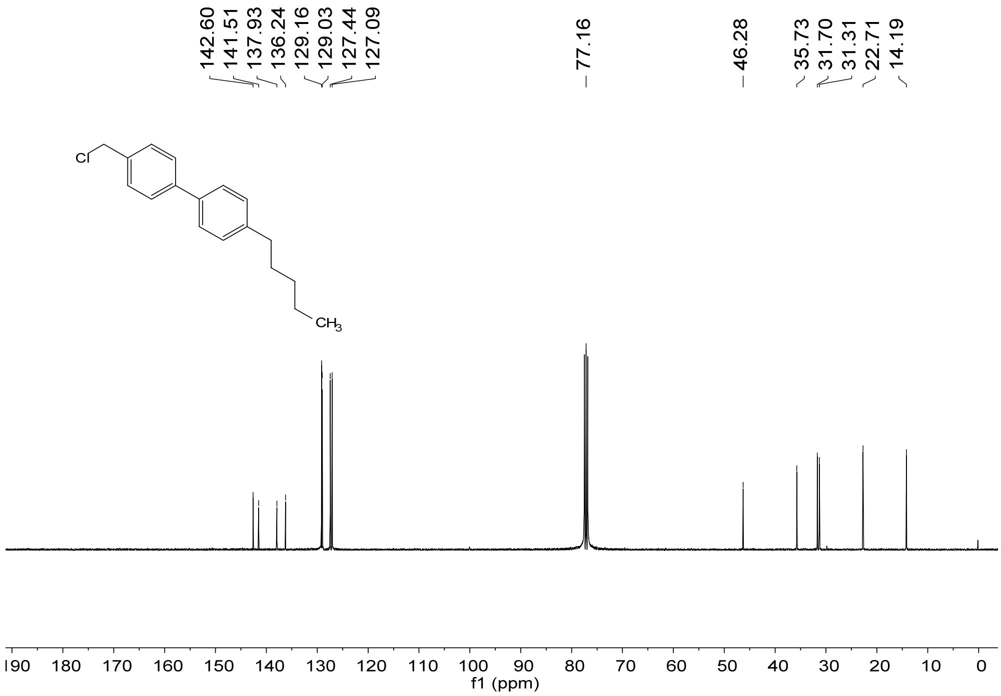

4-(chloromethyl)-4'-fluoro-1,1'-biphenyl[3f]

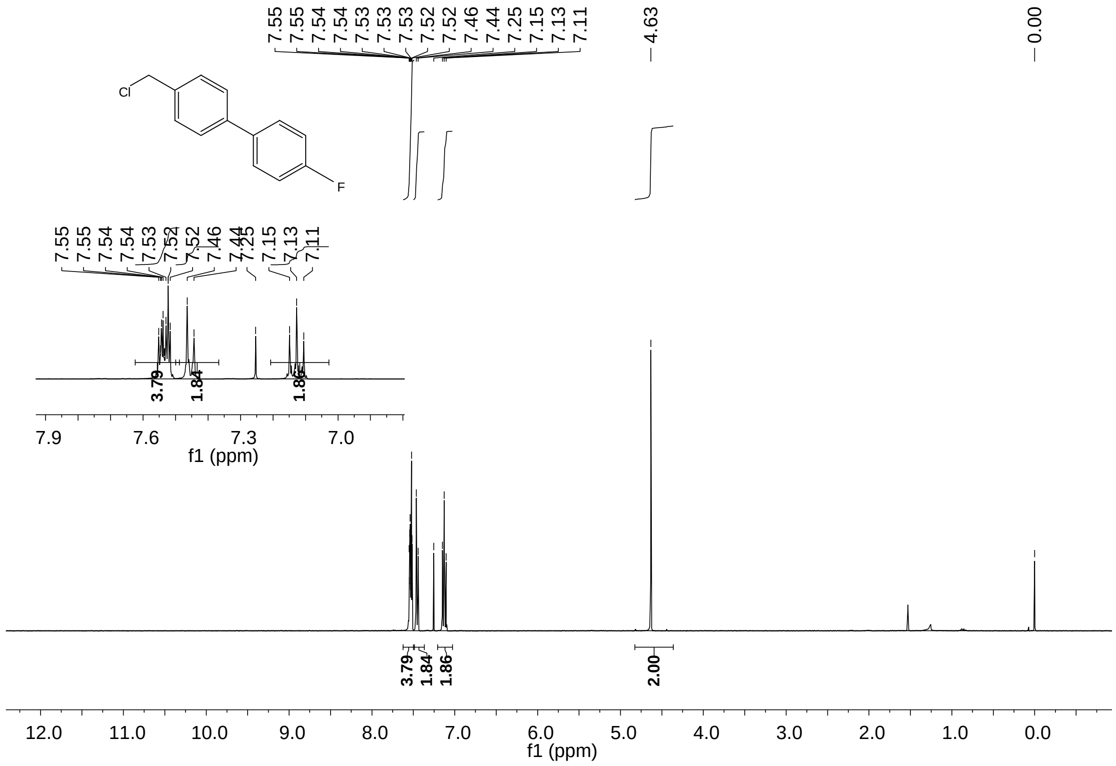

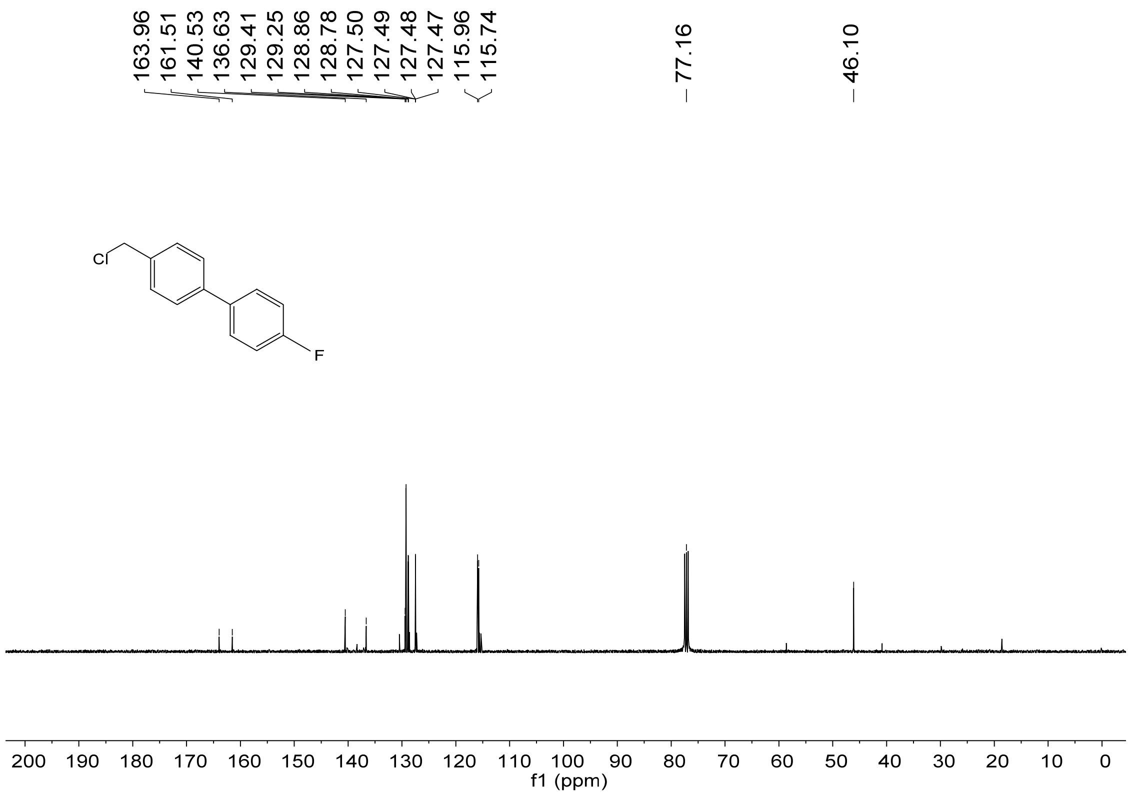

4-(chloromethyl)-4'-(trifluoromethyl)-1,1'-biphenyl[3g]

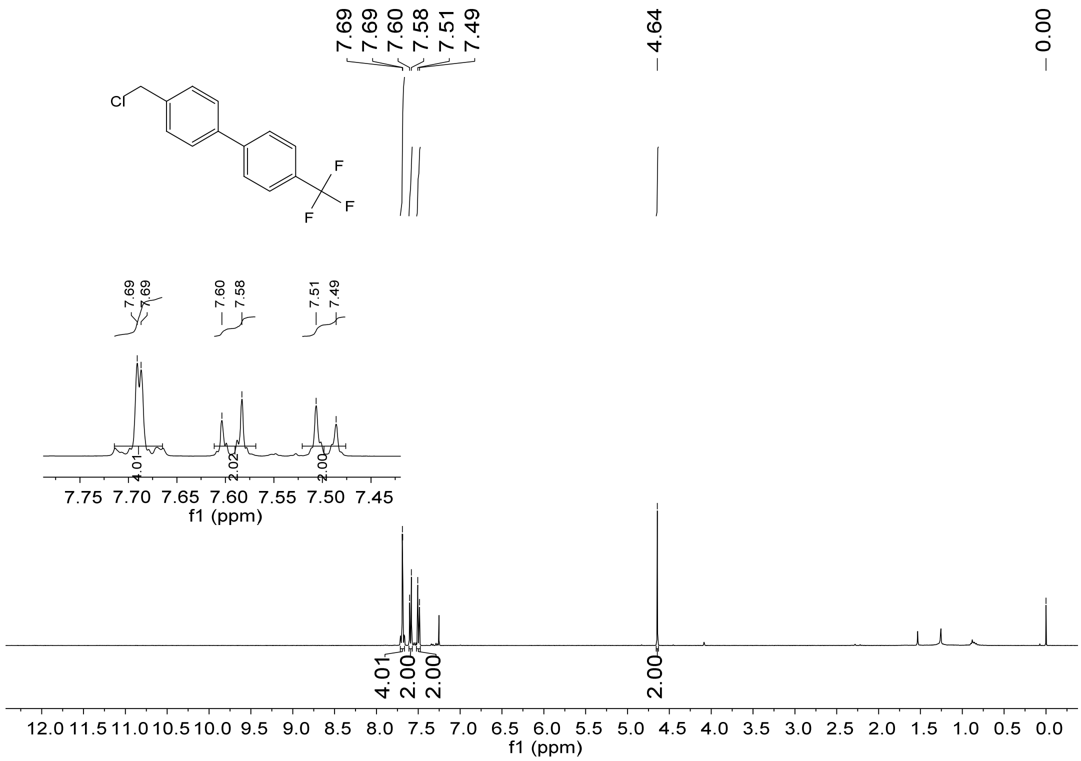

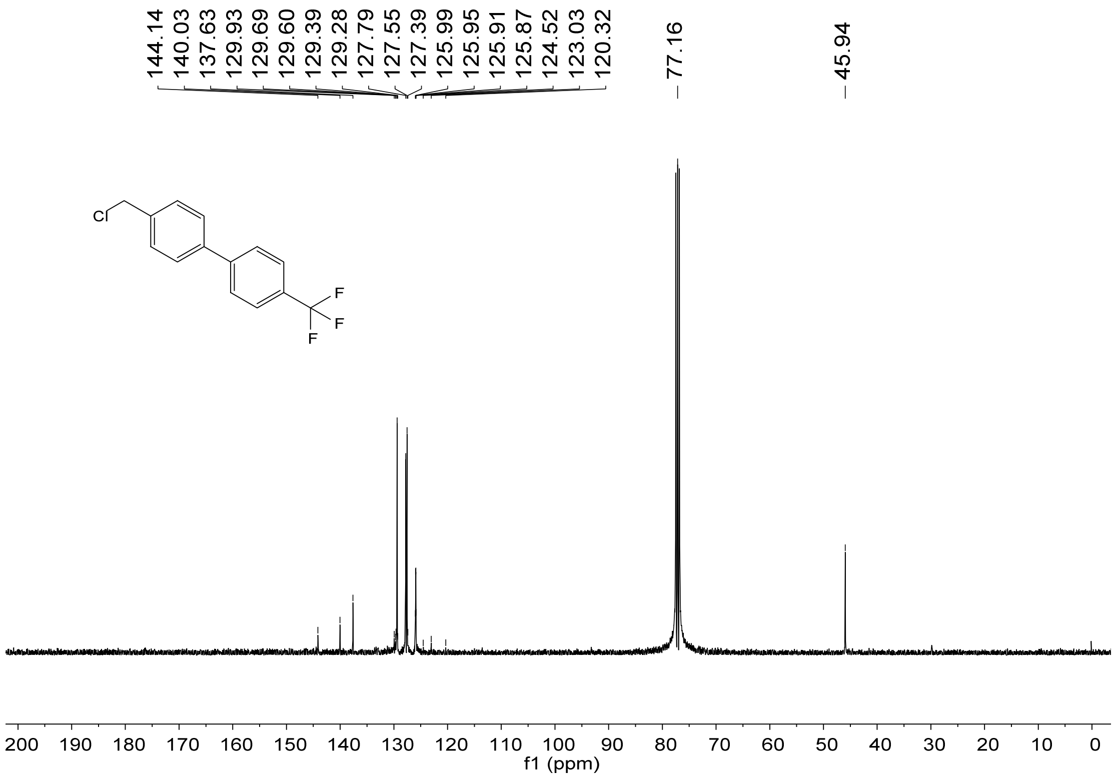

4'-(chloromethyl)-3-methyl-1,1'-biphenyl[3h]

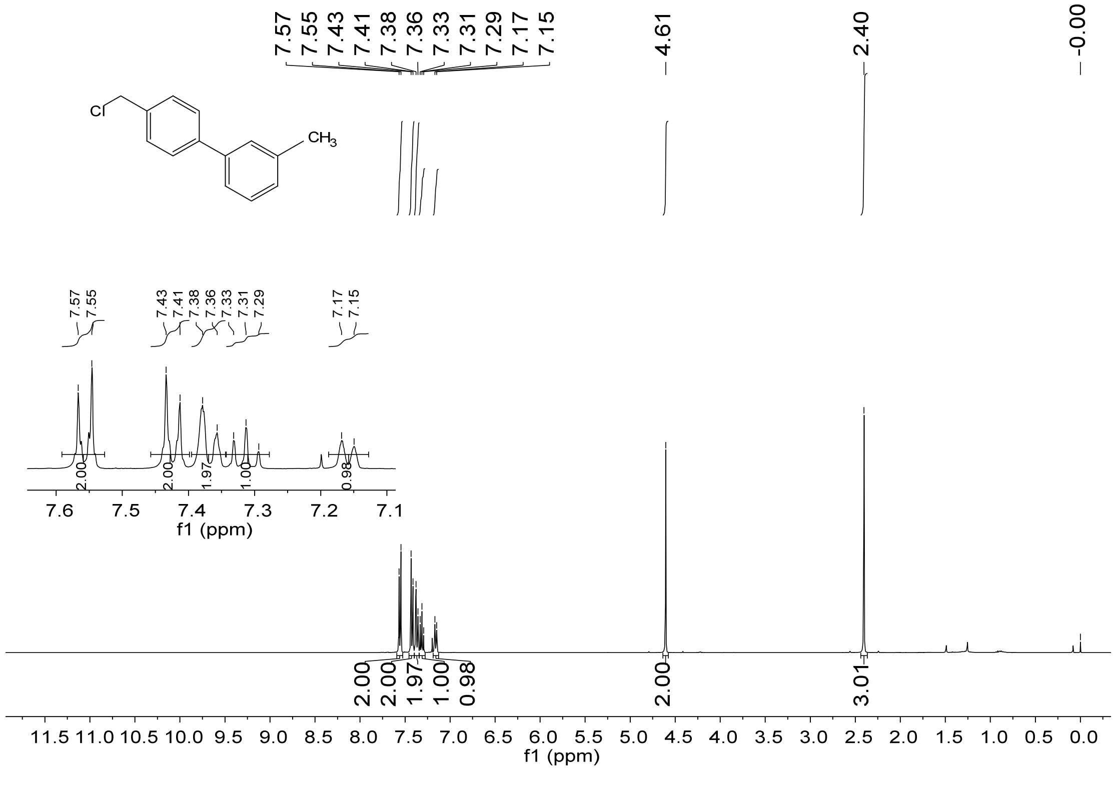

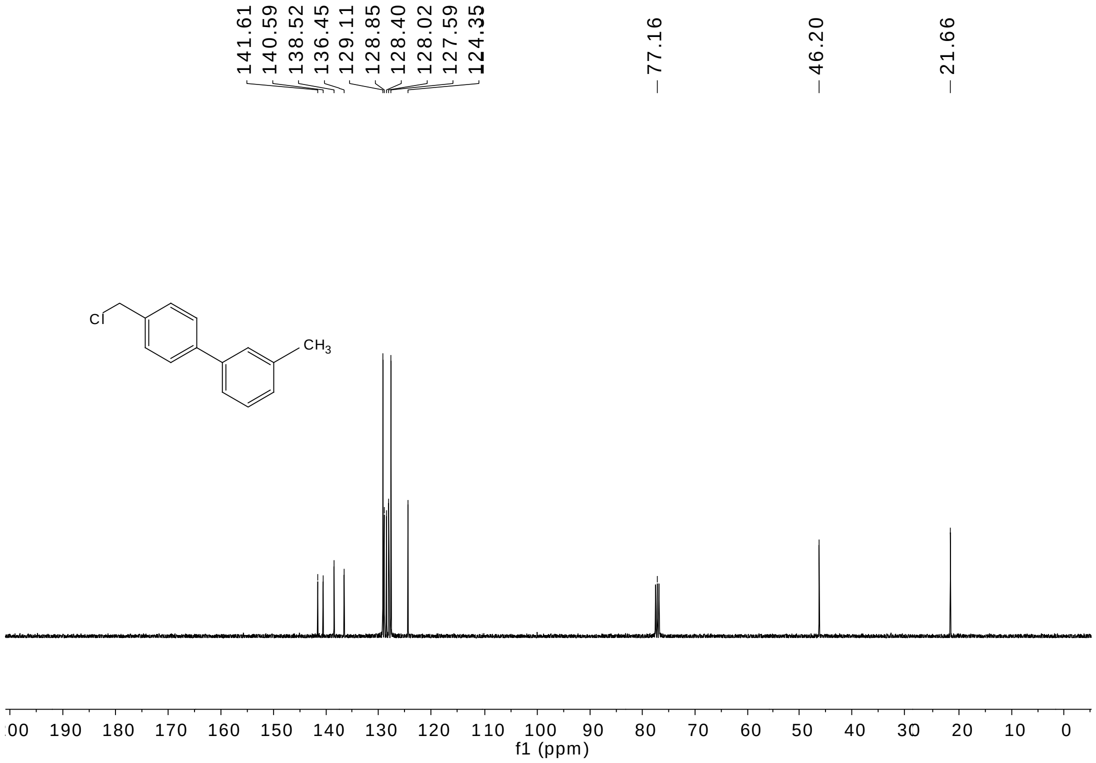

Elemental Composition Report **Page 1**

Single Mass Analysis (displaying only valid results)
Tolerance = 10.0 PPM / DBE: min = -1.5, max = 50.0
Element prediction: Off

Monoisotopic Mass, Odd and Even Electron Ions
15 formula(e) evaluated with 1 results within limits (up to 50 best isotopic matches for each mass)
Elements Used:
C: 0-15 H: 0-15 F: 0-3 Cl: 0-2
default file
HR-3-1 1943 (12.058) Cm (1943-(1934:1938+1965:1968)) TOF MS EI+ 9.24e+002

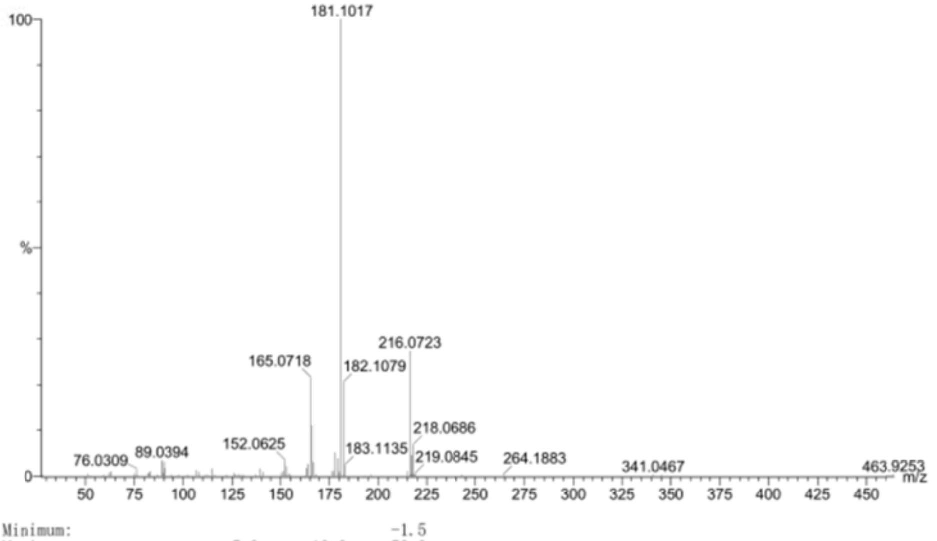

Mass	Calc. Mass	mDa	PPM	DBE	i-FIT	Formula
Minimum:				-1.5		
Maximum:		5.0	10.0	50.0		
216.0723	216.0706	1.7	7.9	8.0	2.7	C14 H13 Cl

3-chloro-4'-(chloromethyl)-1,1'-biphenyl[3i]

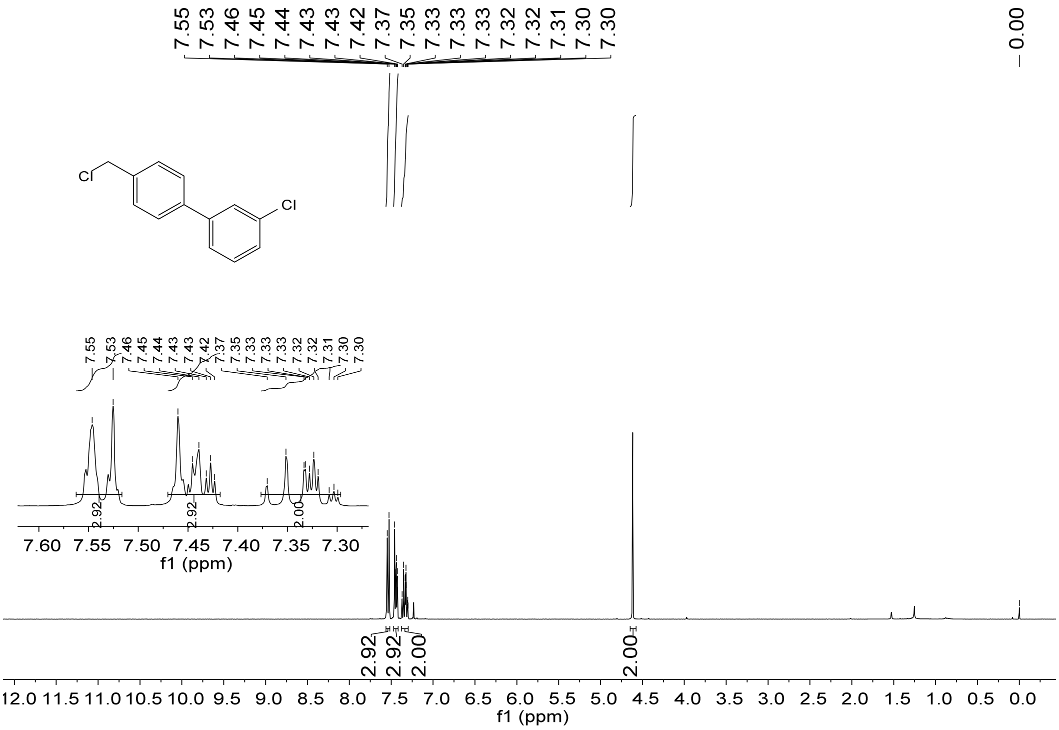

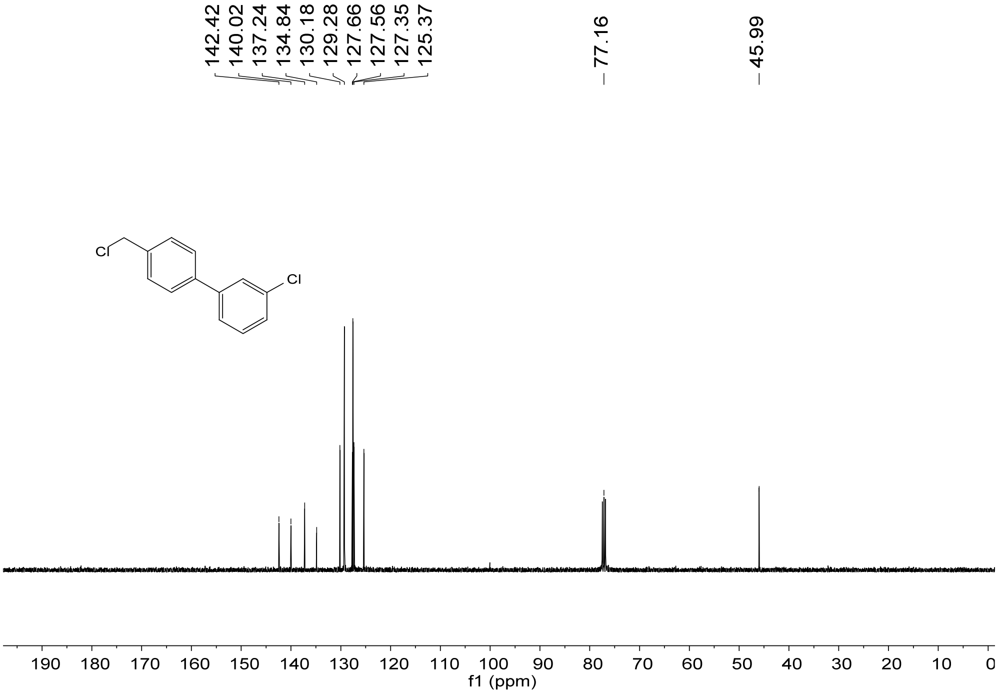

Elemental Composition Report **Page 1**

Single Mass Analysis (displaying only valid results)
Tolerance = 10.0 PPM / DBE: min = -1.5, max = 50.0
Element prediction: Off

Monoisotopic Mass, Odd and Even Electron Ions
14 formula(e) evaluated with 1 results within limits (up to 50 best isotopic matches for each mass)
Elements Used:
C: 0-15 H: 0-15 F: 0-3 Cl: 0-2
default file
HR-3-1 2045 (12.602) Cm (2045-(2028:2036+2057:2068)) TOF MS EI+ 1.30e+003

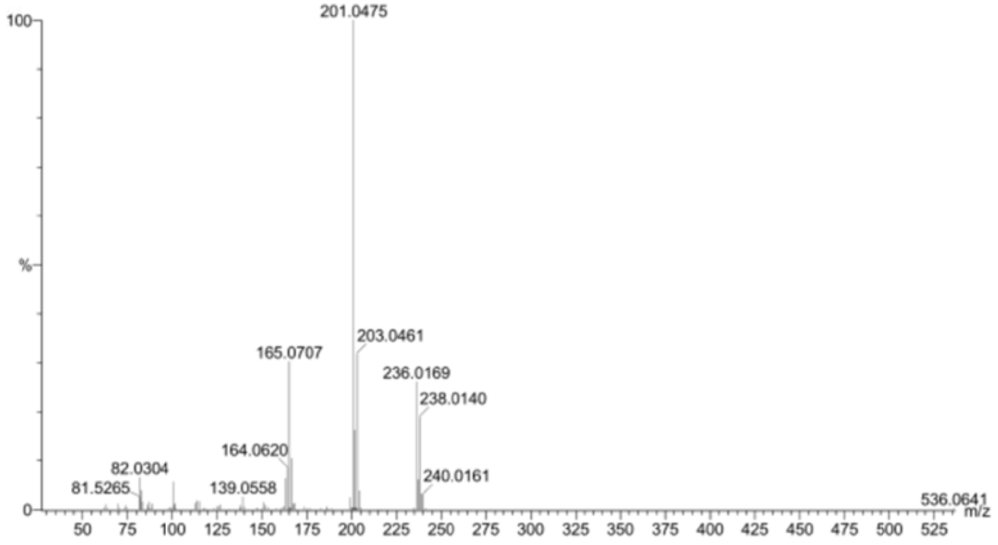

Minimum:				-1.5		
Maximum:		5.0	10.0	50.0		
Mass	Calc. Mass	mDa	PPM	DBE	i-FIT	Formula
236.0169	236.0160	0.9	3.8	8.0	5.4	C13 H10 Cl2

4'-(chloromethyl)-2-methyl-1,1'-biphenyl[3j]

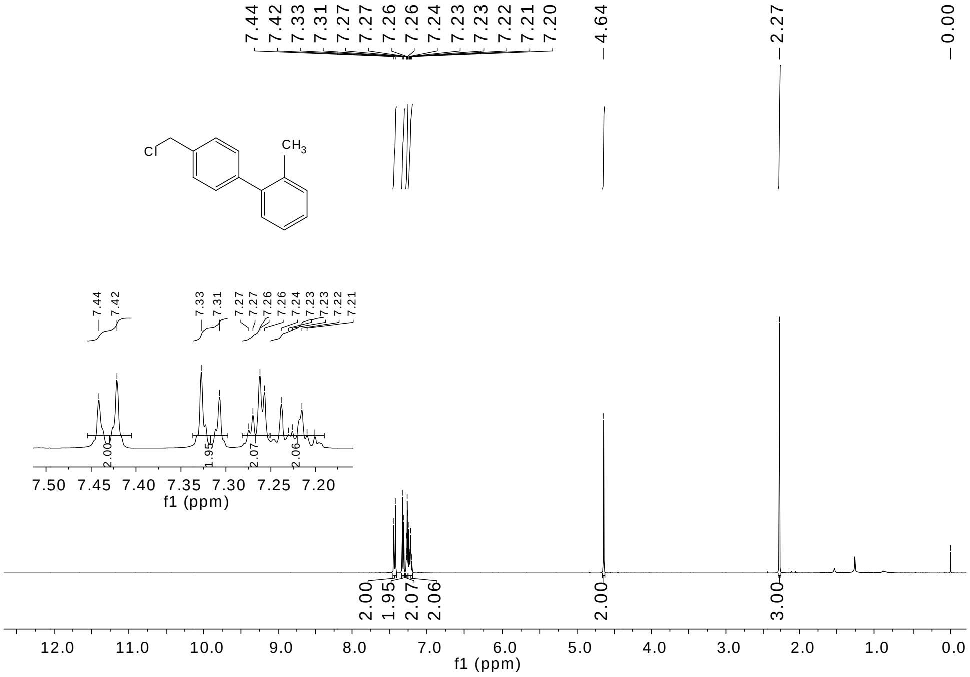

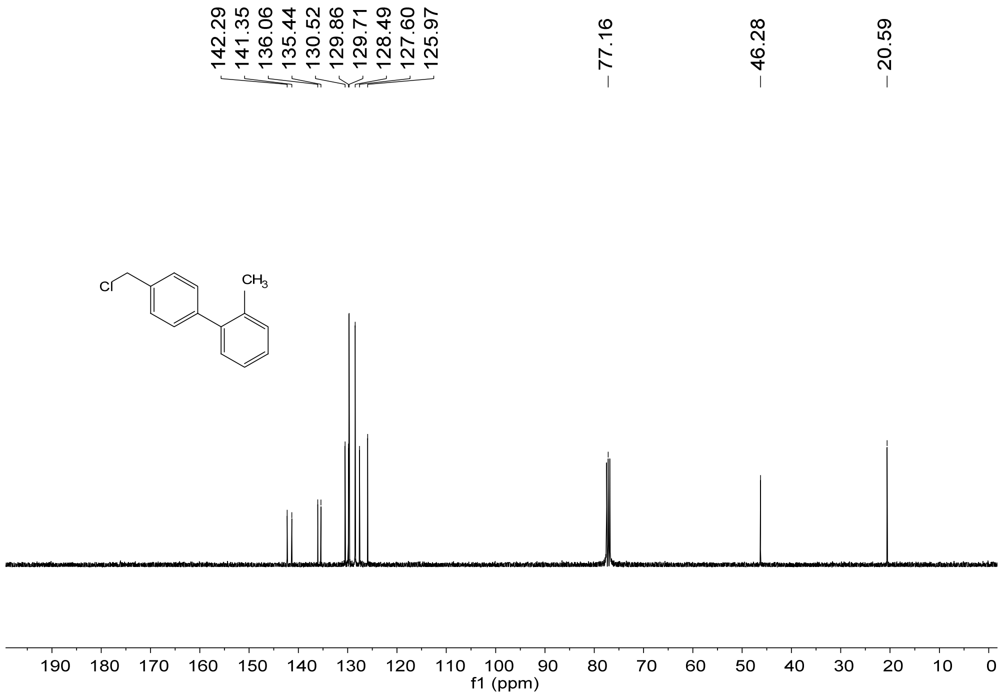

4'-(chloromethyl)-2,3-difluoro-1,1'-biphenyl[3k]

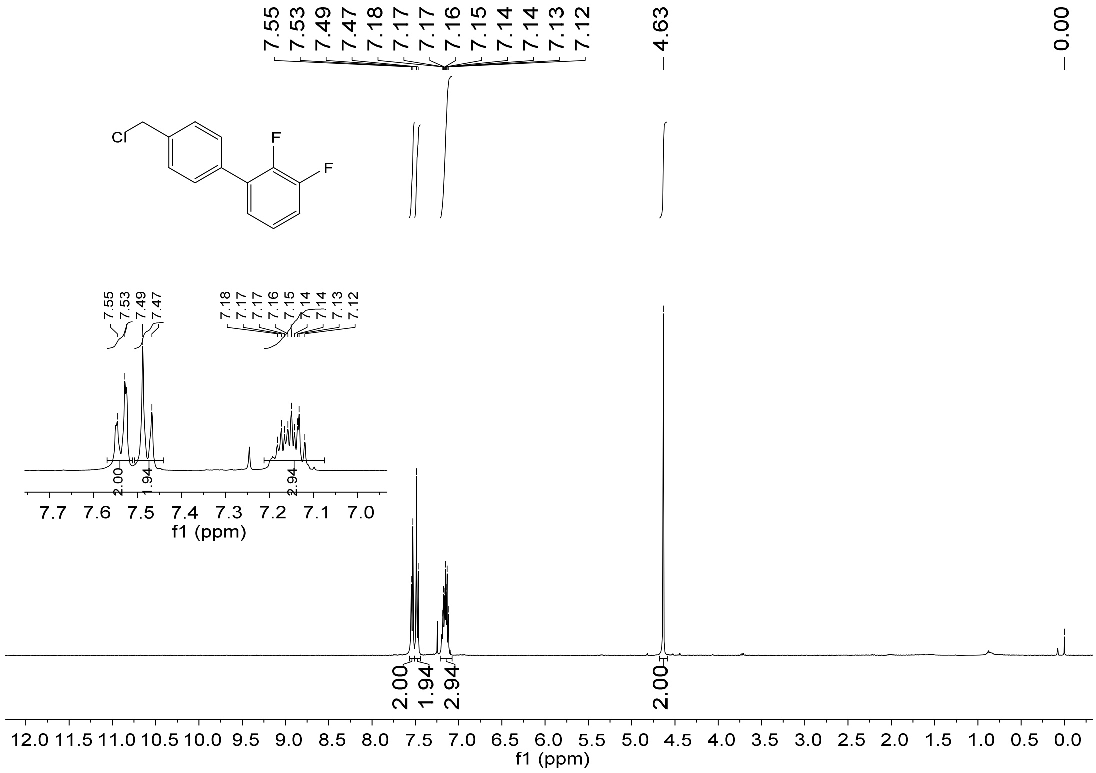

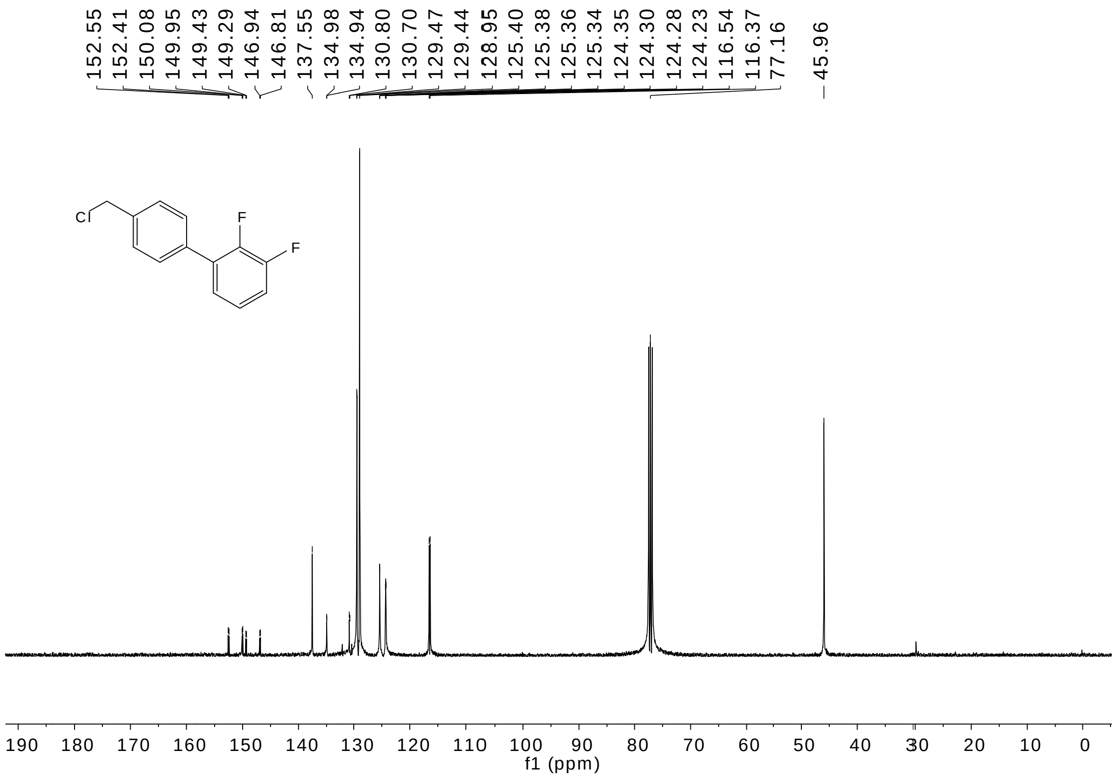

Elemental Composition Report

Page 1

Single Mass Analysis (displaying only valid results)

Tolerance = 10.0 PPM / DBE: min = -1.5, max = 50.0
Element prediction: Off

Monoisotopic Mass, Odd and Even Electron Ions
13 formula(e) evaluated with 1 results within limits (up to 50 best isotopic matches for each mass)
Elements Used:
C: 0-15 H: 0-15 Cl: 0-2 F: 0-3

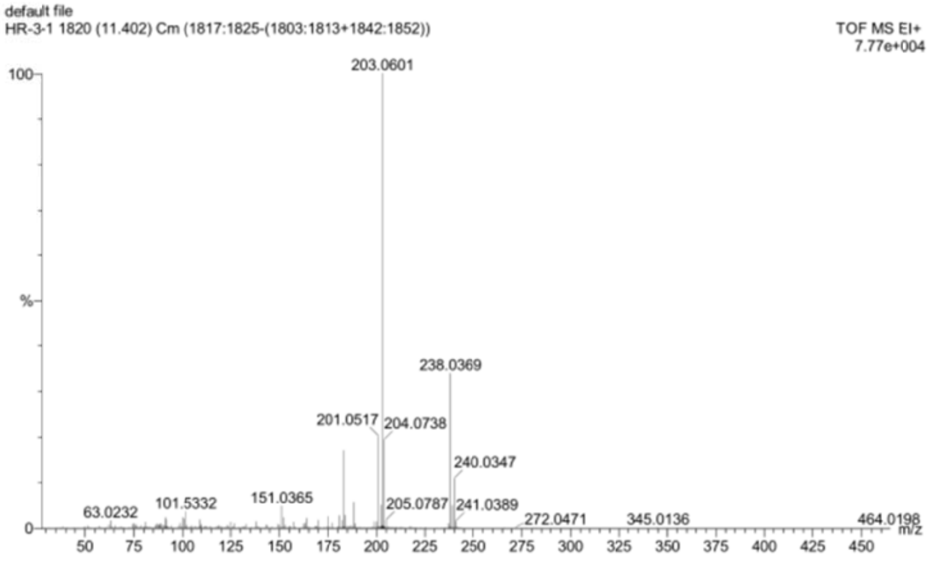

Minimum:				-1.5		
Maximum:		5.0	10.0	50.0		
Mass	Calc. Mass	mDa	PPM	DBE	i-FIT	Formula
238.0369	238.0361	0.8	3.4	8.0	6.4	C13 H9 Cl F2

4'-(chloromethyl)-2,6-dimethyl-1,1'-biphenyl[3l]

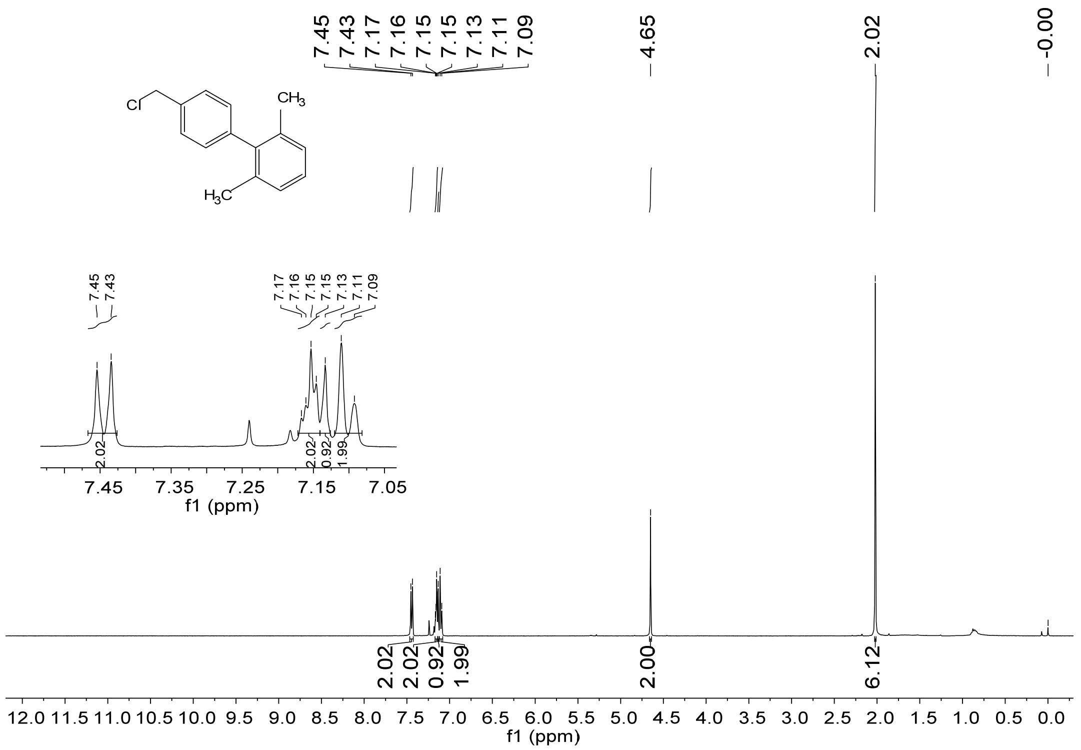

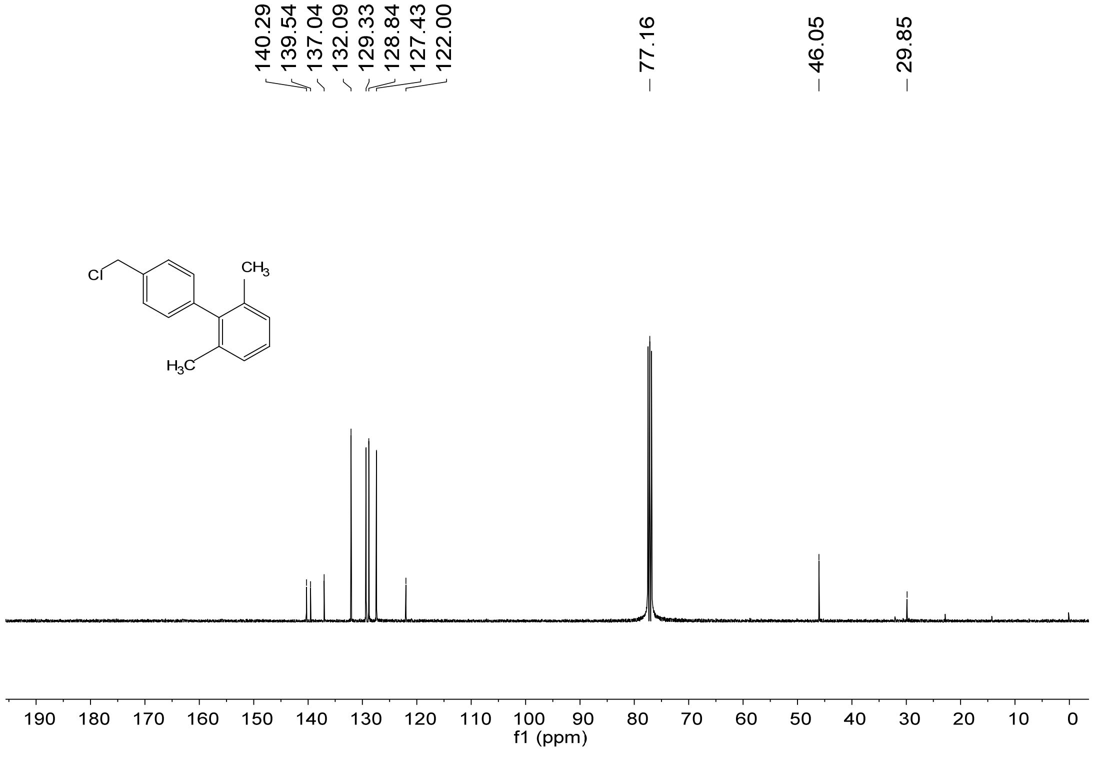

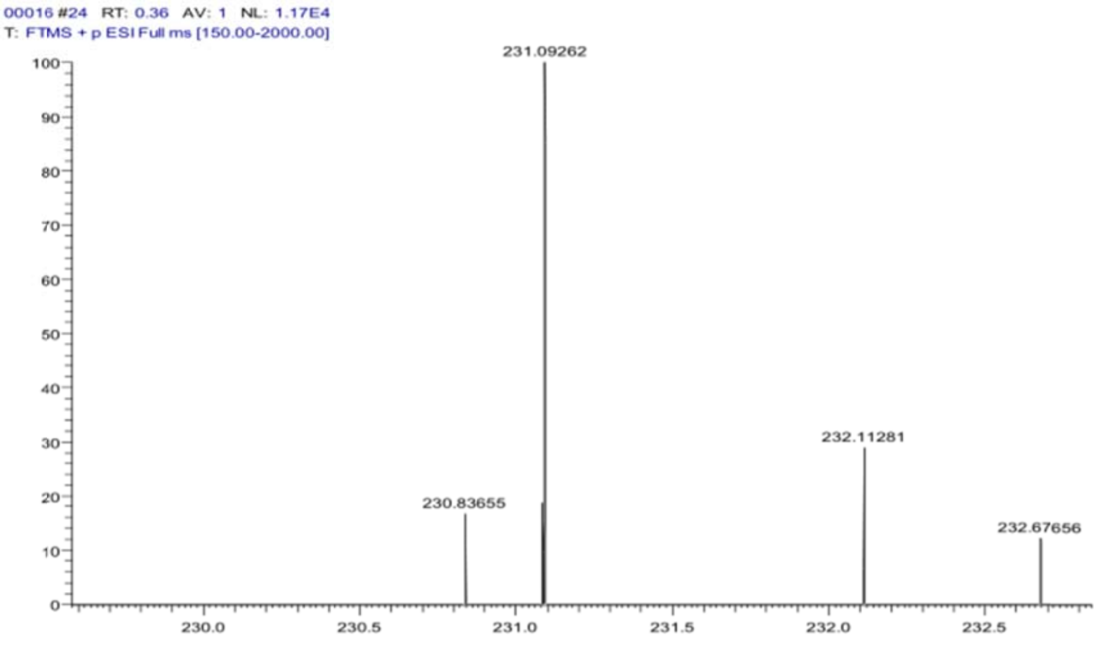

4'-(chloromethyl)-3,4,5-trifluoro-1,1'-biphenyl[3m]

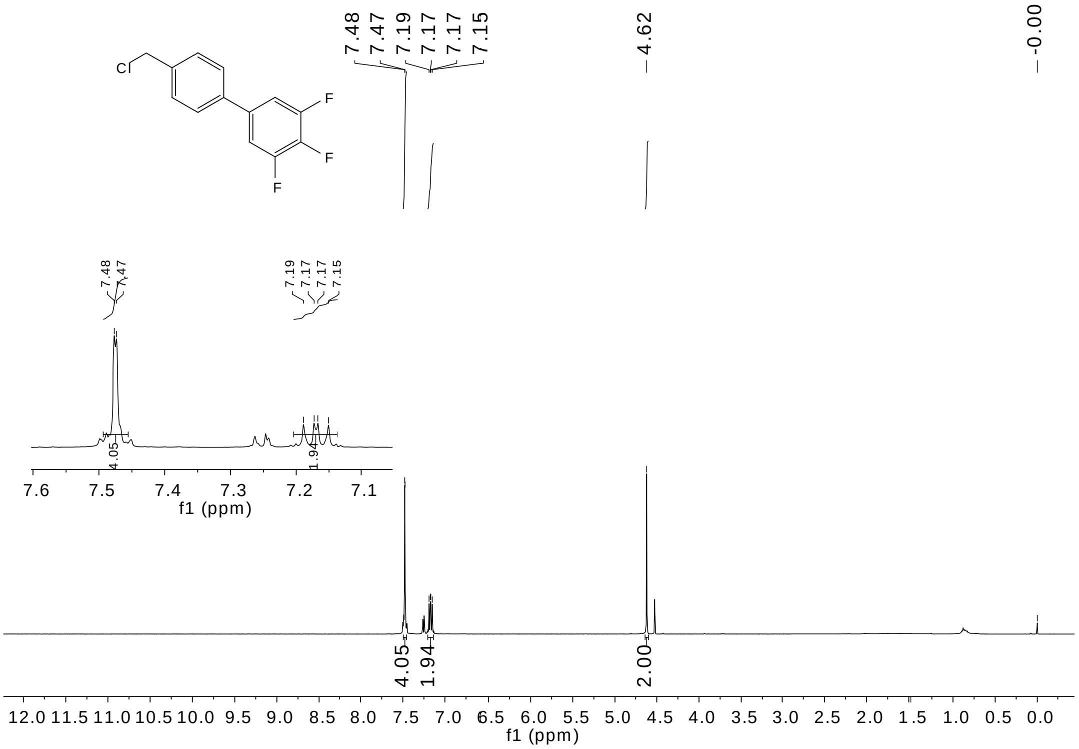

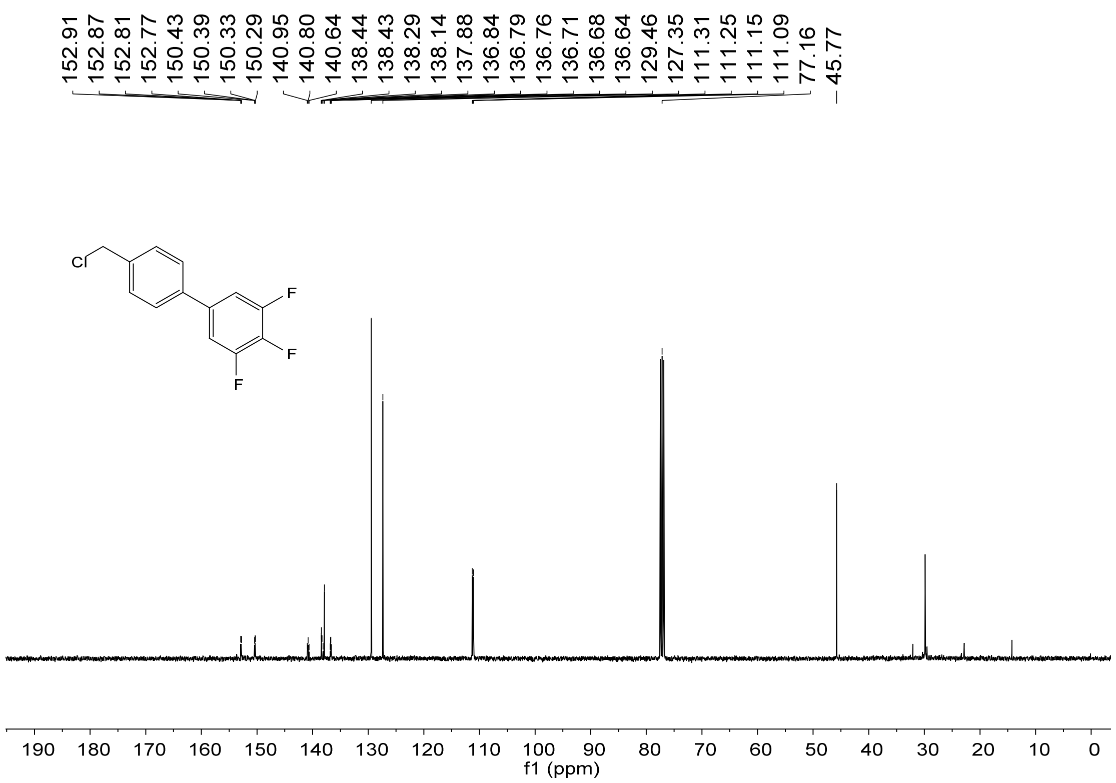

3-(4-(chloromethyl)phenyl)thiophene[3n]

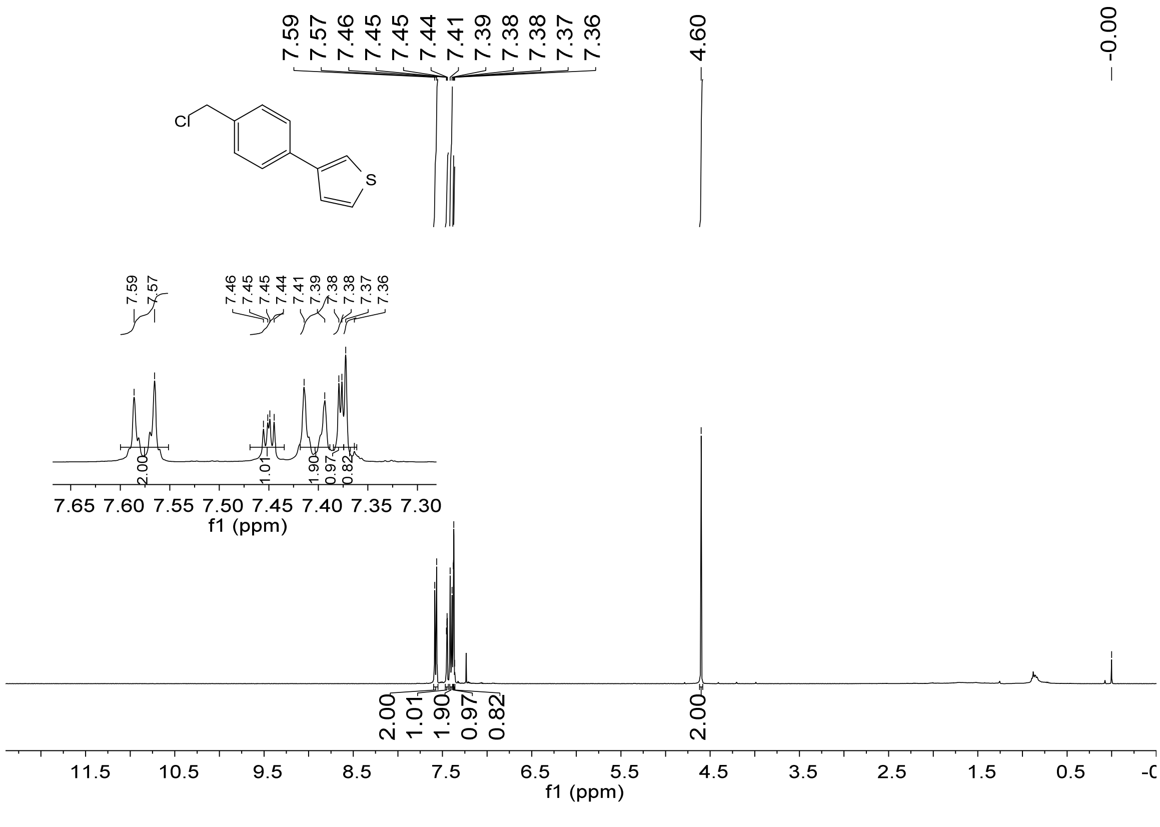

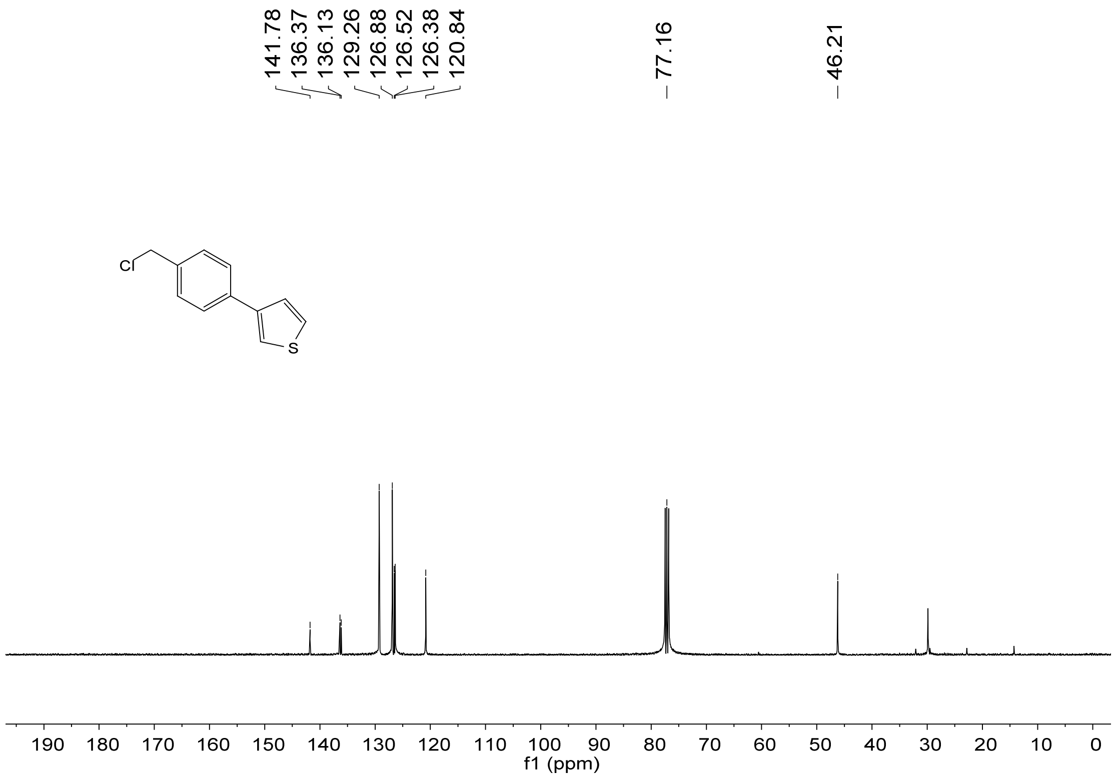

2-(4-(chloromethyl)phenyl)naphthalene[3o]

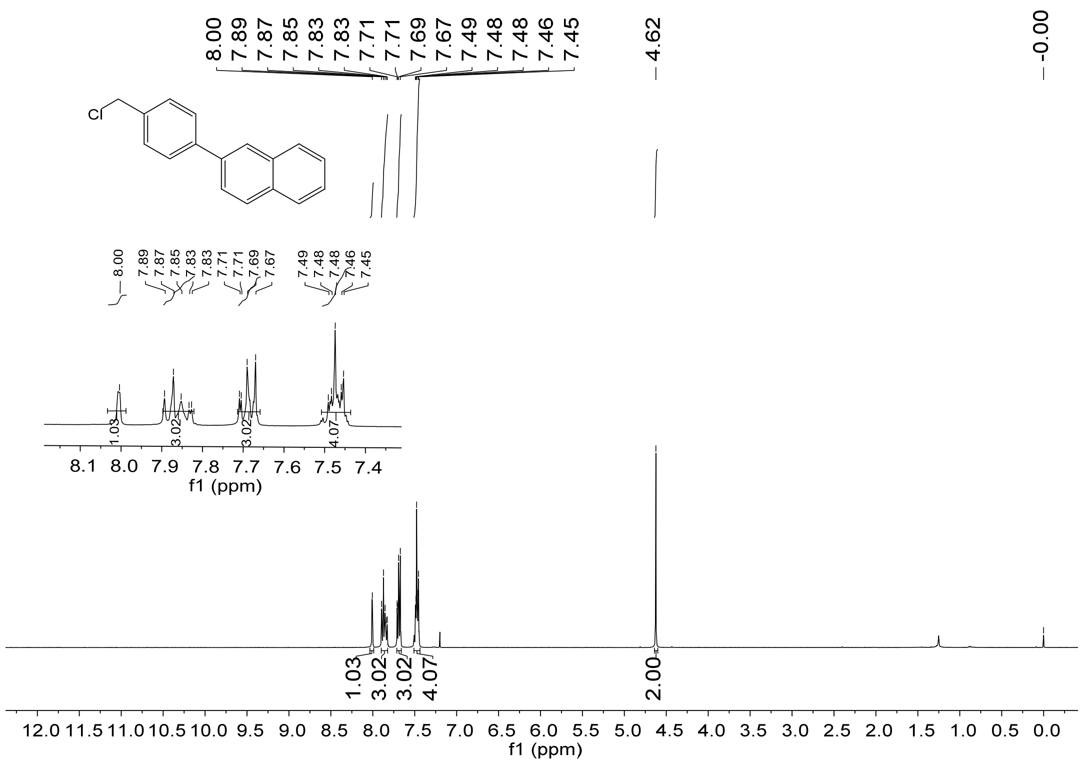

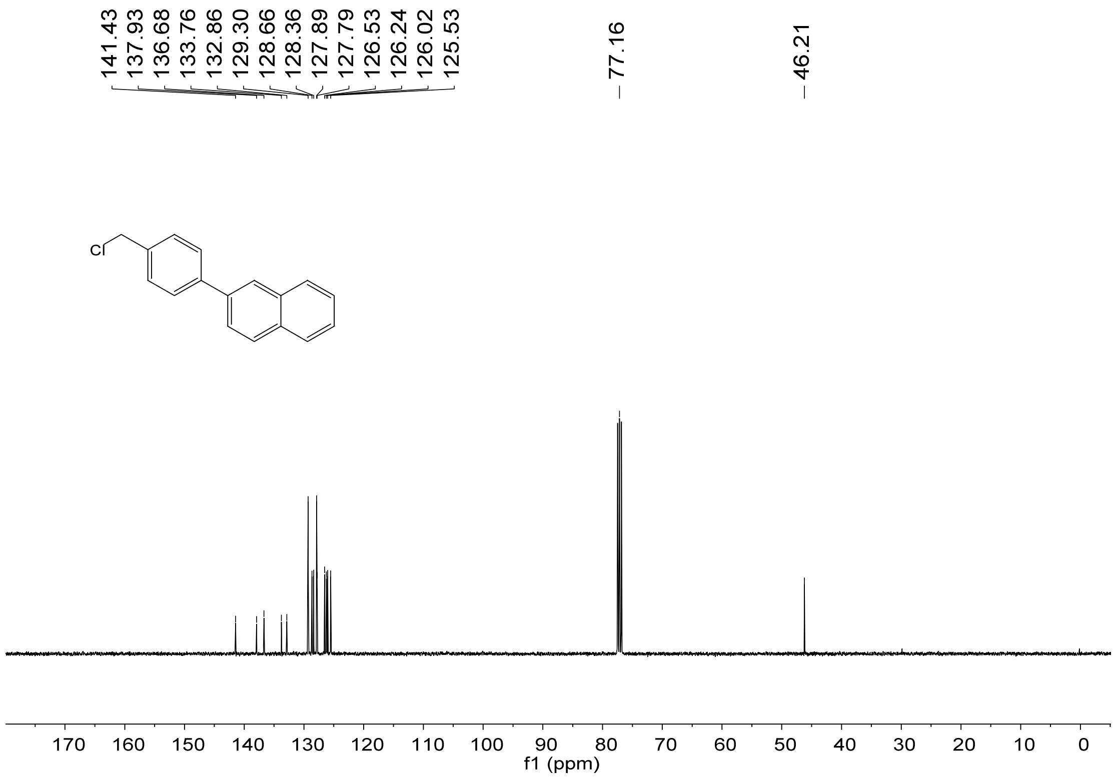

4-(chloromethyl)-4'-vinyl-1,1'-biphenyl[3p]

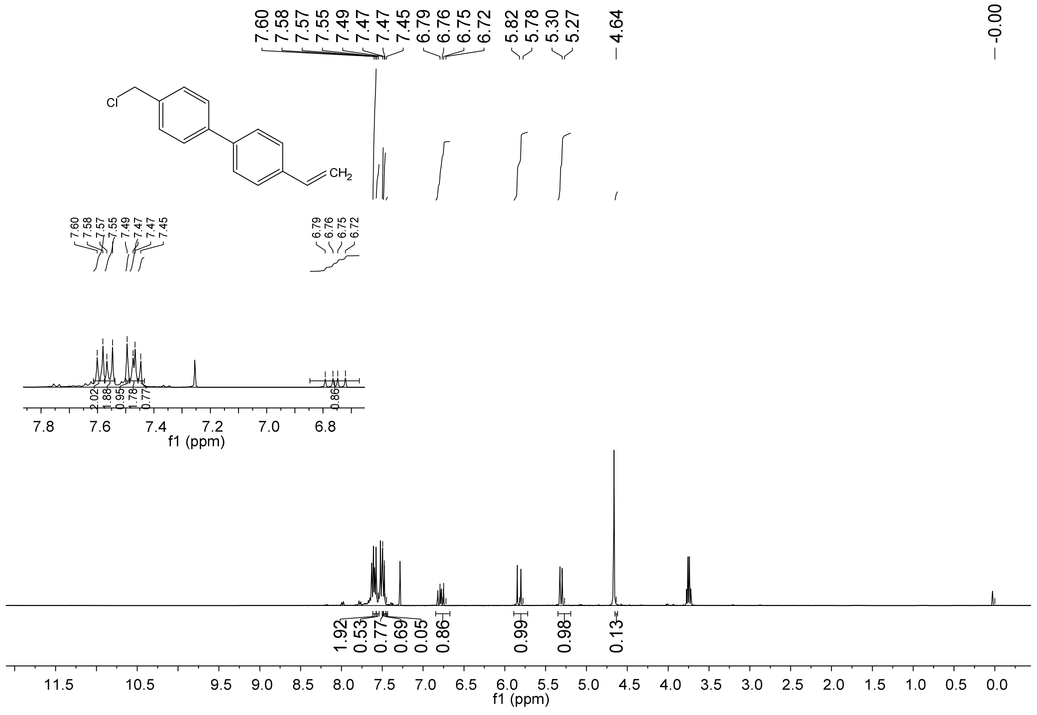

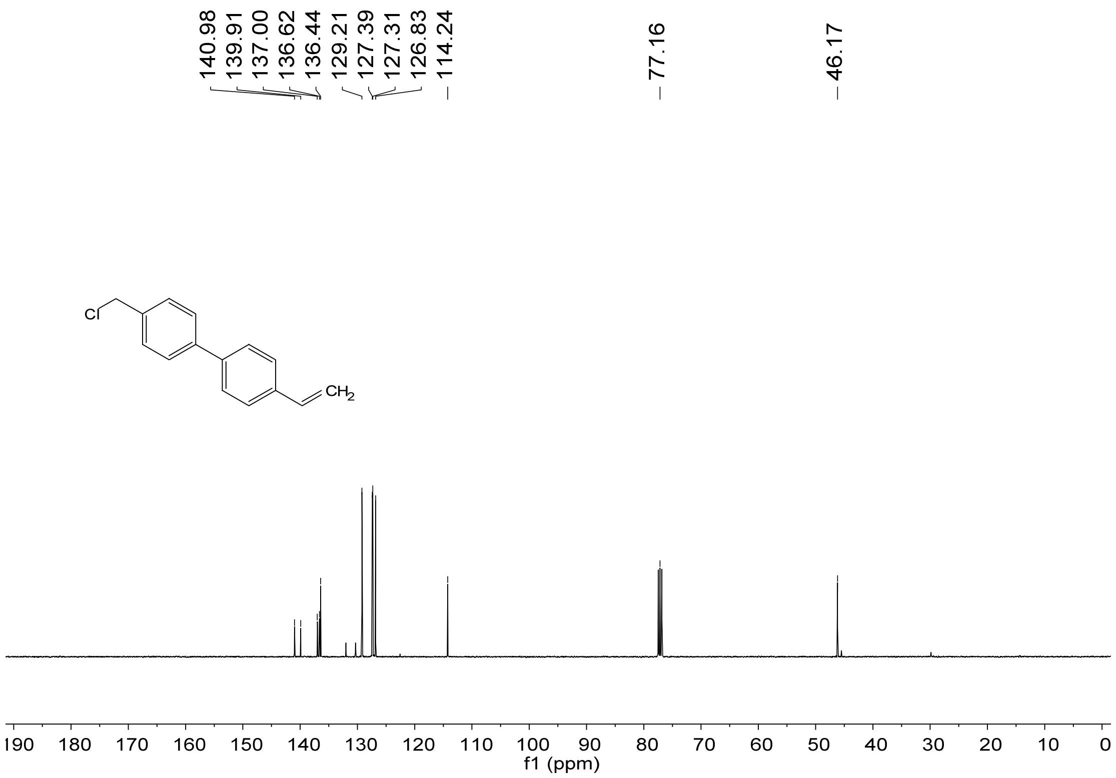
140.98
139.91
137.00
136.62
136.44
129.21
127.39
127.31
126.83
114.24
77.16
46.17
Cl
CH2
190 180 170 160 150 140 130 120 110 100 90 80 70 60 50 40 30 20 10 0
f1 (ppm)

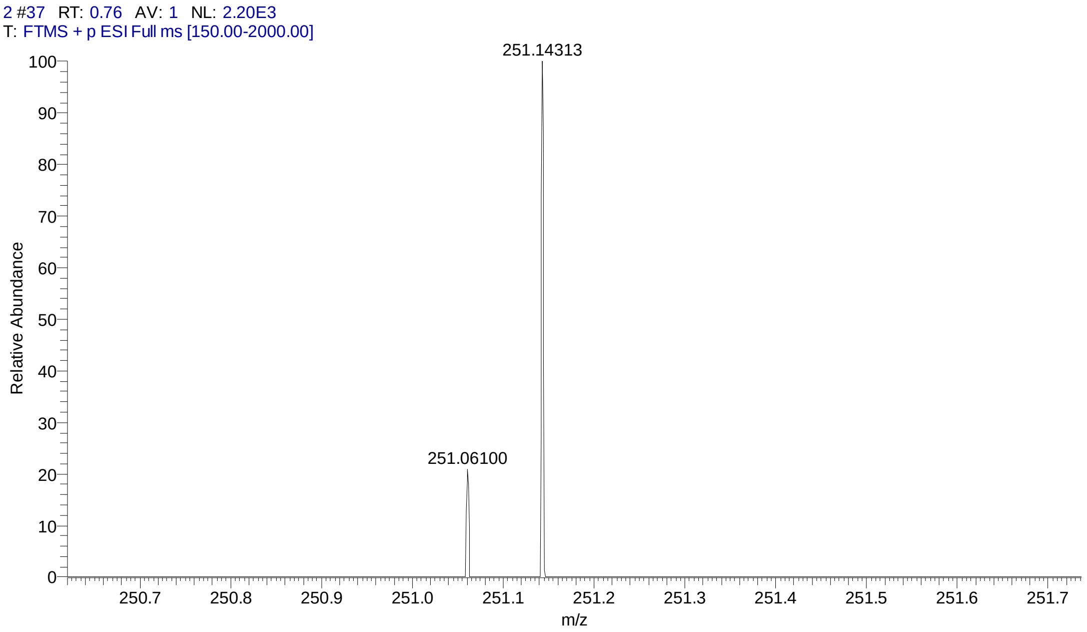
2 #37 RT: 0.76 AV: 1 NL: 2.20E3
T: FTMS + p ESI Full ms [150.00-2000.00]
251.14313
251.06100
Relative Abundance
m/z

3-(chloromethyl)-1,1'-biphenyl[4a]

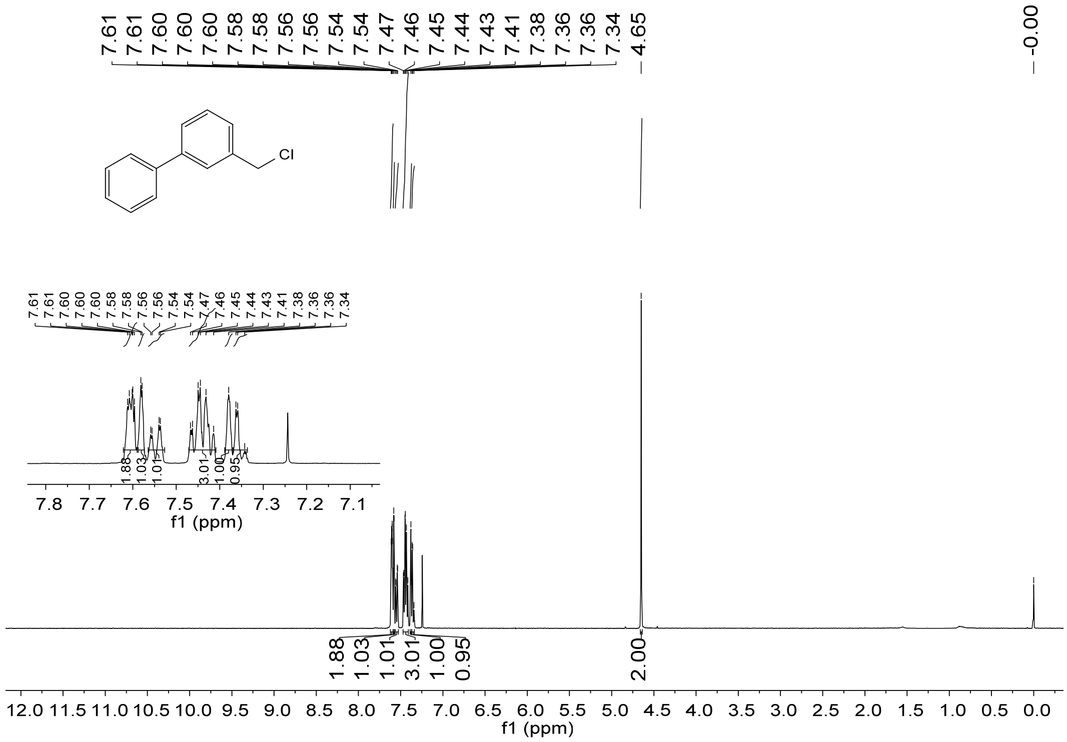

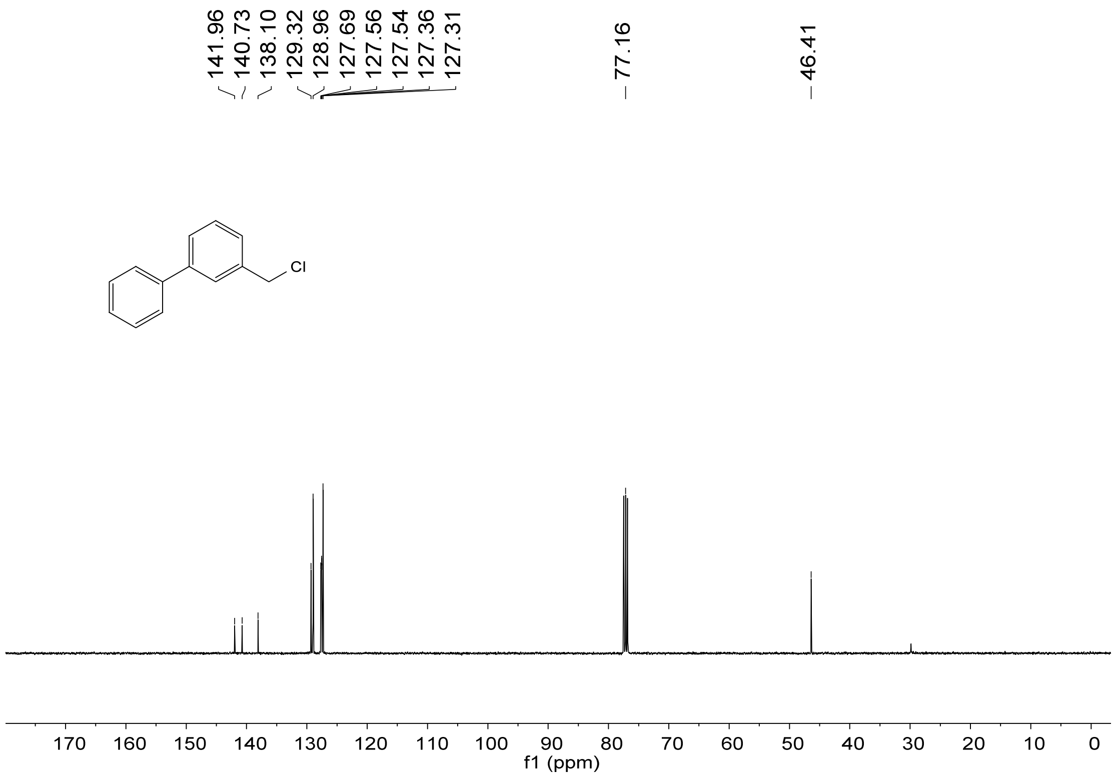

3-(chloromethyl)-4'-methyl-1,1'-biphenyl[4b]

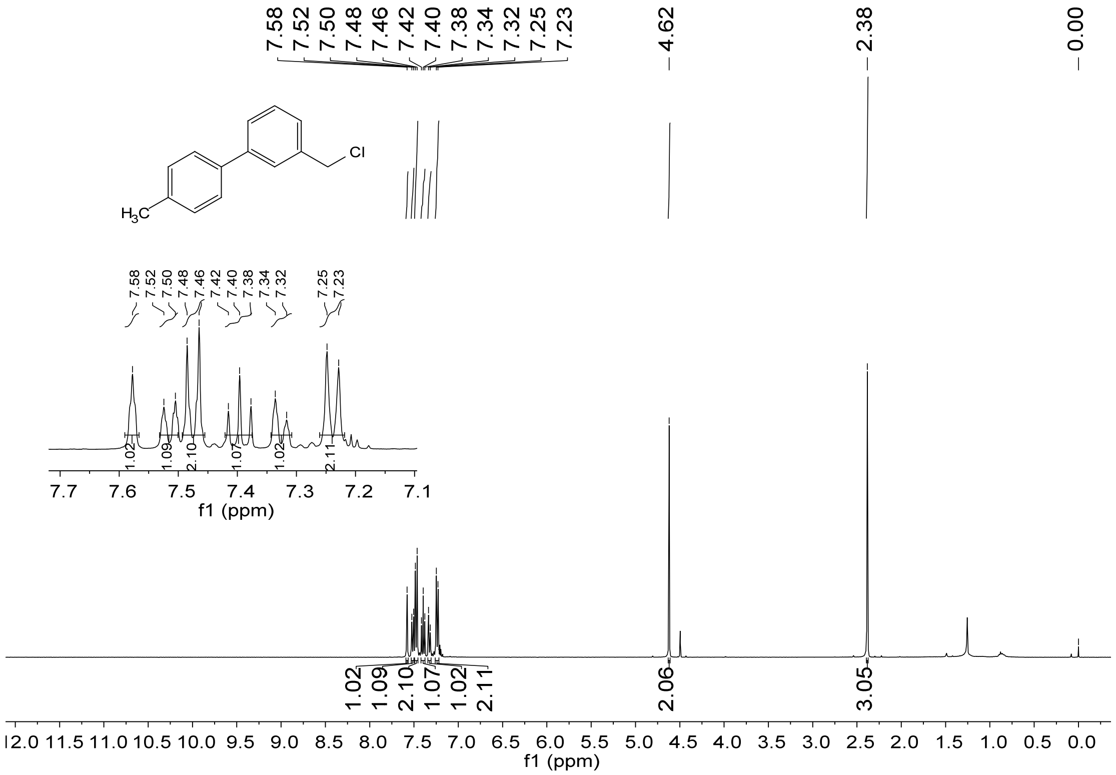

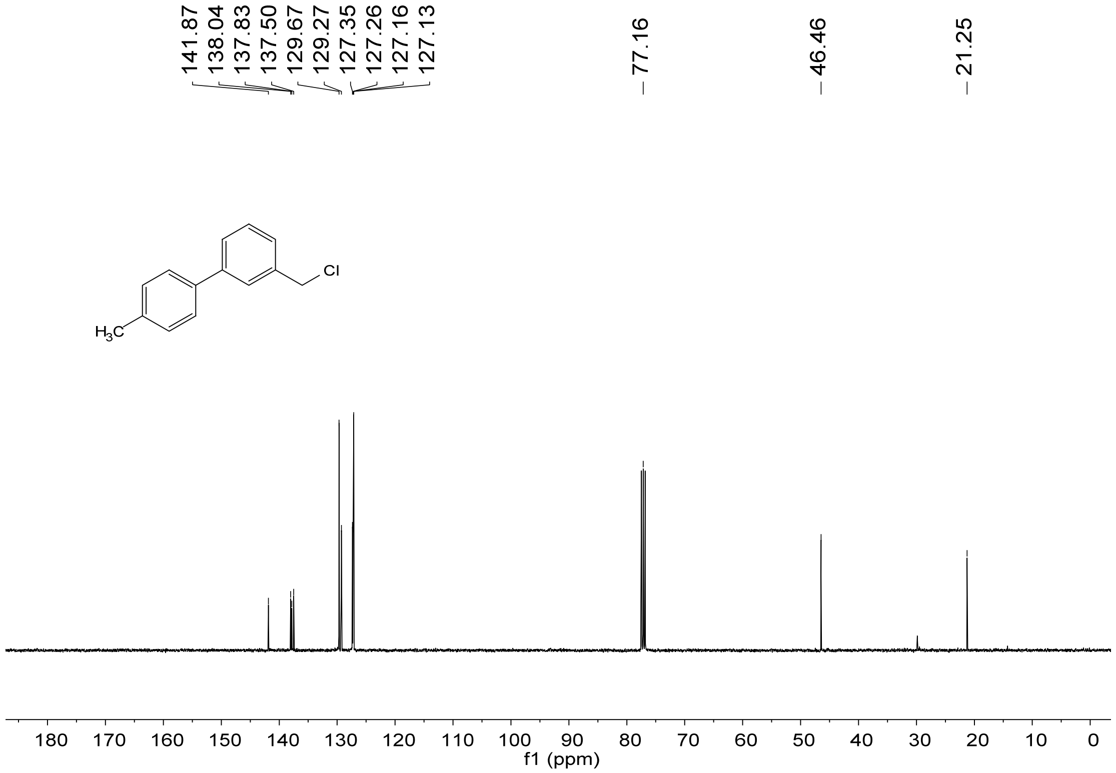

3-(chloromethyl)-4'-(trifluoromethyl)-1,1'-biphenyl[4c]

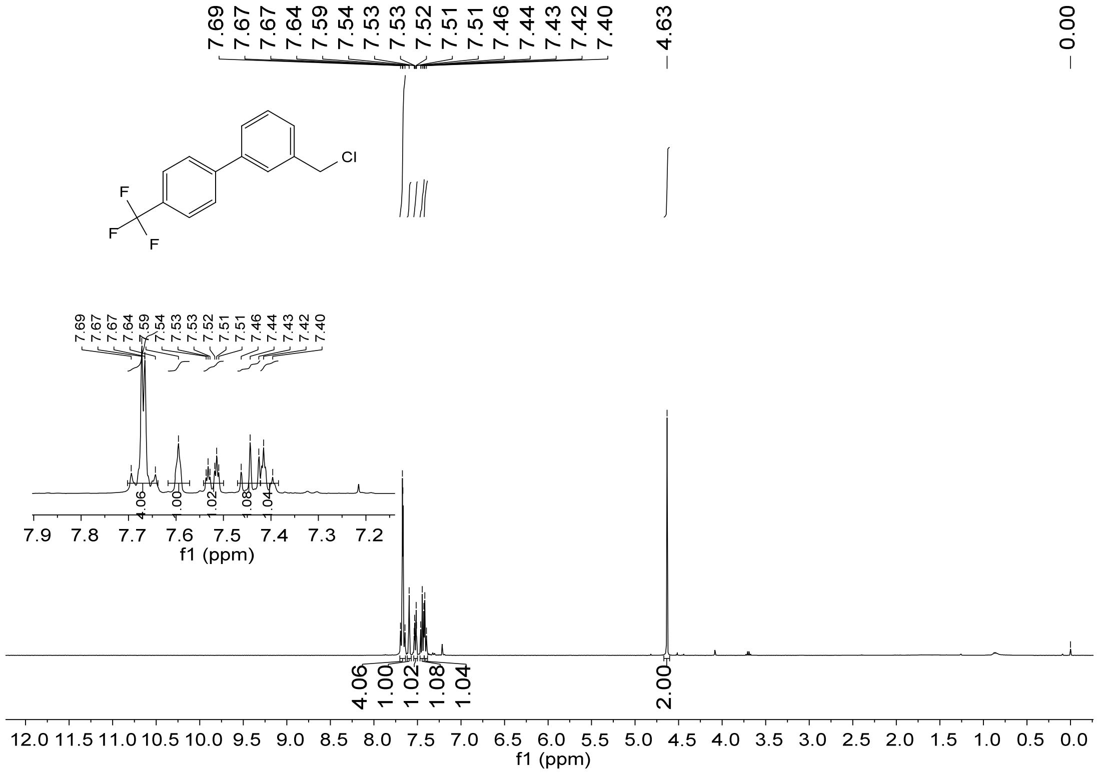

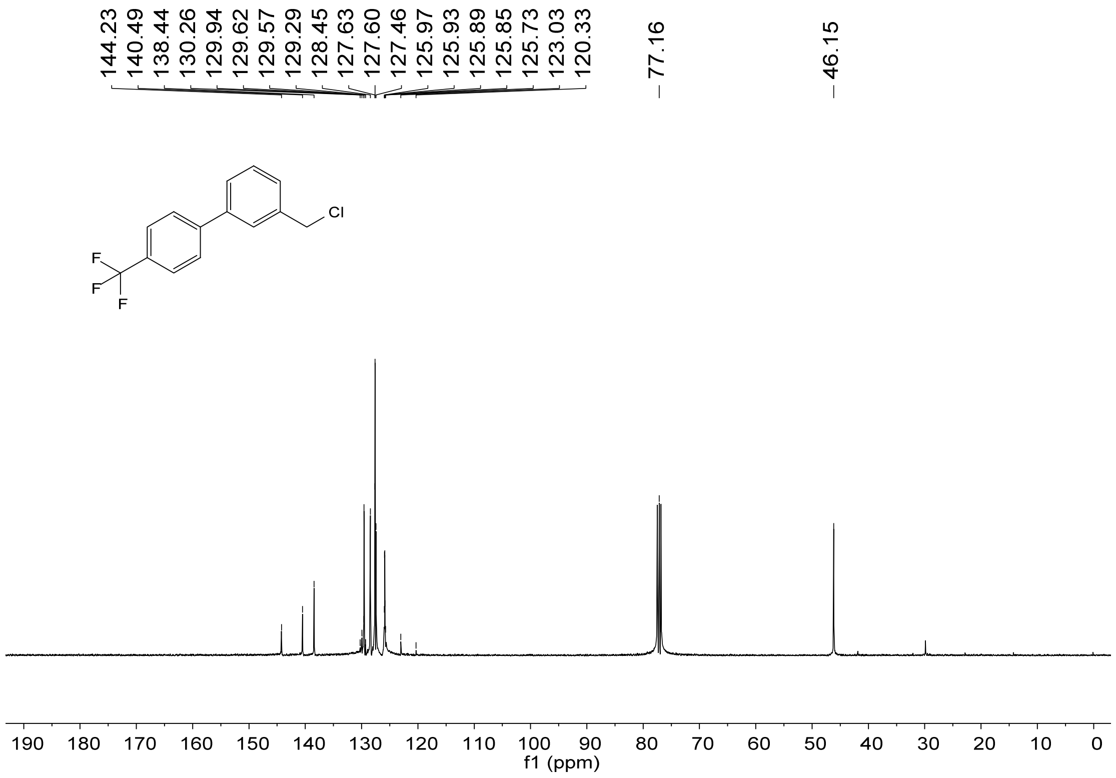

3-(chloromethyl)-3'-methyl-1,1'-biphenyl[4d]

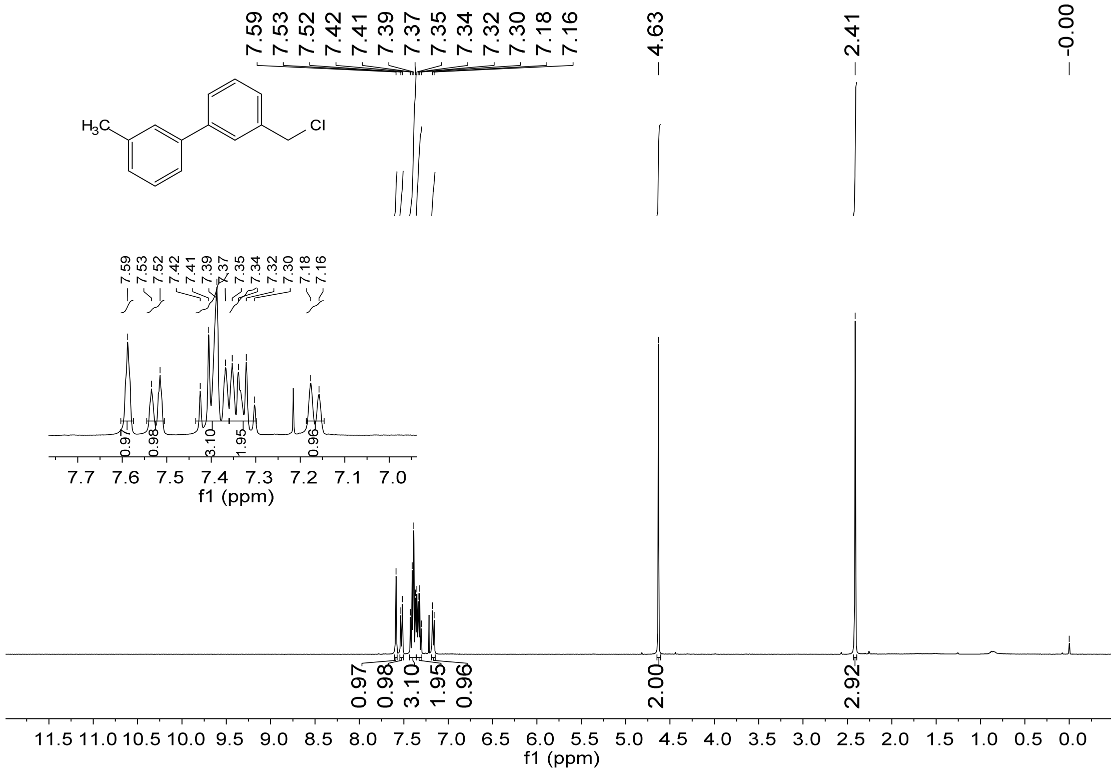

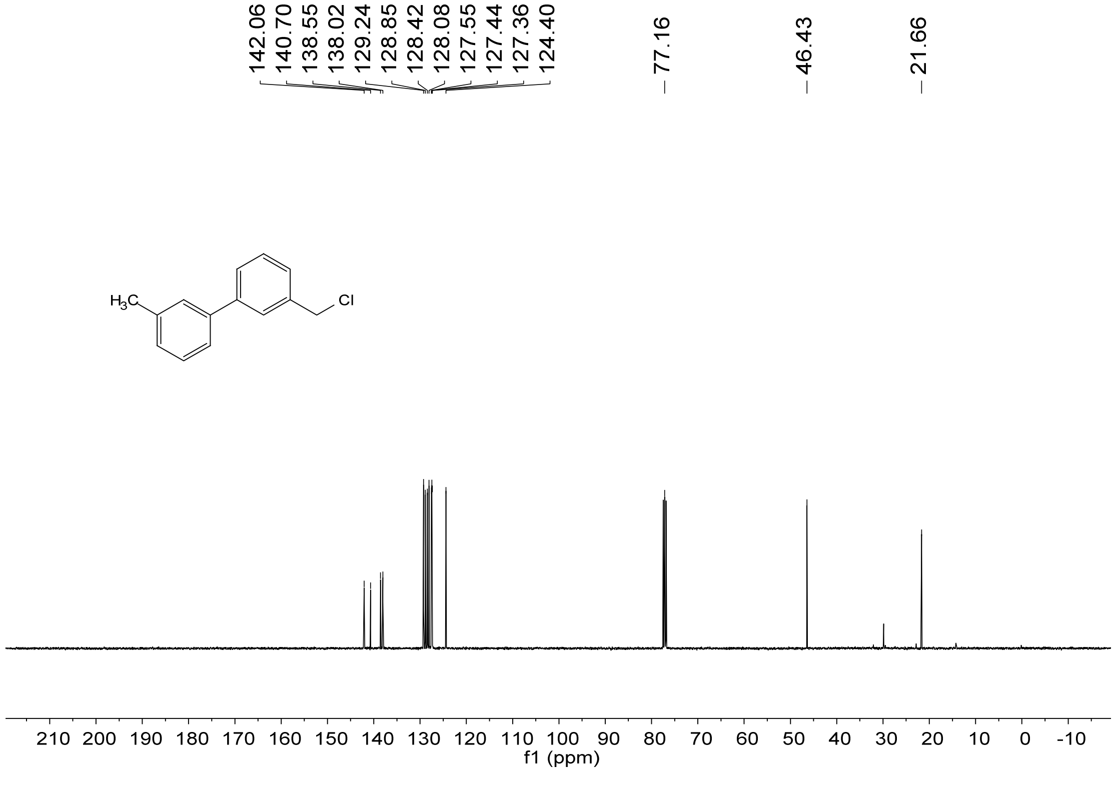

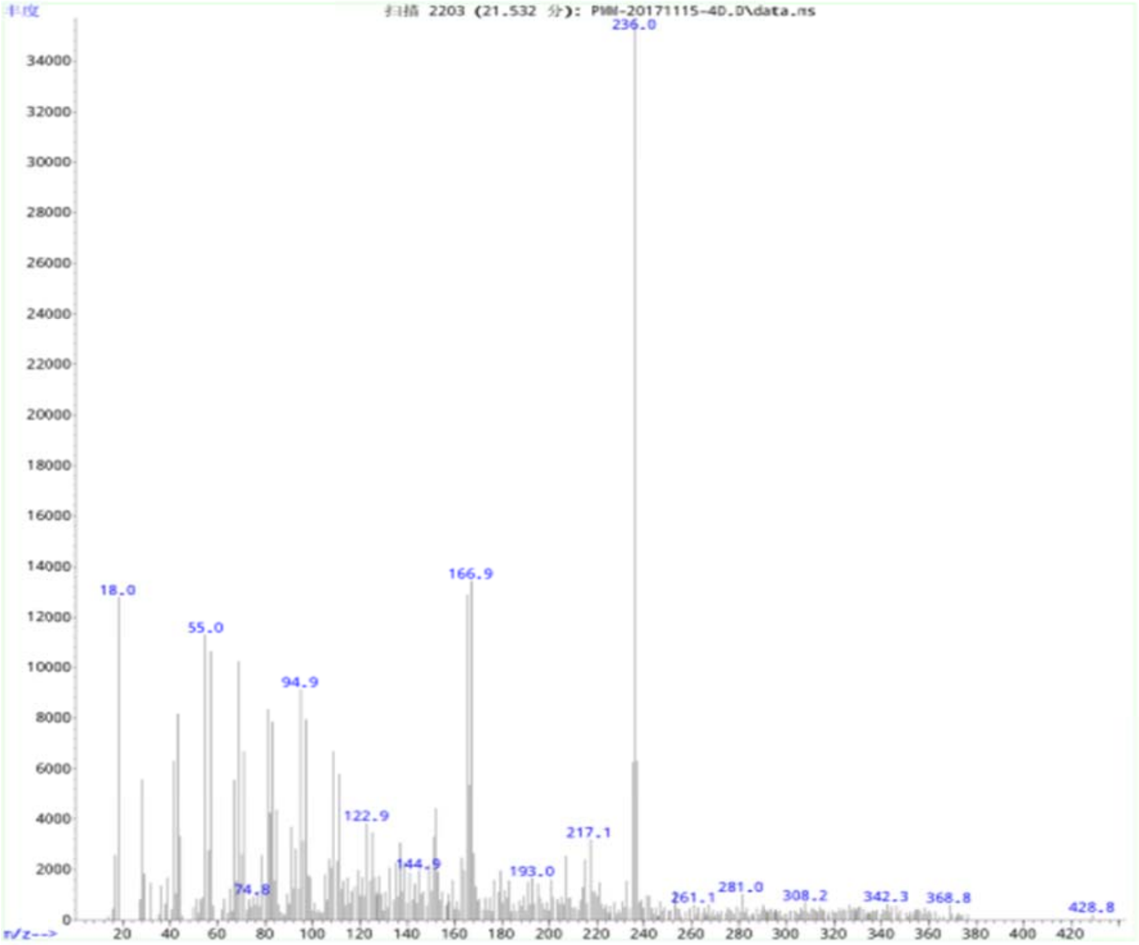

3-chloro-3'-(chloromethyl)-1,1'-biphenyl[4e]

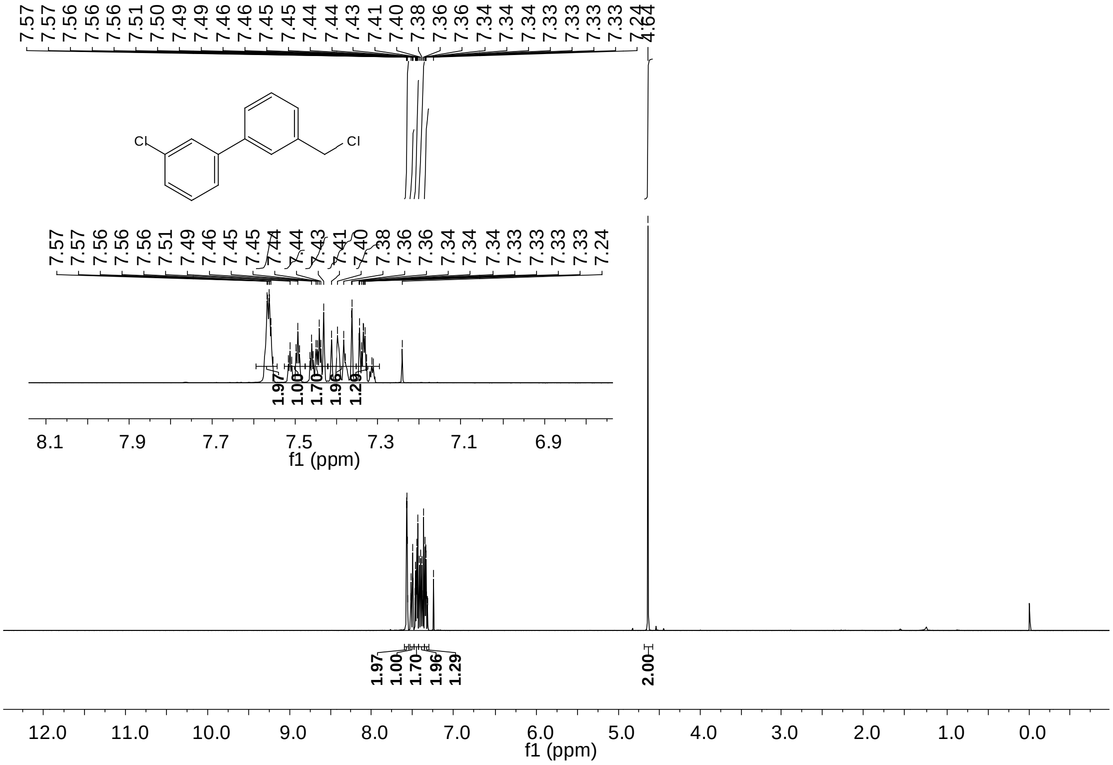

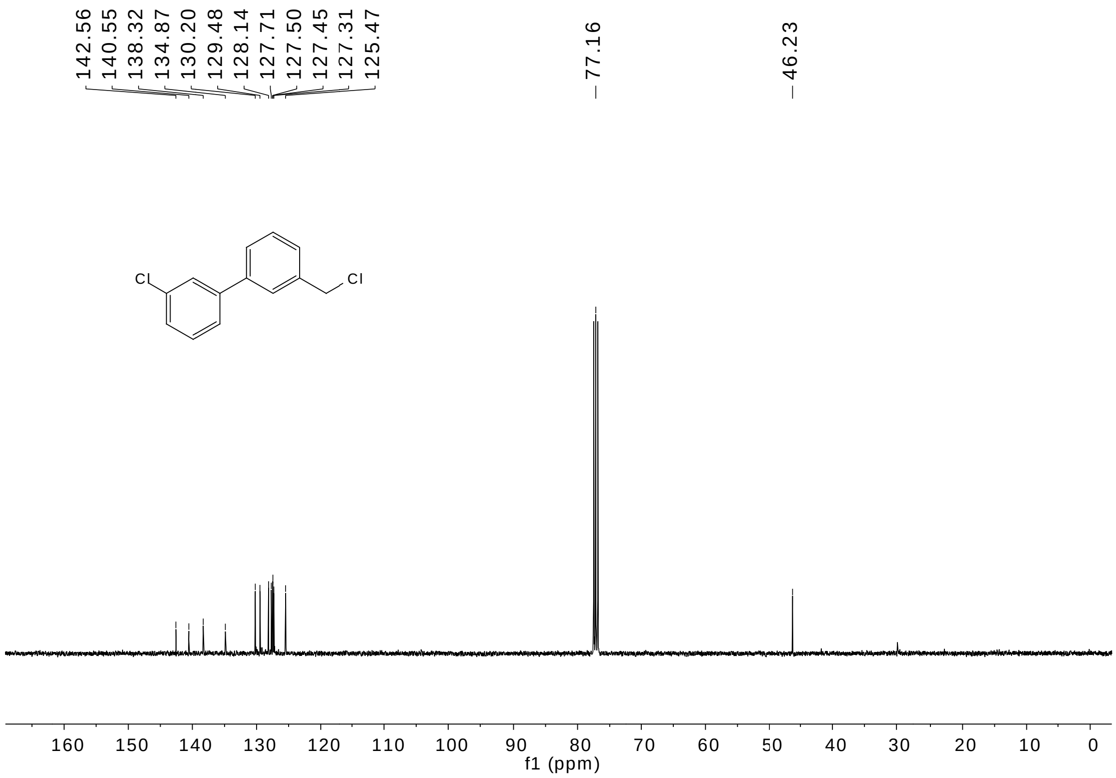

Elemental Composition Report **Page 1**

Single Mass Analysis (displaying only valid results)
Tolerance = 10.0 PPM / DBE: min = -1.5, max = 50.0
Element prediction: Off

Monoisotopic Mass, Odd and Even Electron Ions
20 formula(e) evaluated with 1 results within limits (up to 50 best isotopic matches for each mass)
Elements Used:
C: 0-15 H: 0-15 F: 0-3 Cl: 0-3
default file
HR-4 1857 (12.464) Cm (1854:1857-(1844:1852+1875:1886))

TOF MS EI+
9.12e+002

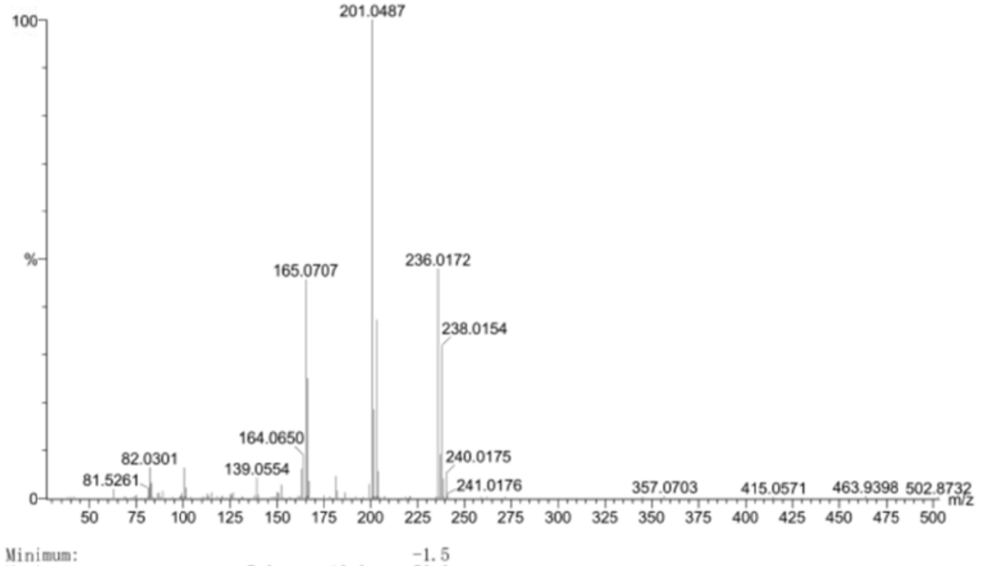

Mass	Calc. Mass	mDa	PPM	DBE	i-FIT	Formula
Minimum:				-1.5		
Maximum:		5.0	10.0	50.0		
236.0172	236.0160	1.2	5.1	8.0	2.2	C13 H10 Cl2

3'-(chloromethyl)-2-methyl-1,1'-biphenyl[4f]

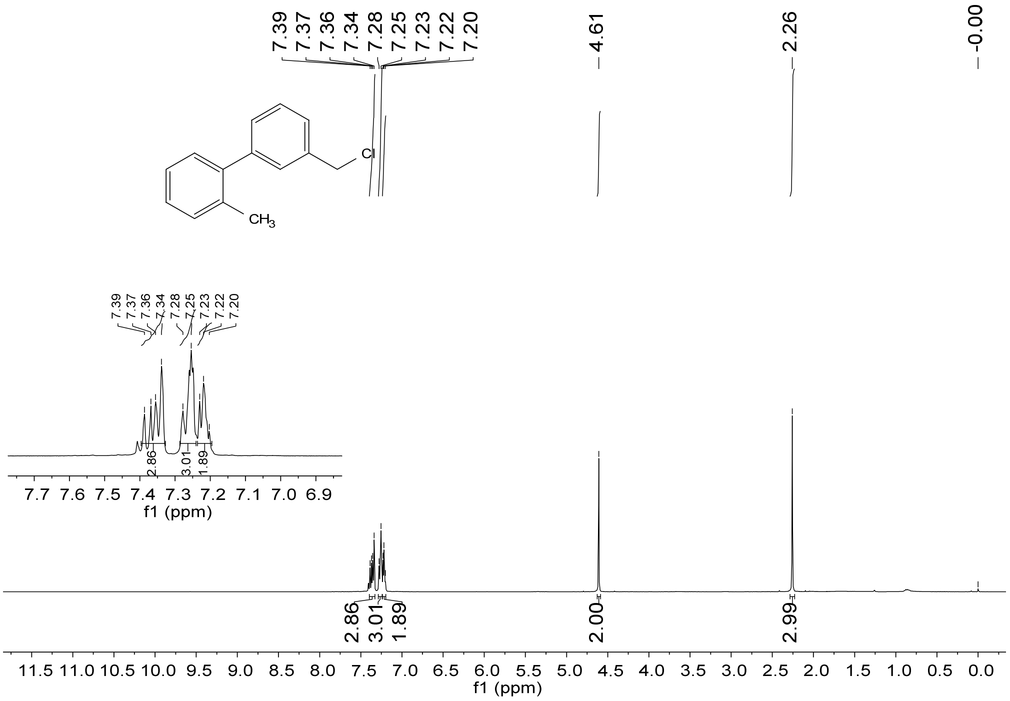

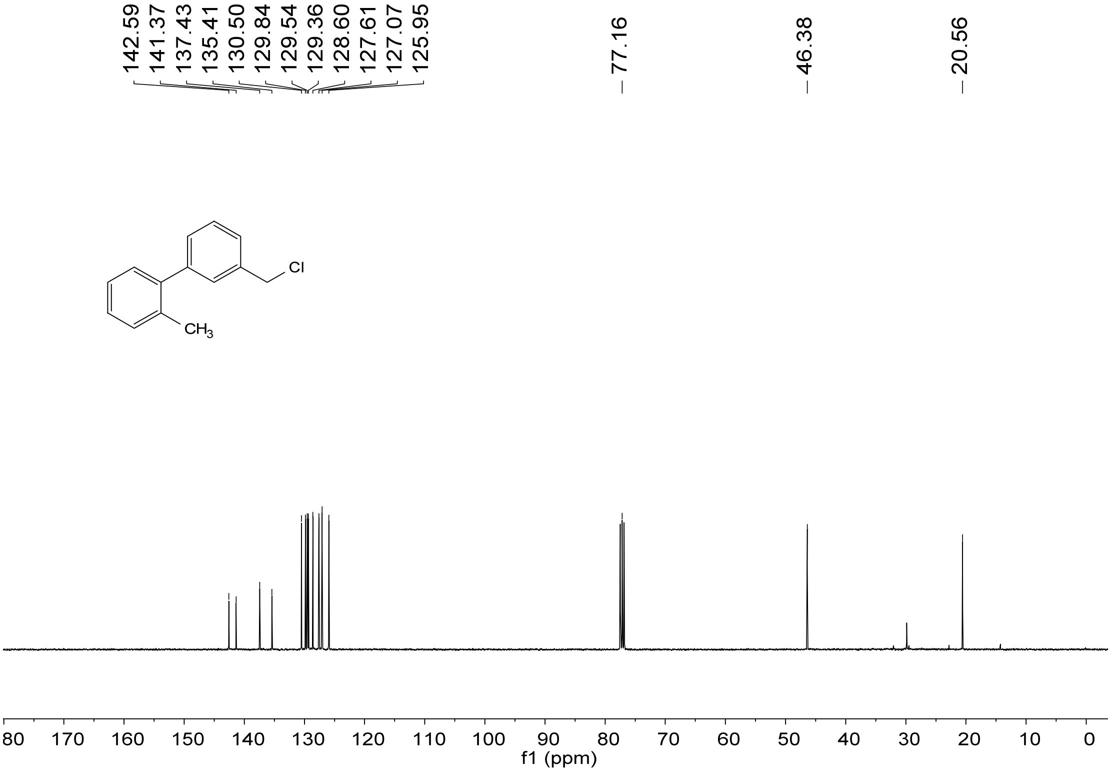

3-(3-(chloromethyl)phenyl)thiophene[4g]

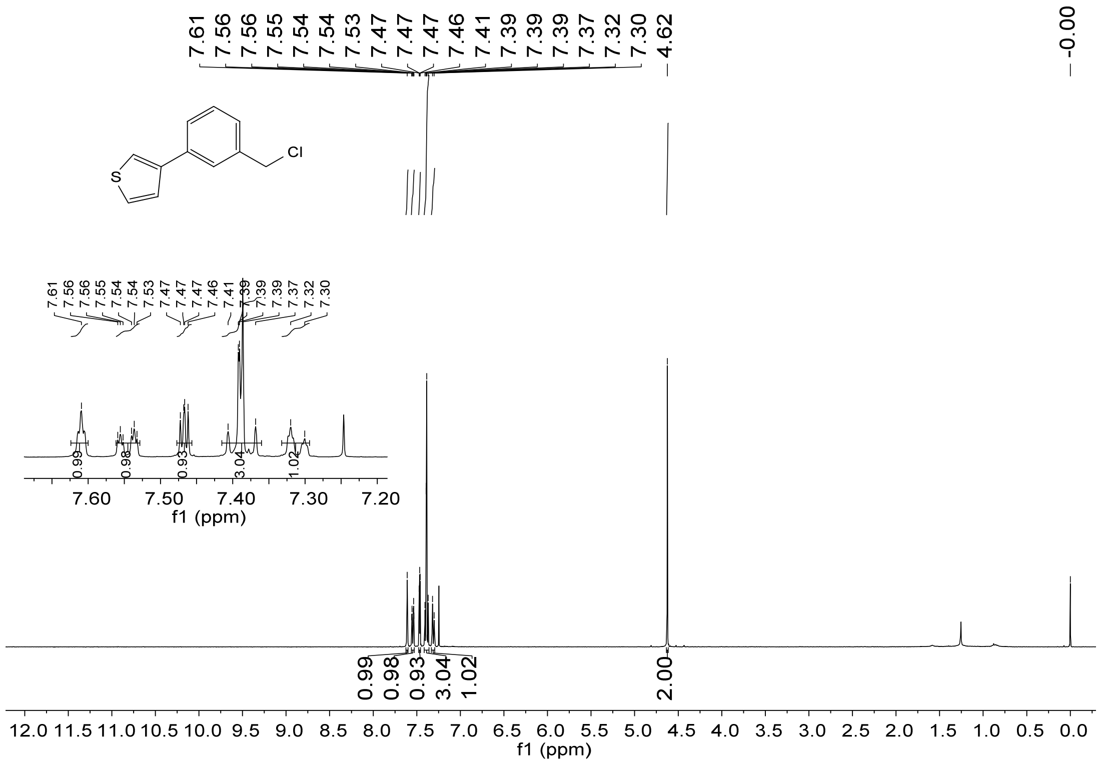

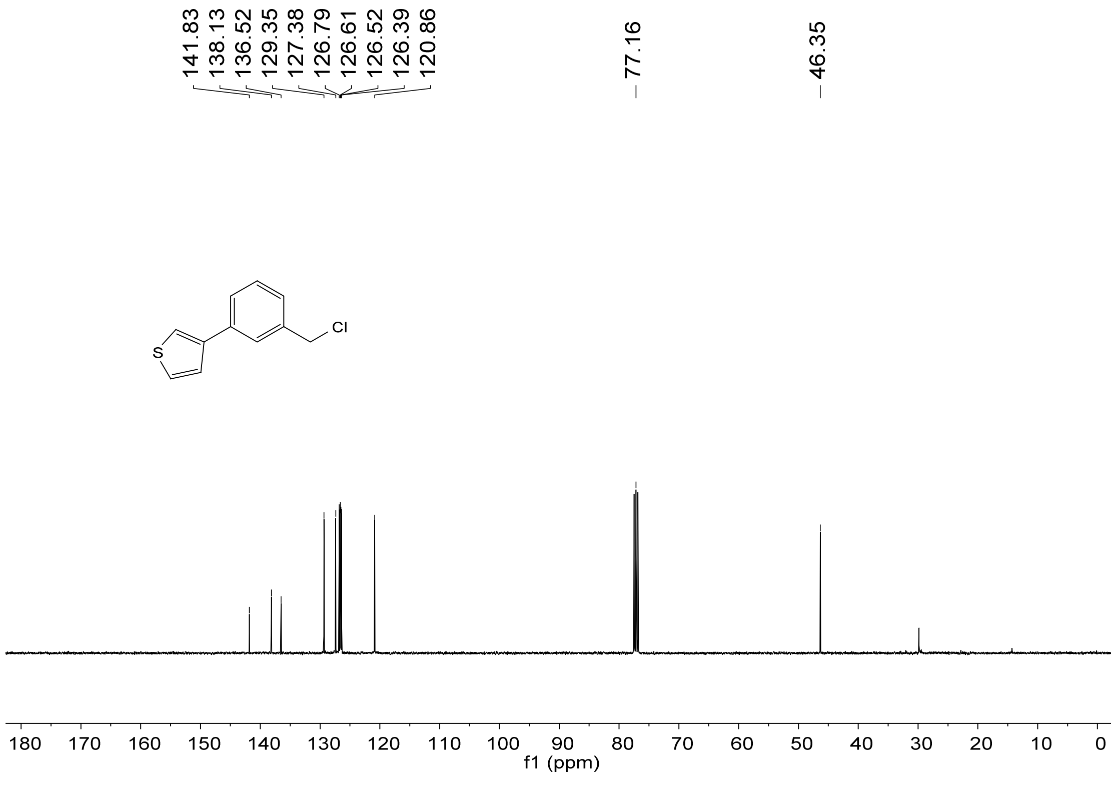

2-(3-(chloromethyl)phenyl)naphthalene[4h]

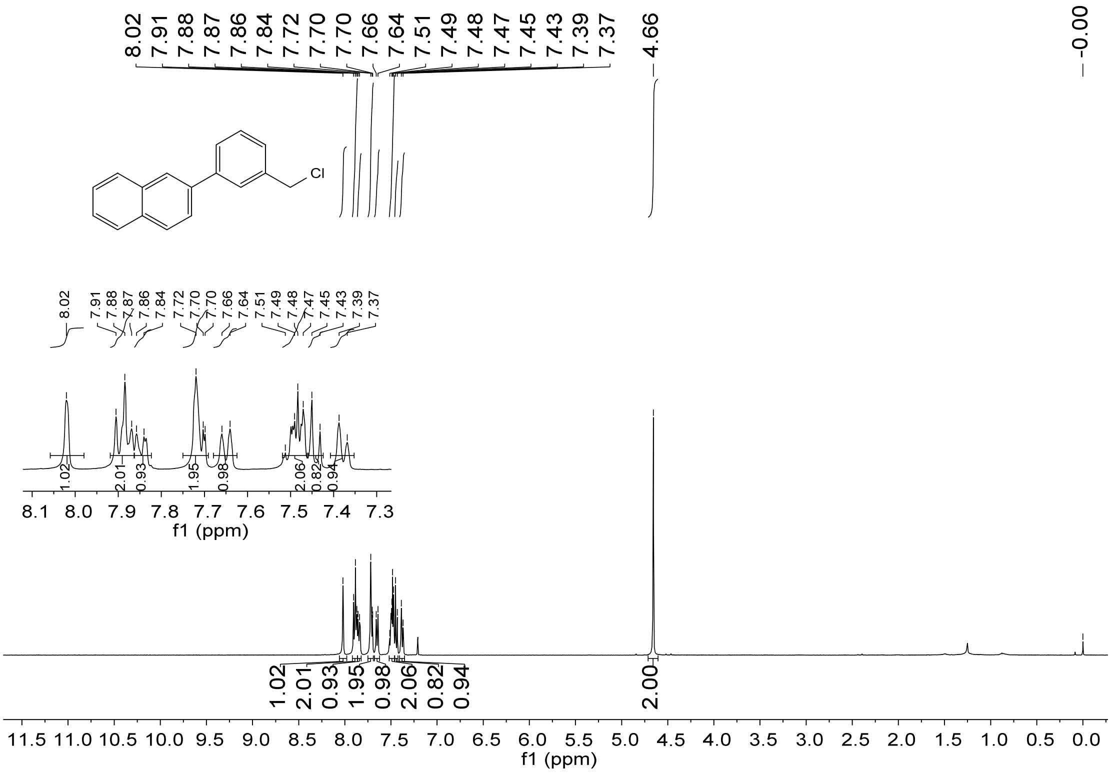

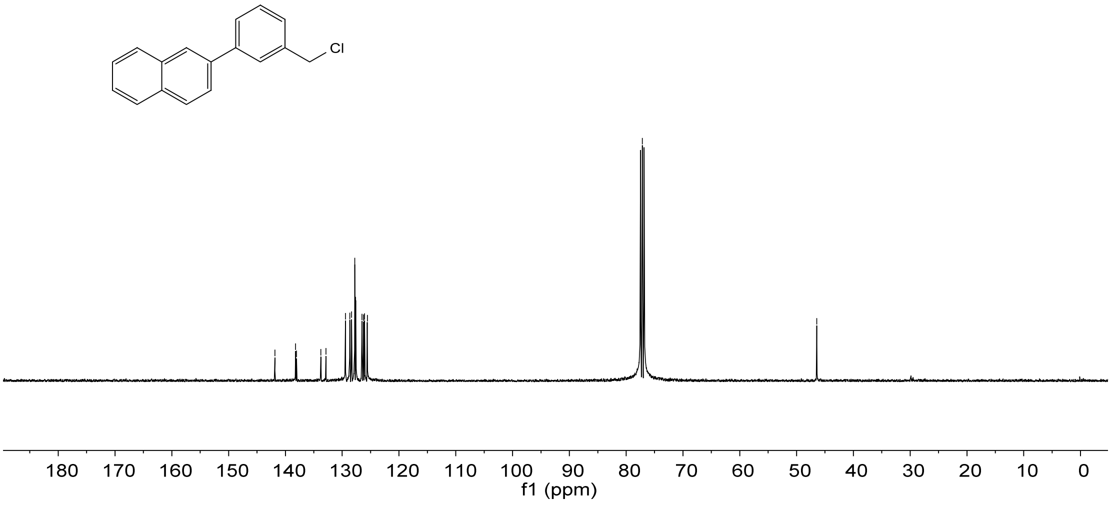

Elemental Composition Report **Page 1**

Single Mass Analysis (displaying only valid results)
Tolerance = 10.0 PPM / DBE: min = -1.5, max = 50.0
Element prediction: Off

Monoisotopic Mass, Odd and Even Electron Ions
49 formula(e) evaluated with 1 results within limits (up to 50 best isotopic matches for each mass)
Elements Used:
C: 0-30 H: 0-30 F: 0-4 Cl: 0-3

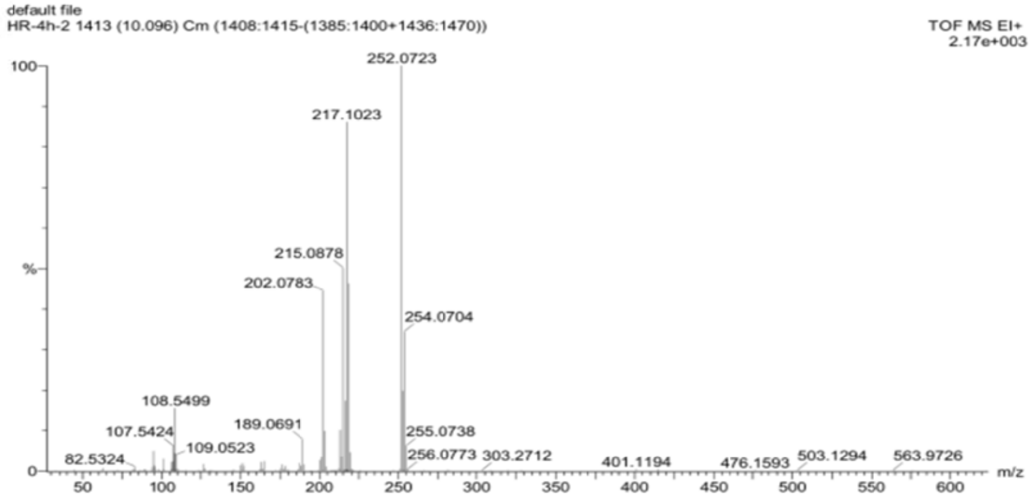

Minimum:				-1.5		
Maximum:		5.0	10.0	50.0		
Mass	Calc. Mass	mDa	PPM	DBE	i-FIT	Formula
252.0723	252.0706	1.7	6.7	11.0	0.8	C17 H13 Cl

2-(chloromethyl)-1,1'-biphenyl[5a]

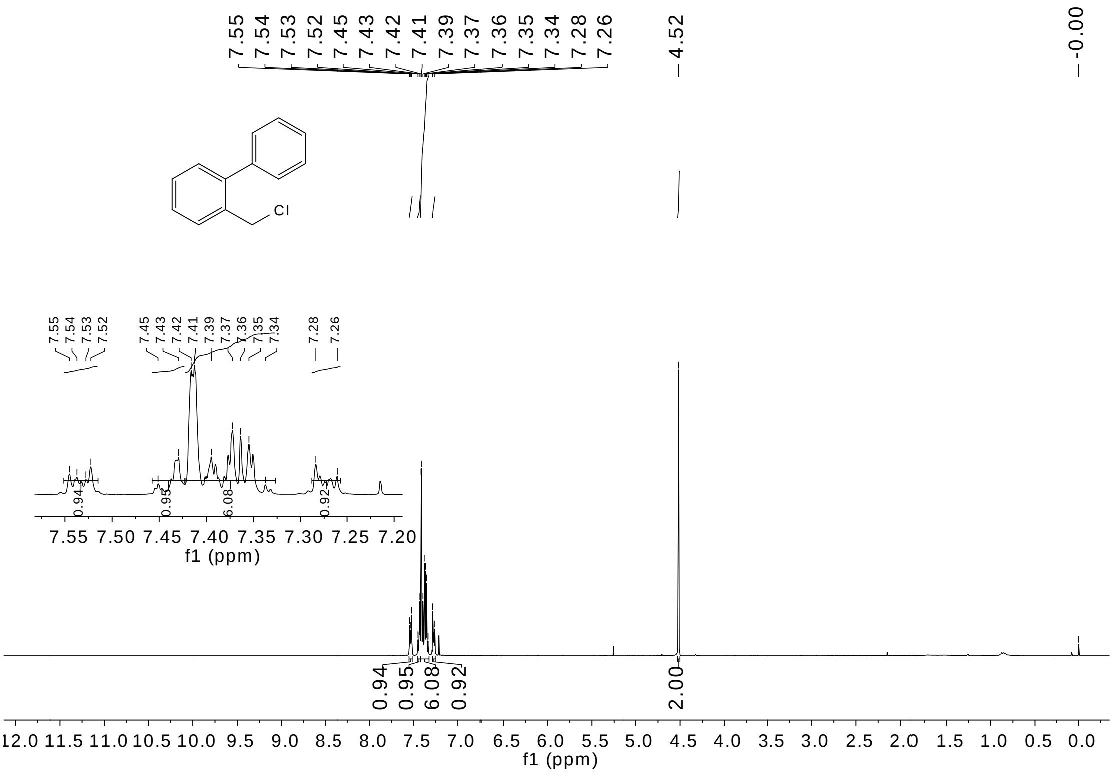

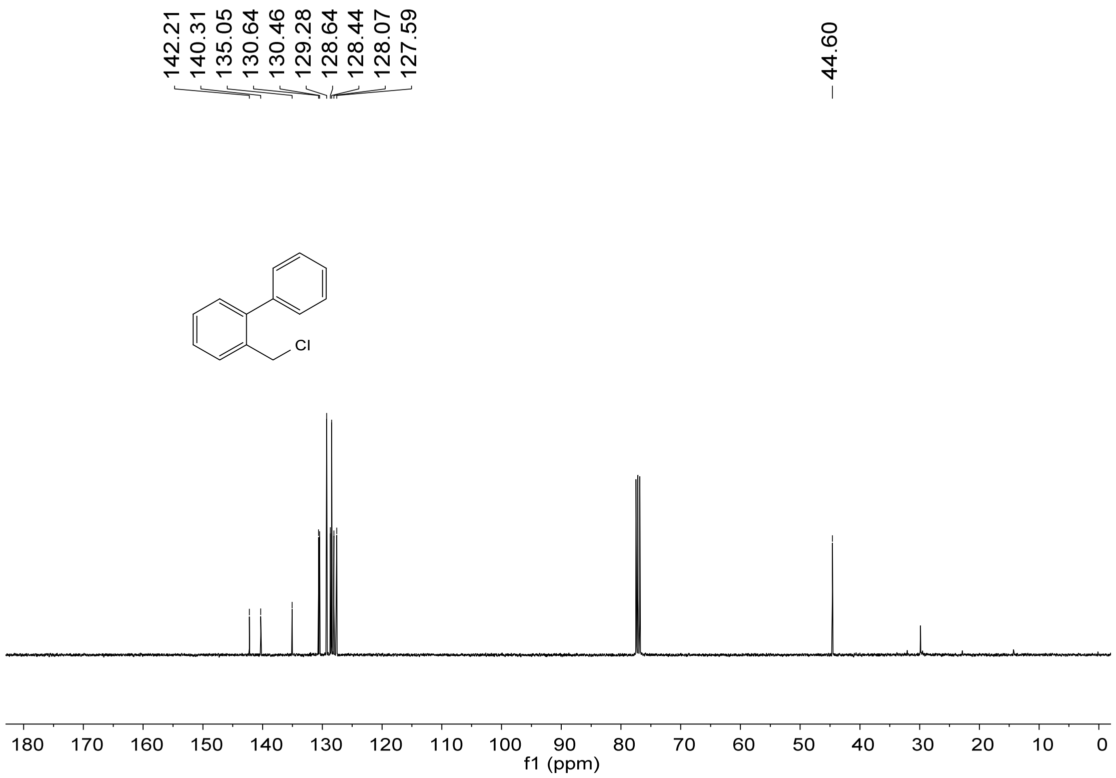

2-(chloromethyl)-4'-methyl-1,1'-biphenyl[5b]

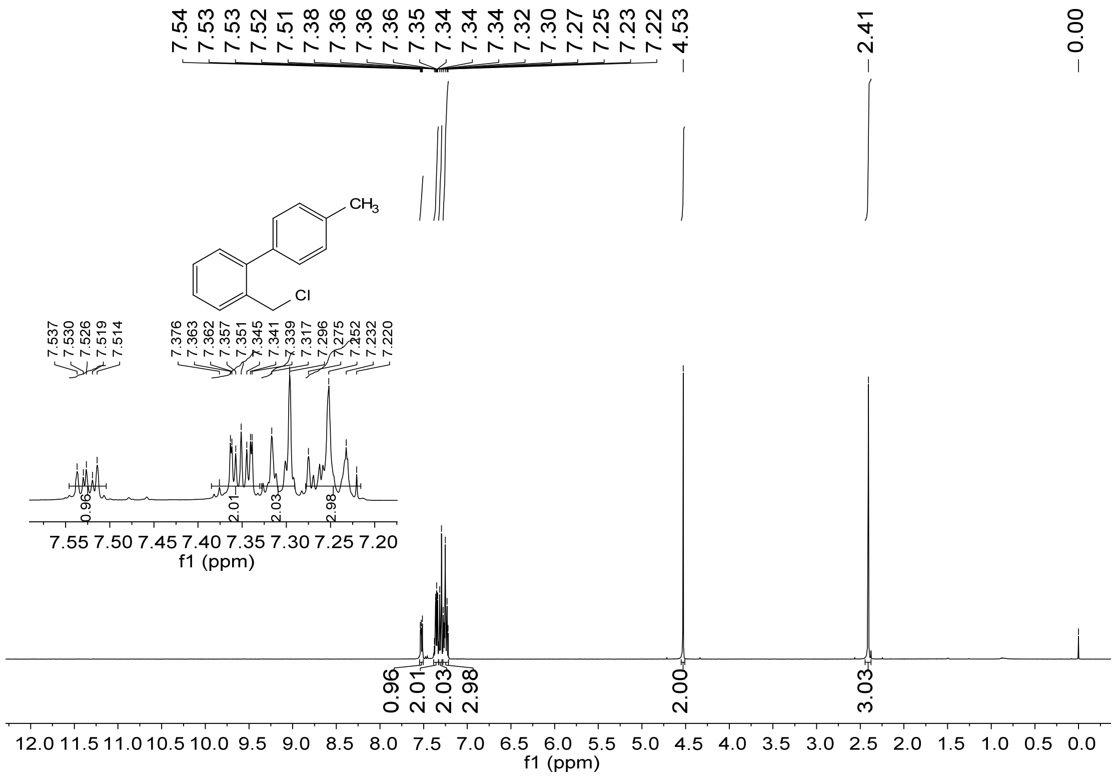

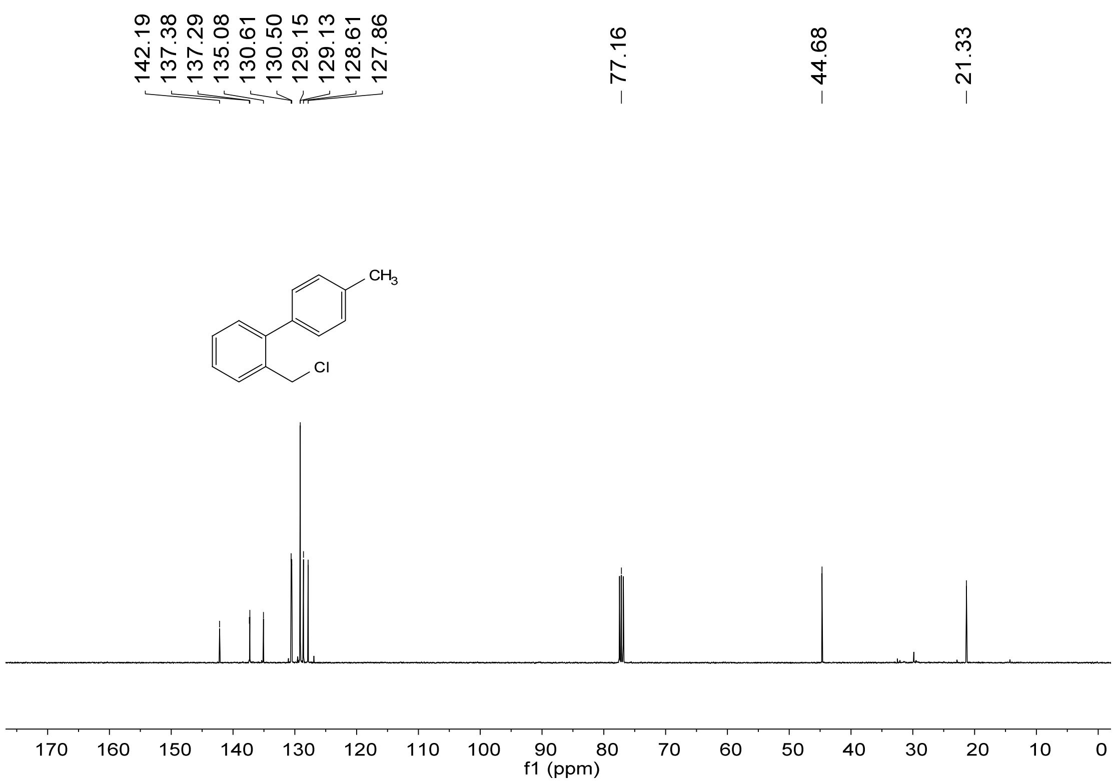

2-(chloromethyl)-4'-(trifluoromethyl)-1,1'-biphenyl[5c]

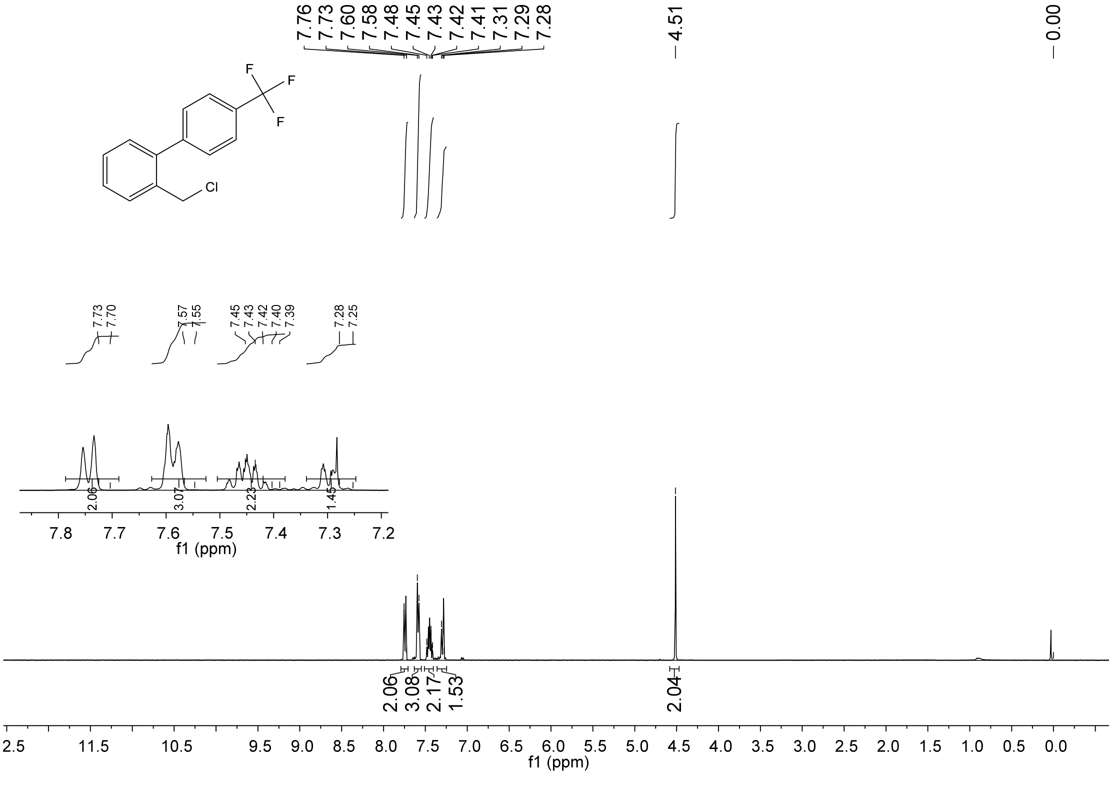

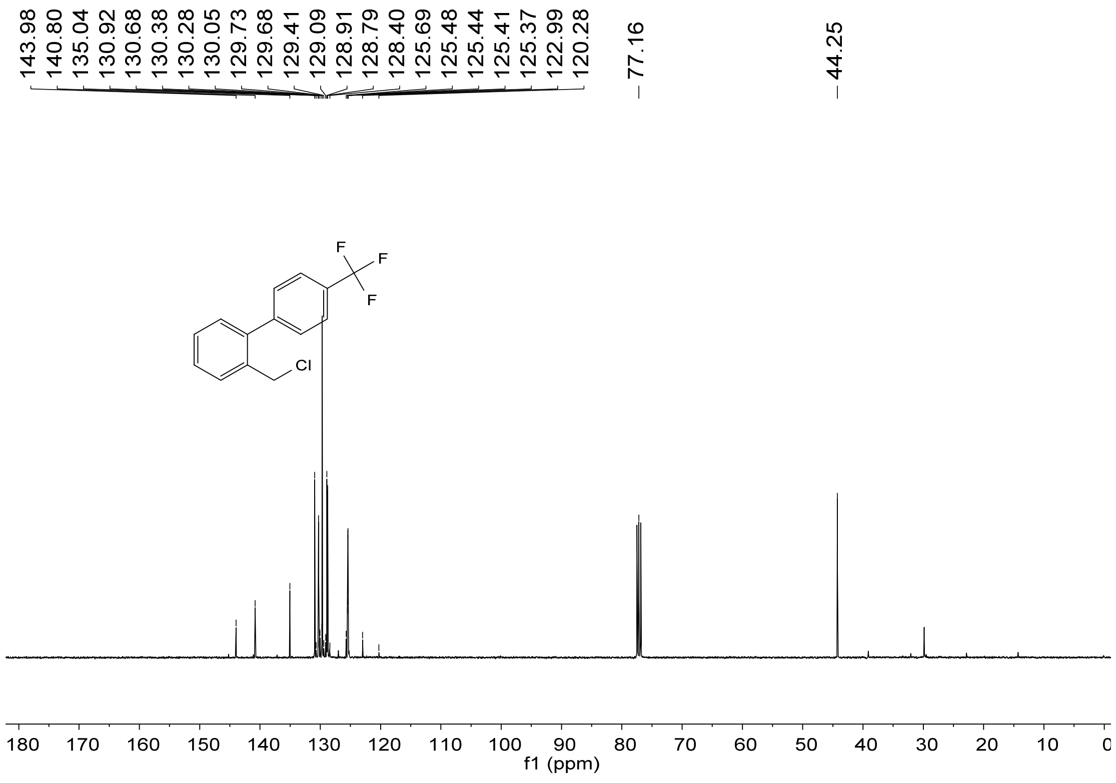

2-(chloromethyl)-3'-methyl-1,1'-biphenyl[5d]

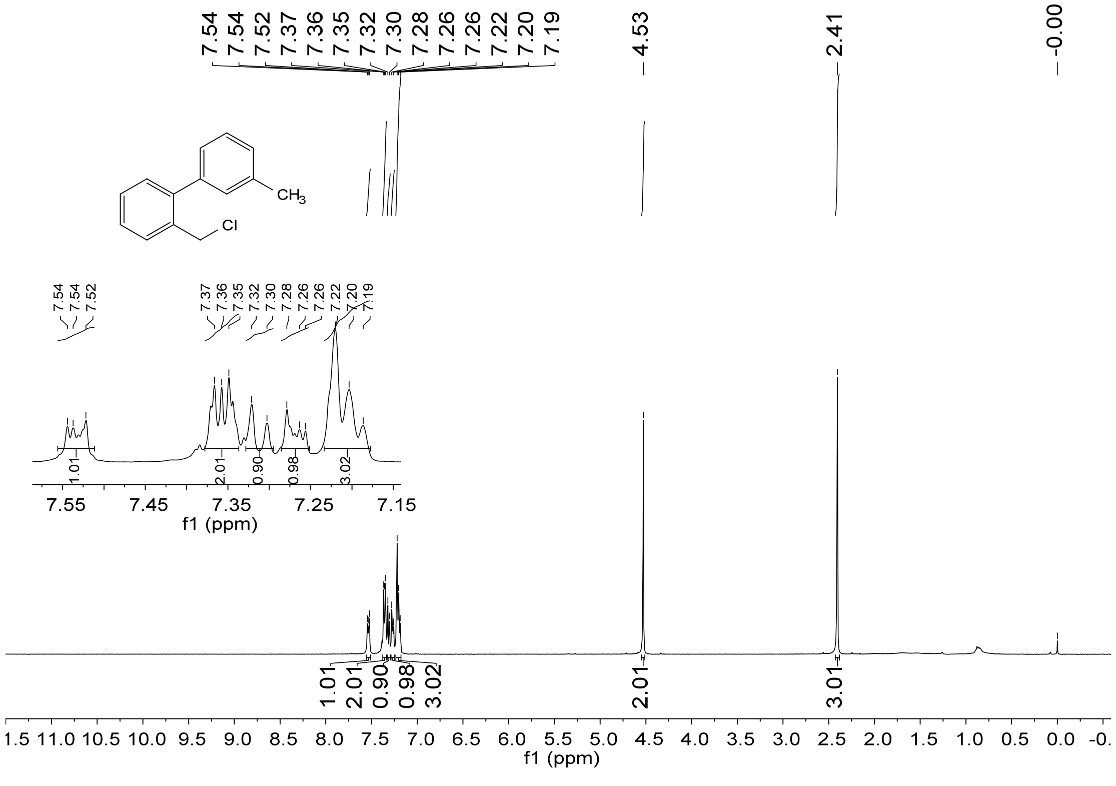

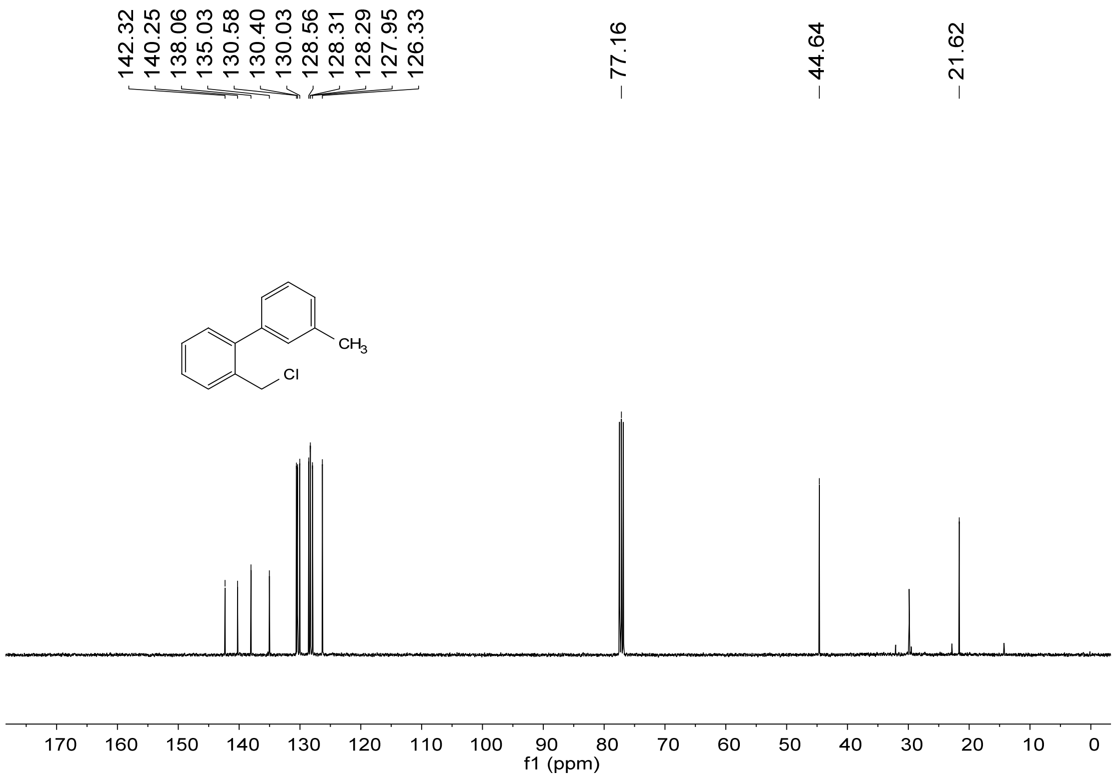

3'-chloro-2-(chloromethyl)-1,1'-biphenyl[5e]

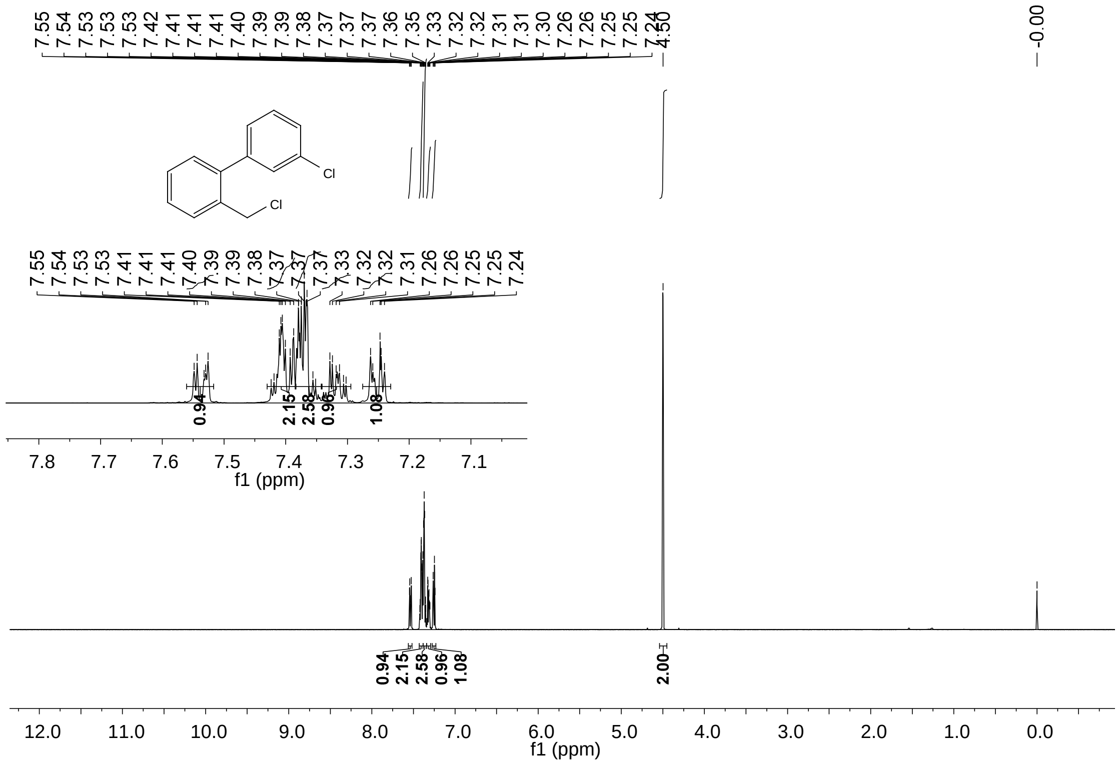

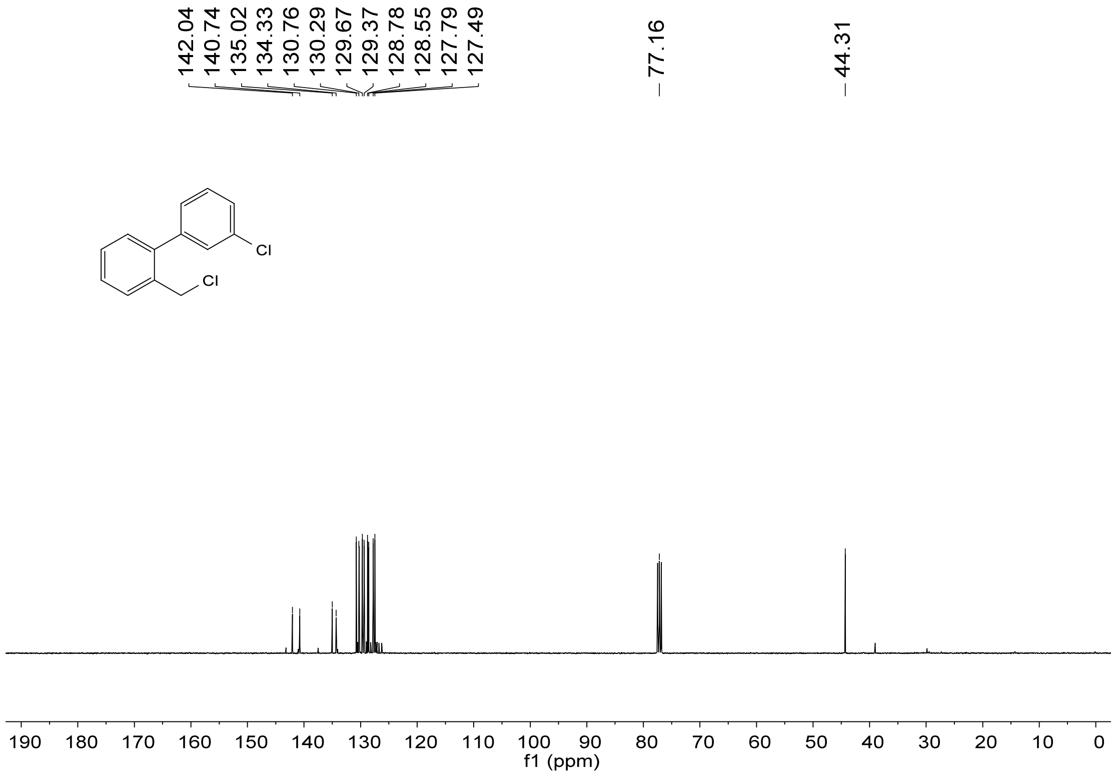

2-(chloromethyl)-2'-methyl-1,1'-biphenyl[5f]

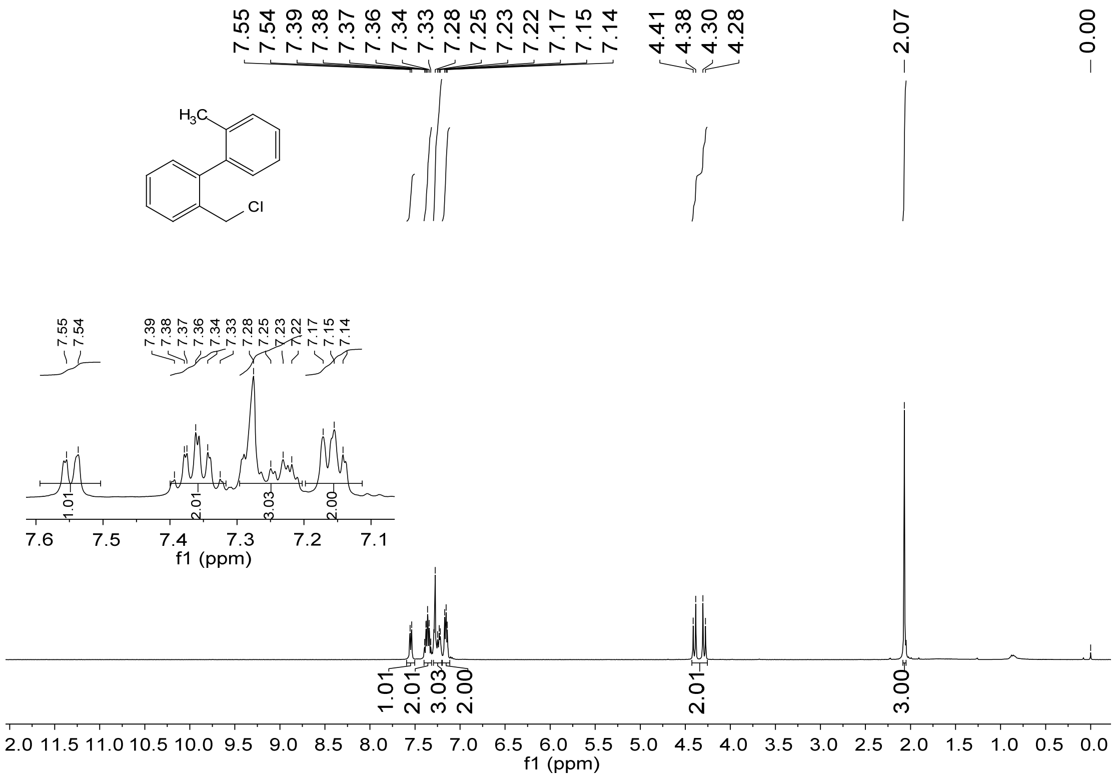

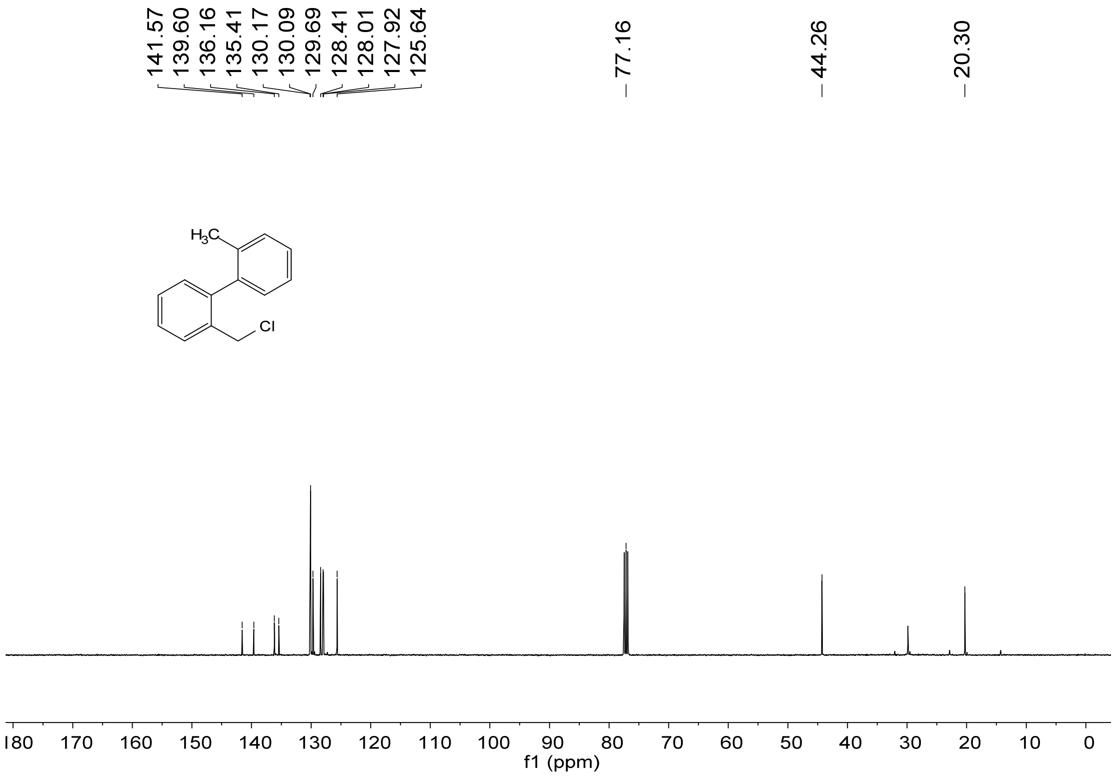

3-(2-(chloromethyl)phenyl)thiophene[5g]

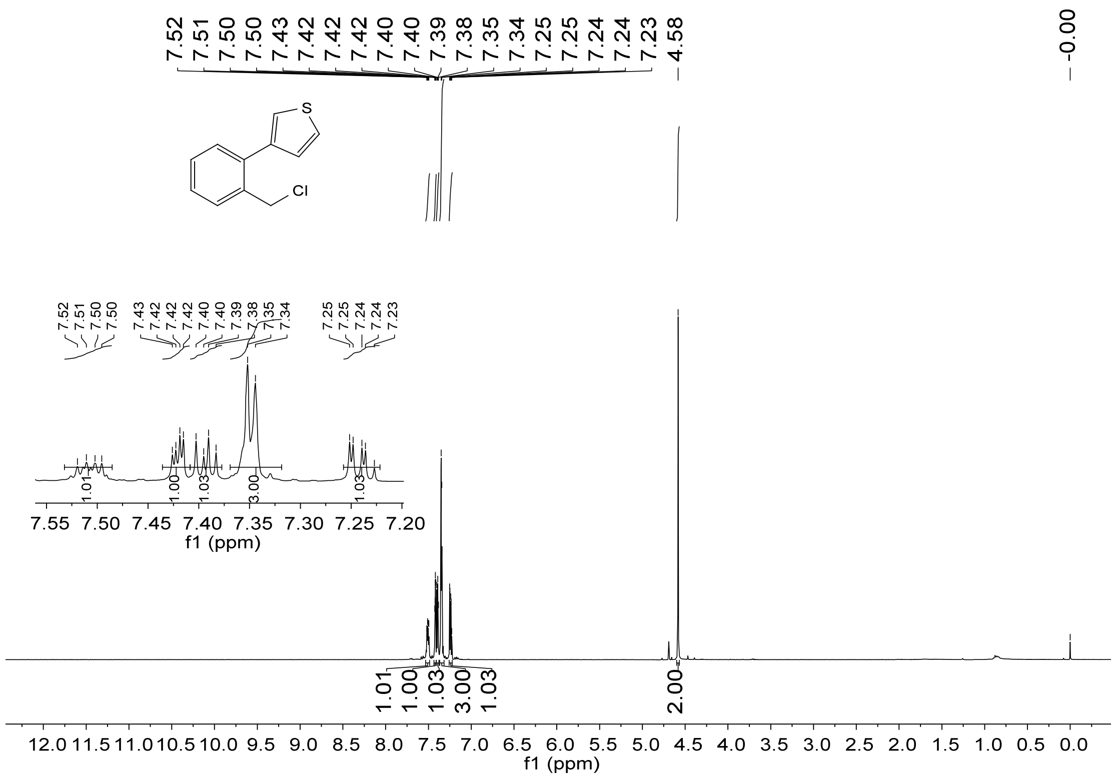

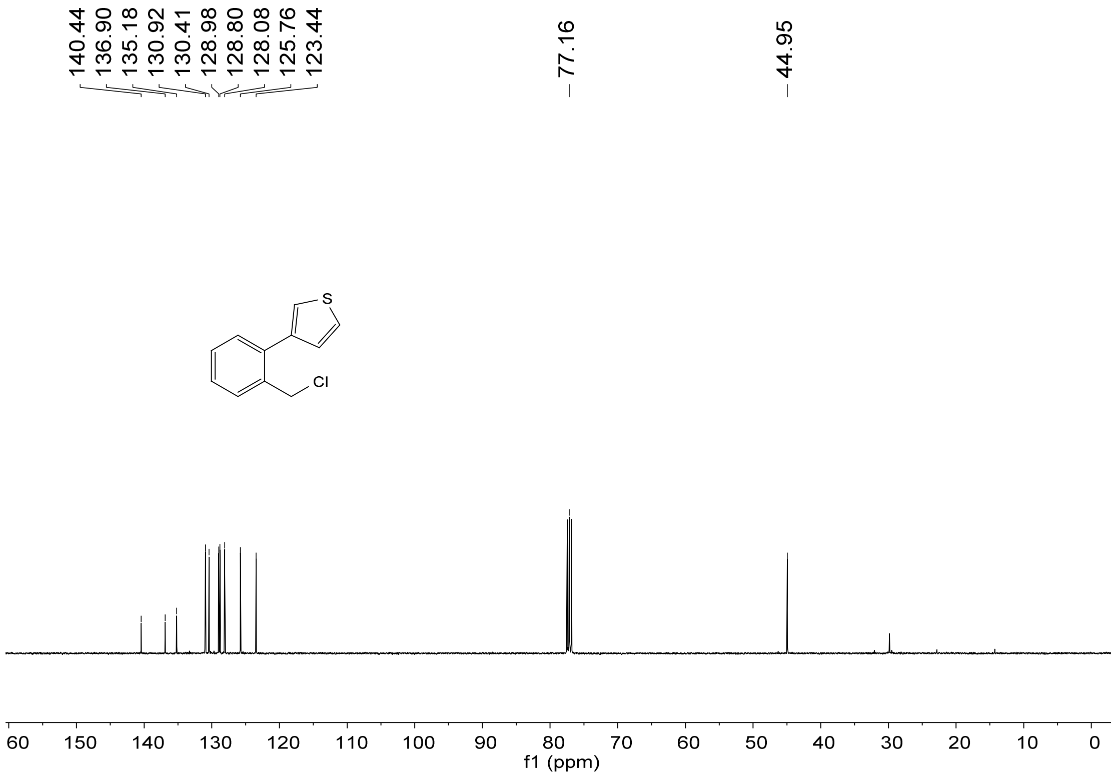

2-(2-(chloromethyl)phenyl)naphthalene[5h]

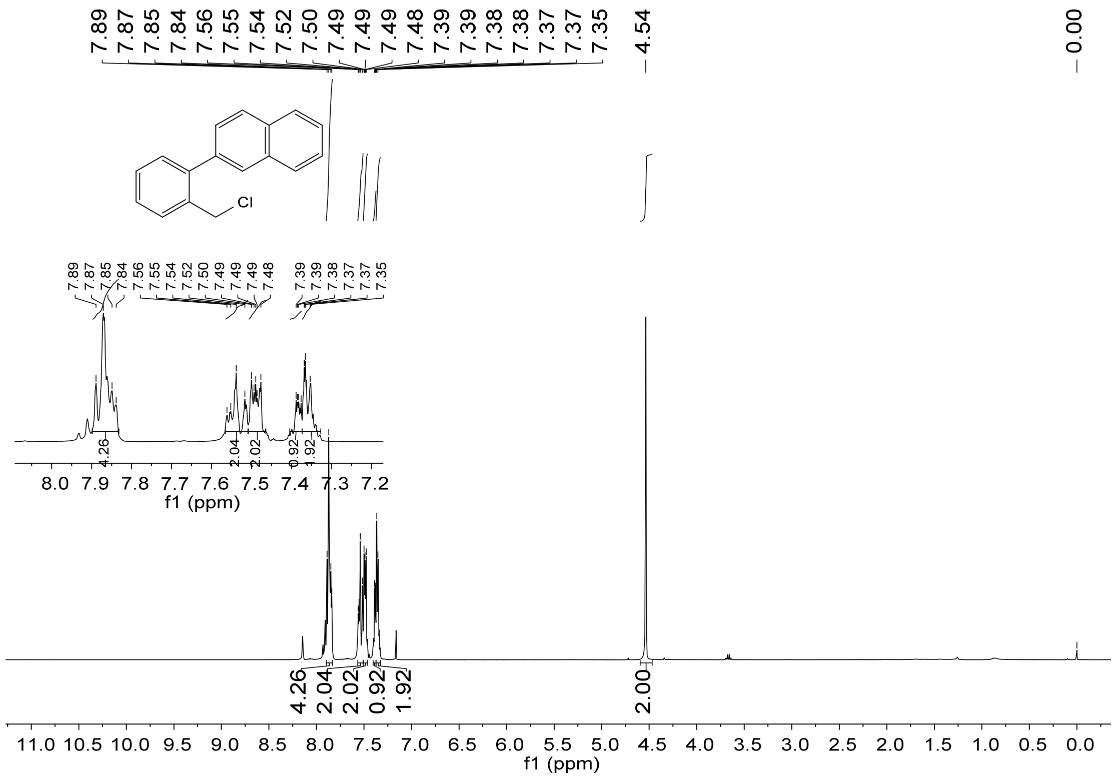

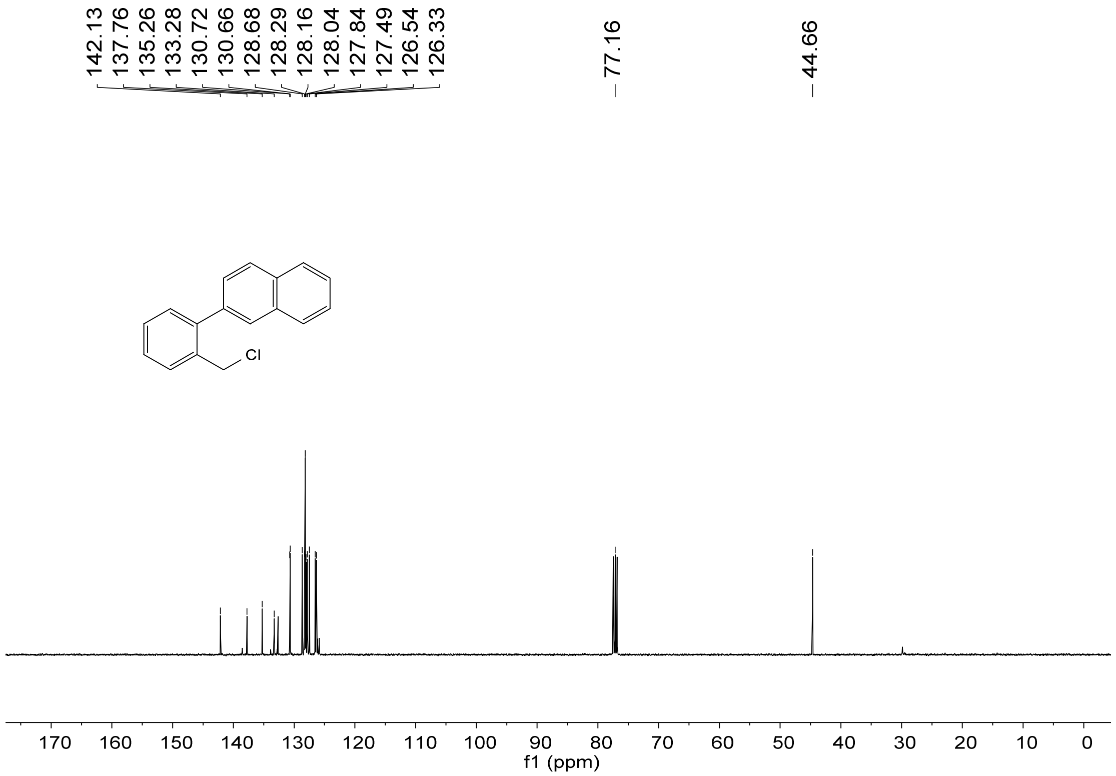

4-benzyl-4'-methyl-1,1'-biphenyl[6a]

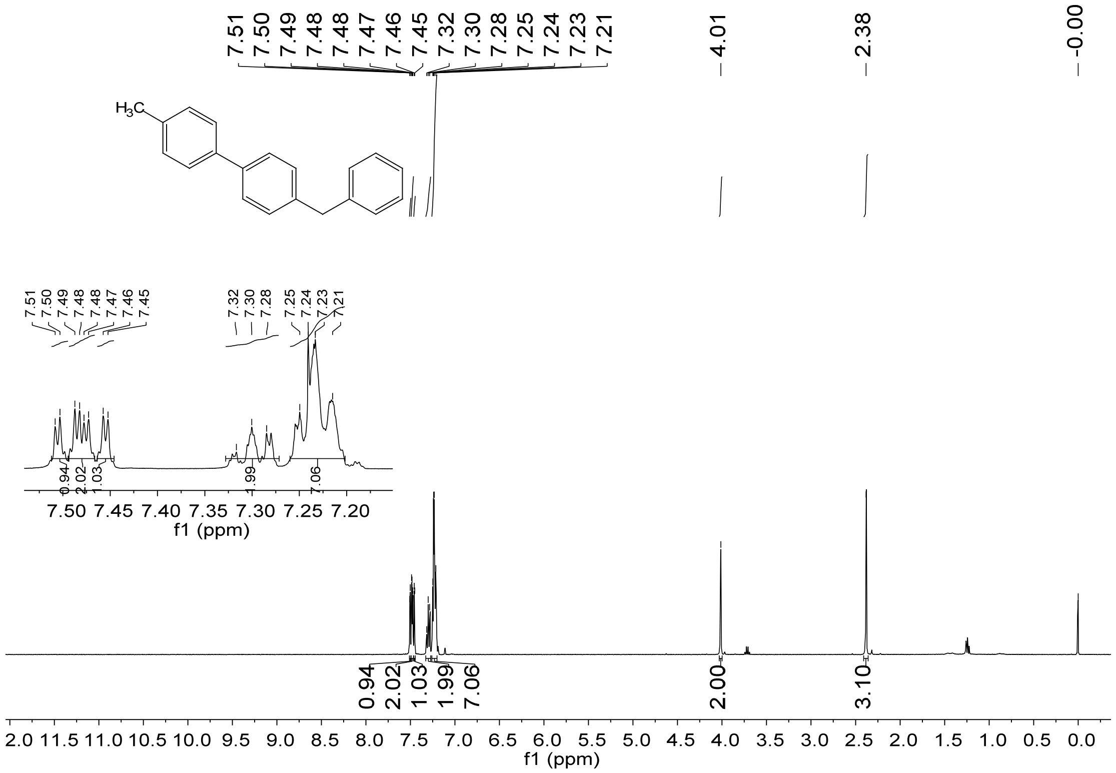

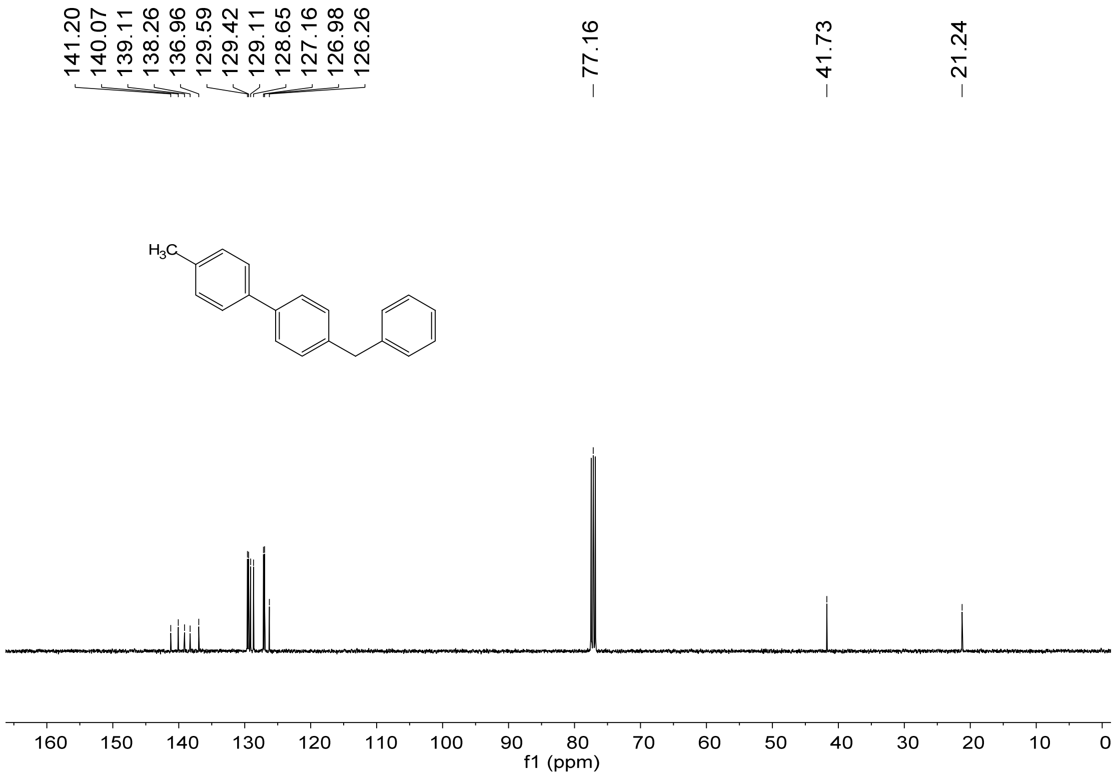

4-(4-methoxybenzyl)-4'-methyl-1,1'-biphenyl[6b]

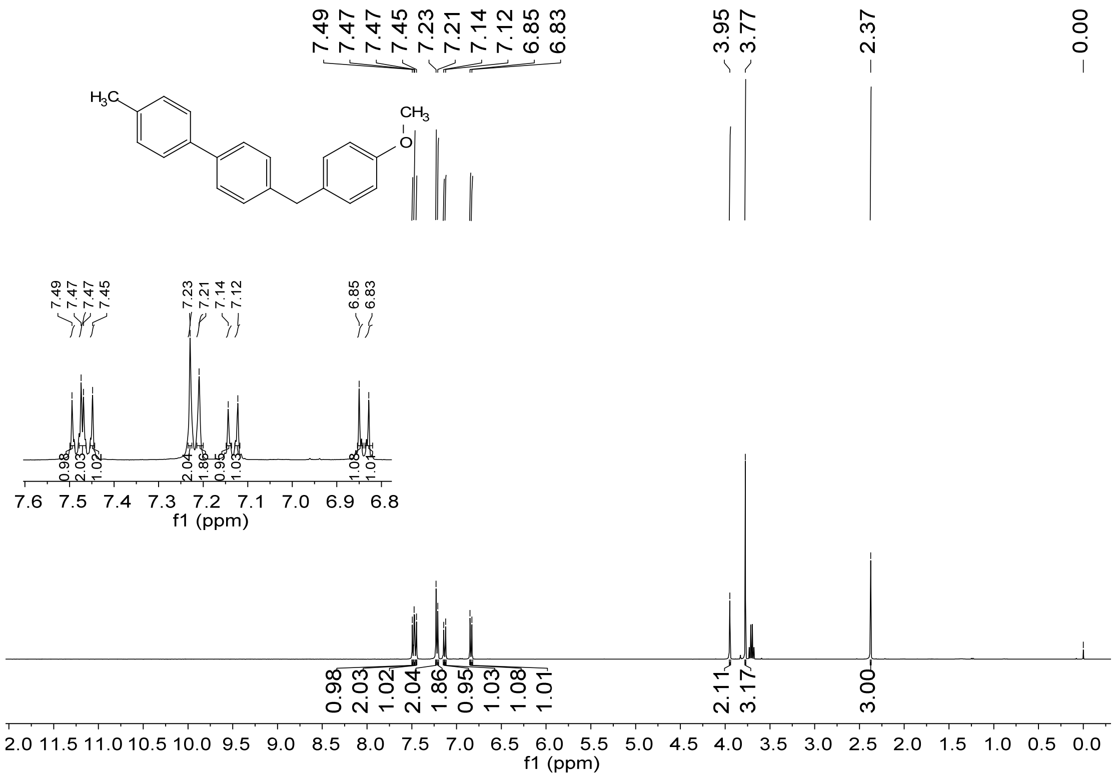

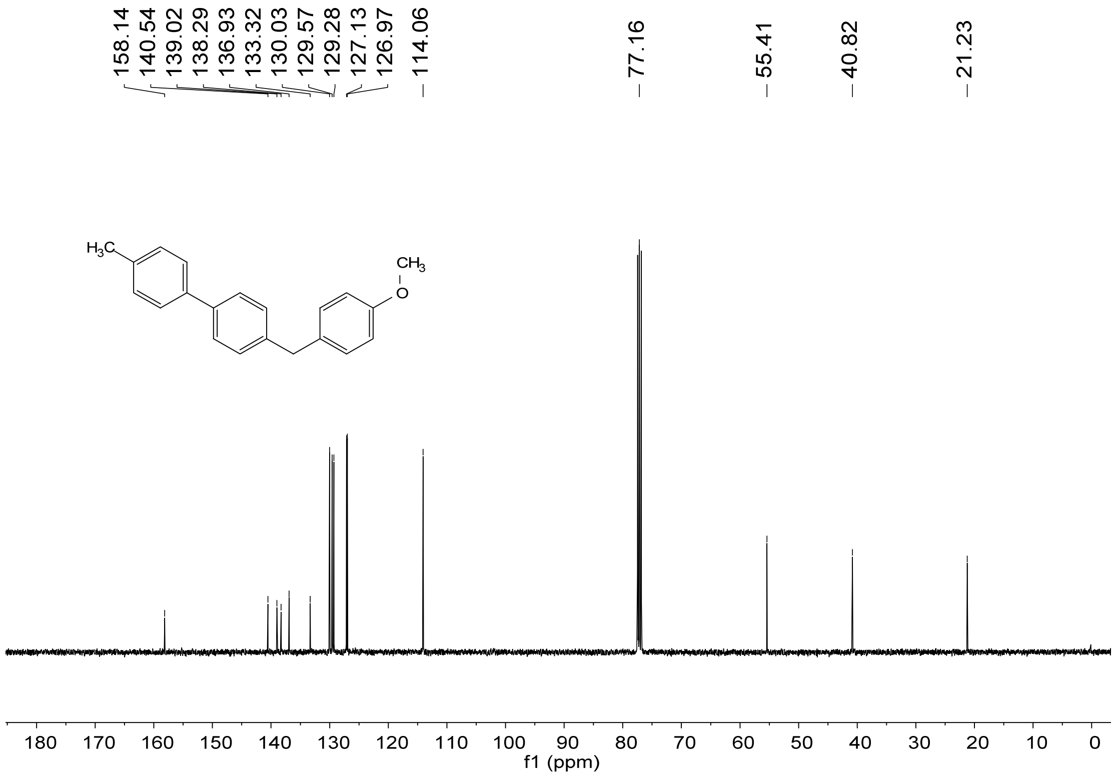

4-(4-fluorobenzyl)-4'-methyl-1,1'-biphenyl[6c]

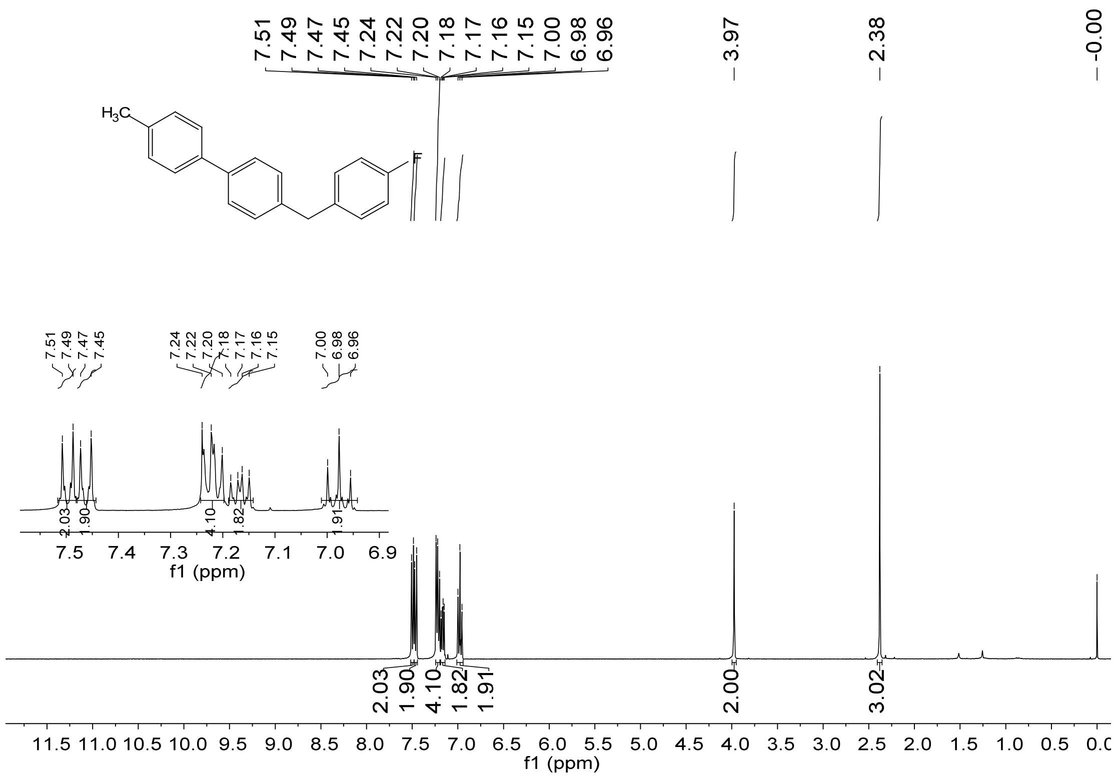

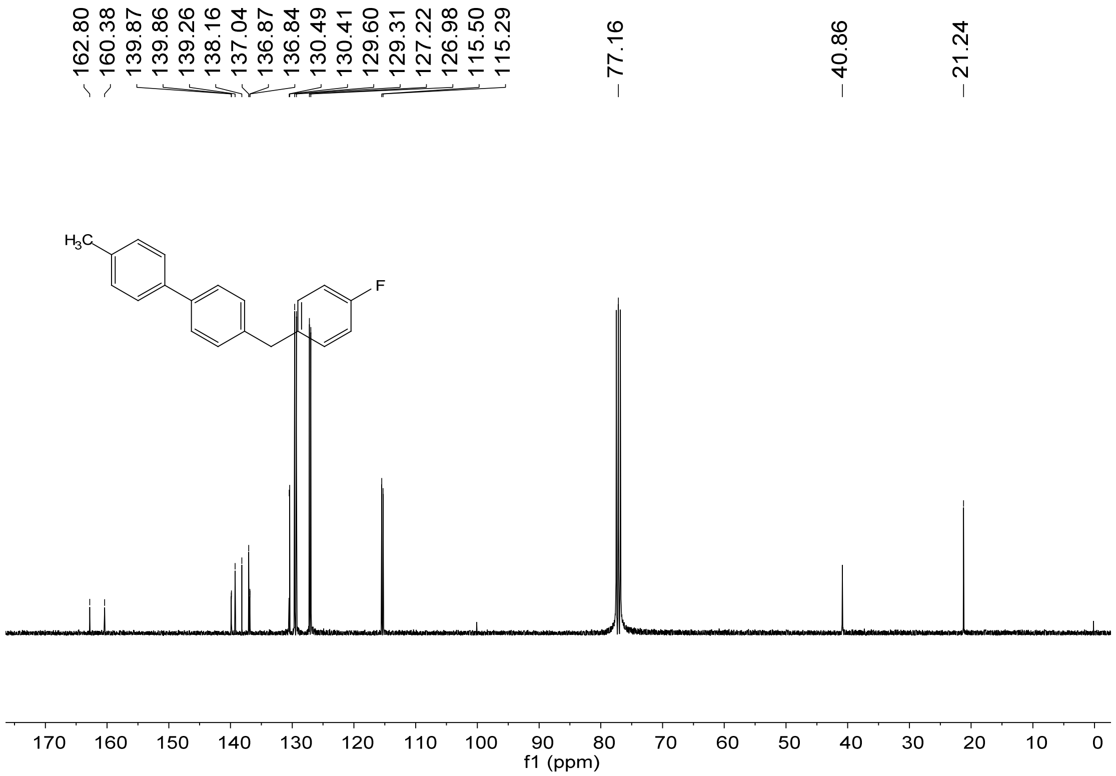

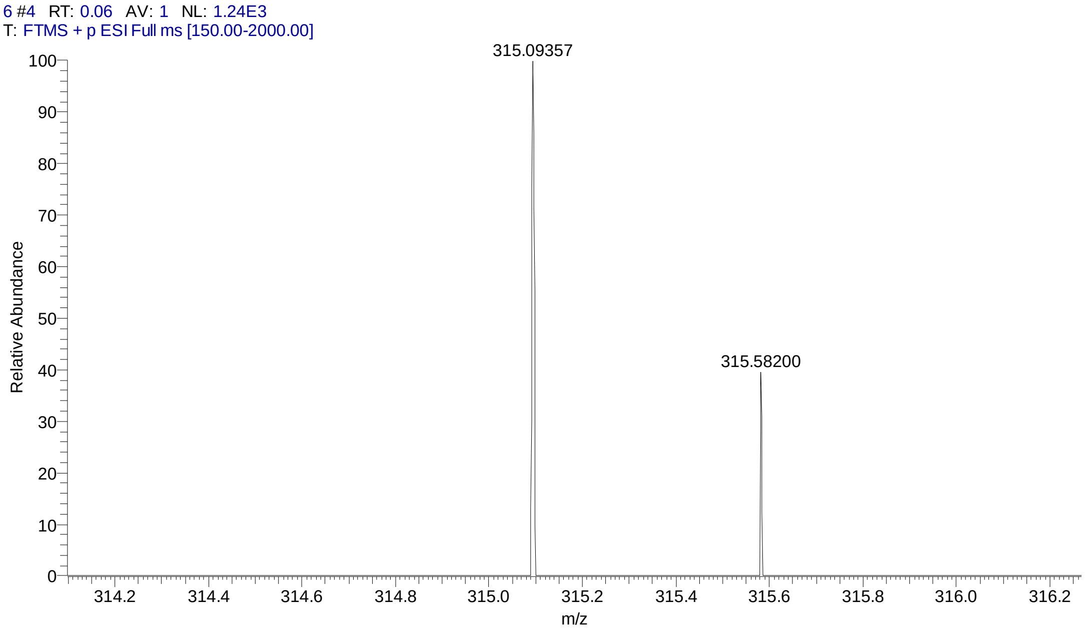

3-((4'-methyl-[1,1'-biphenyl]-4-yl)methyl)thiophene[6d]

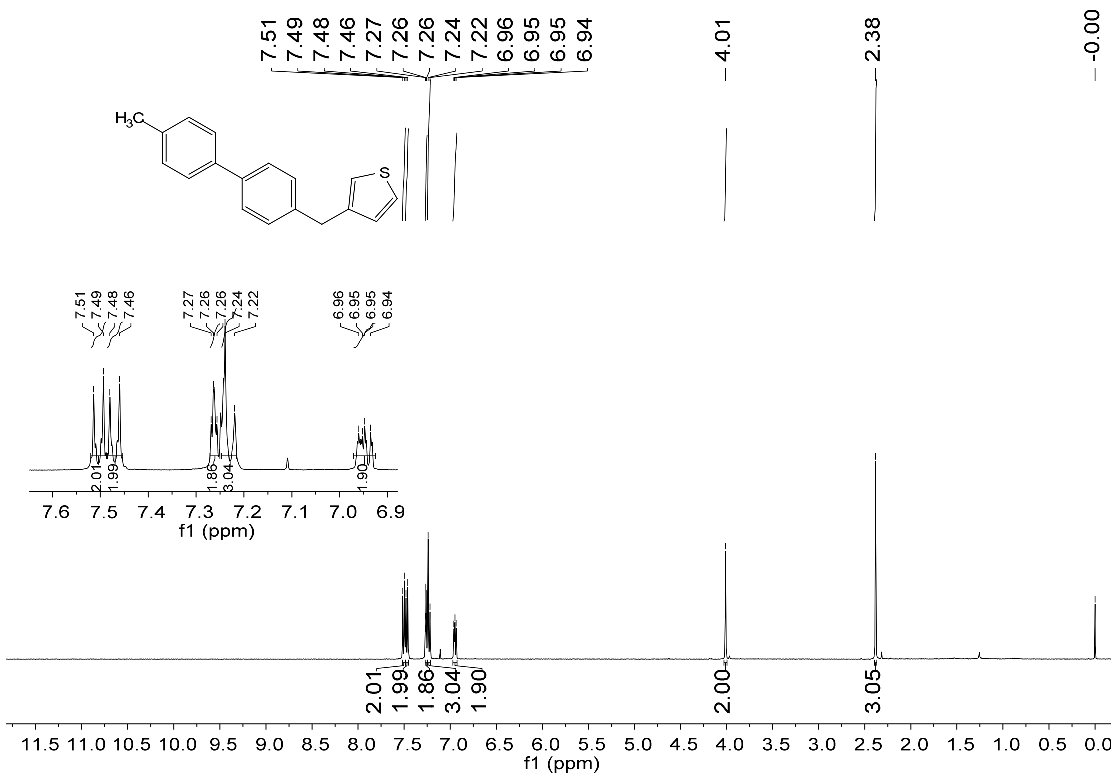
7.51
7.49
7.48
7.46
7.27
7.26
7.26
7.24
7.22
6.96
6.95
6.95
6.94
4.01
2.38
-0.00
H3C
S
2.01
1.99
1.86
3.04
1.90
2.00
3.05
f1 (ppm)

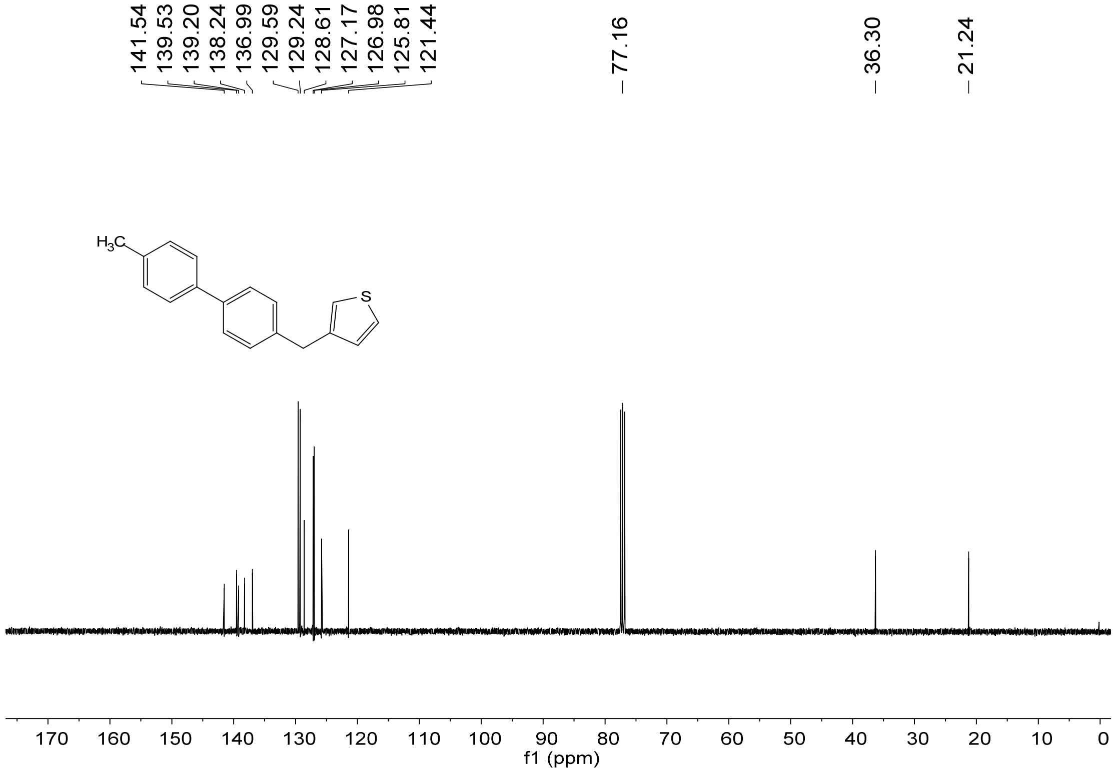
141.54
139.53
139.20
138.24
136.99
129.59
129.24
128.61
127.17
126.98
125.81
121.44
77.16
36.30
21.24
H3C
S
f1 (ppm)

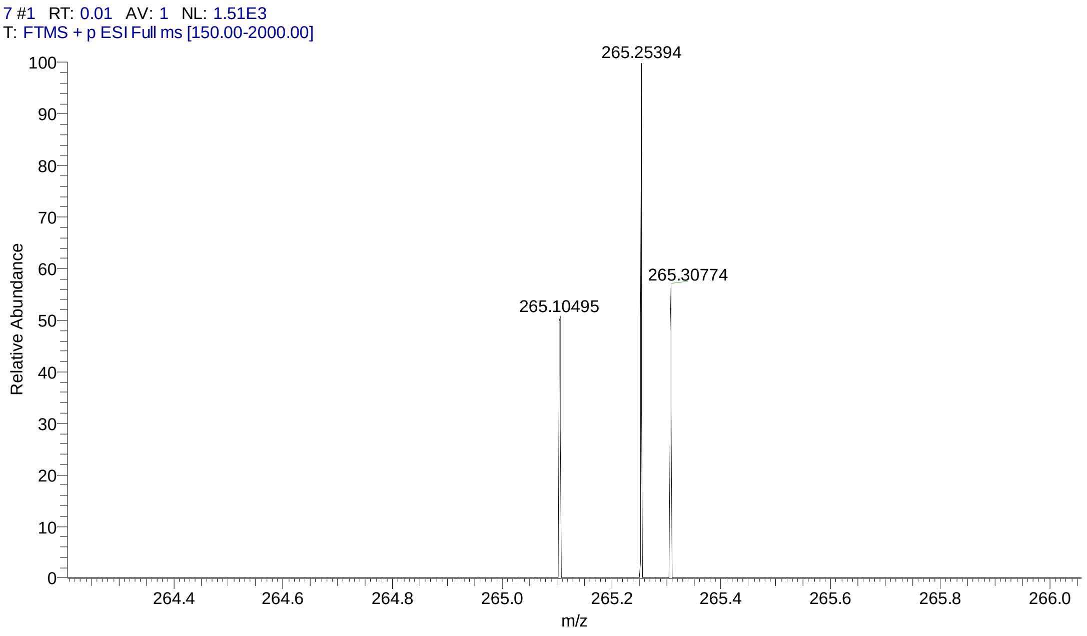

4-(bromomethyl)-4'-methyl-1,1'-biphenyl [7a]

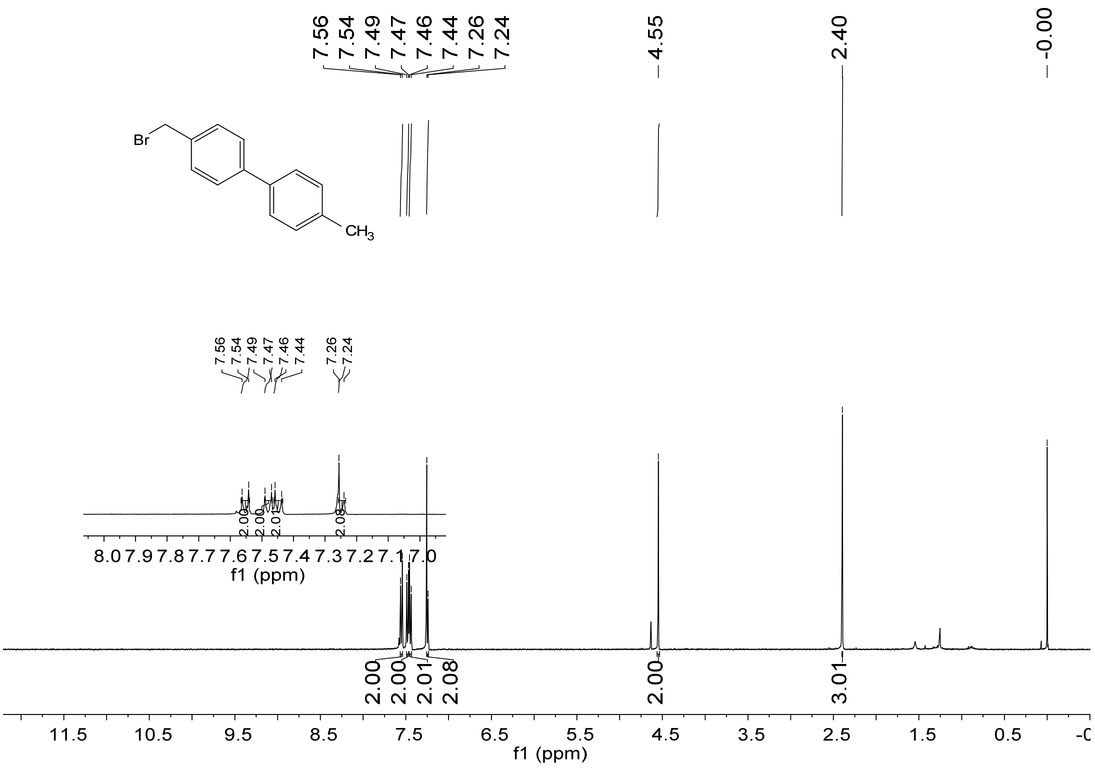

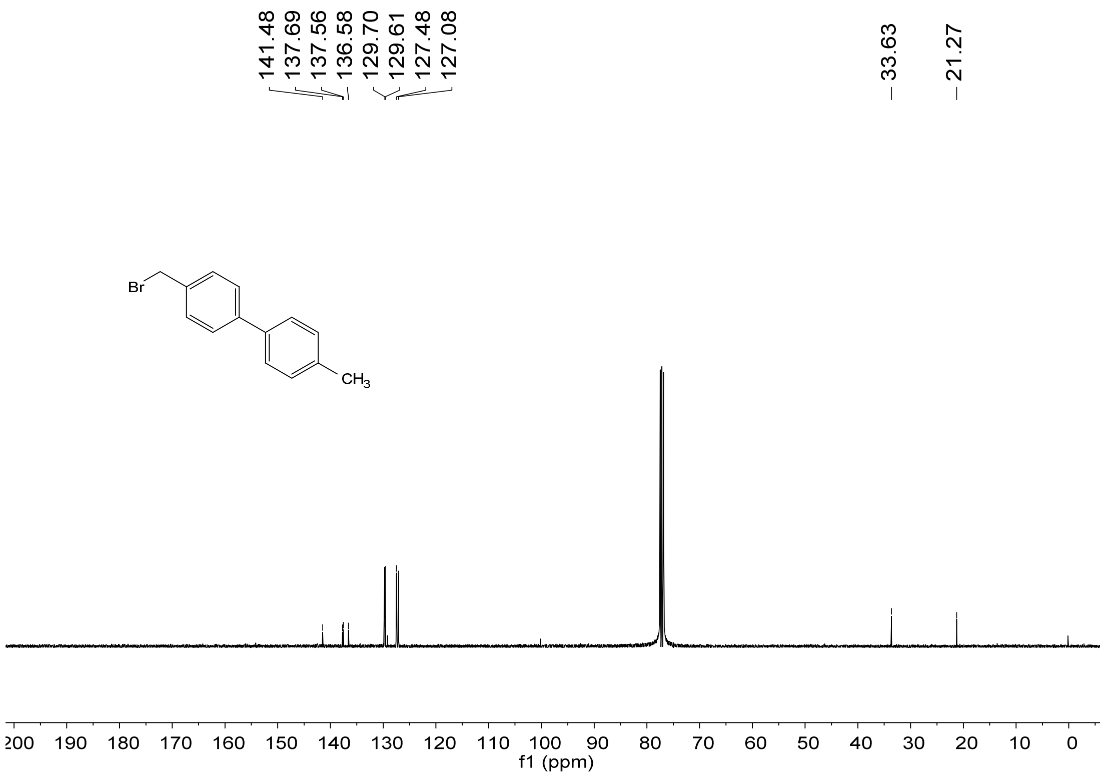

4-methyl-4'-(4-methylbenzyl)-1,1'-biphenyl [7b]

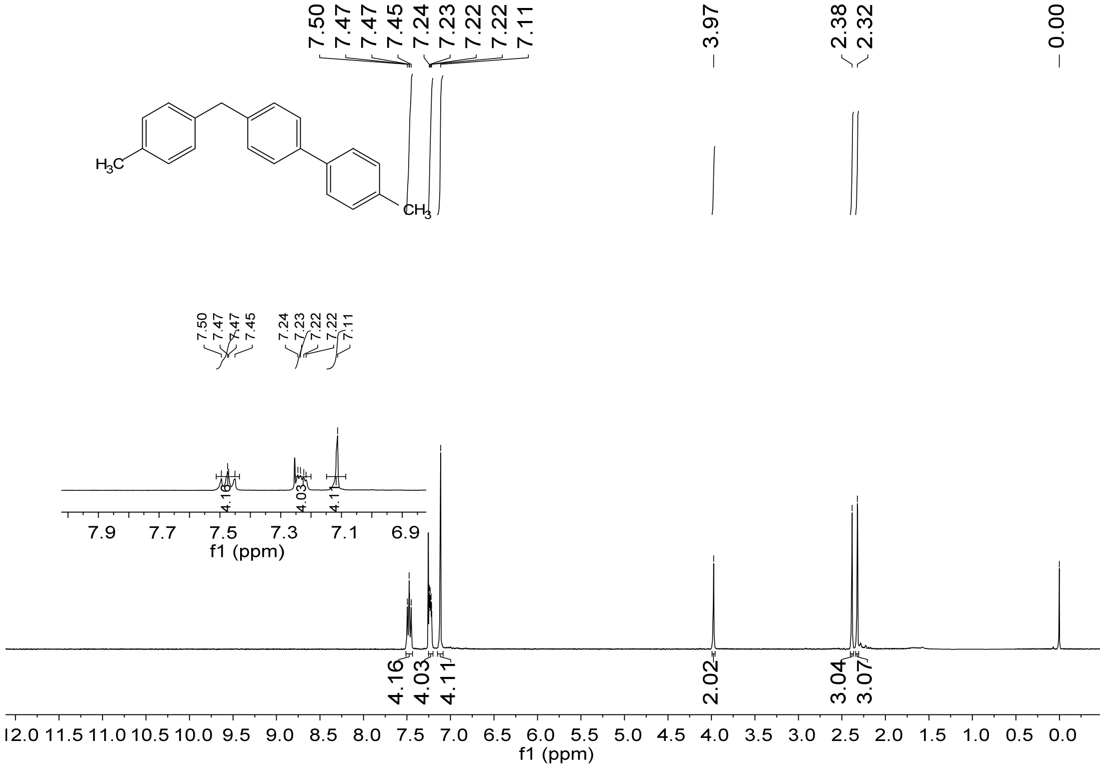

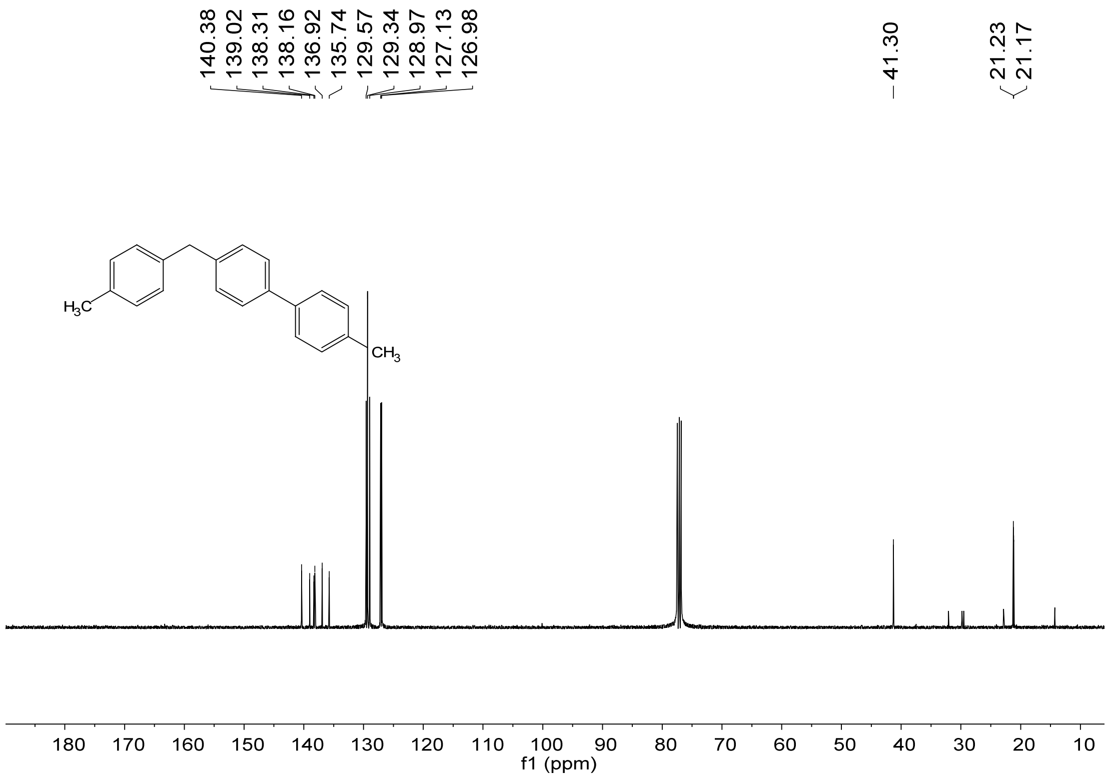

4-methyl-1,1'-biphenyl [7c]

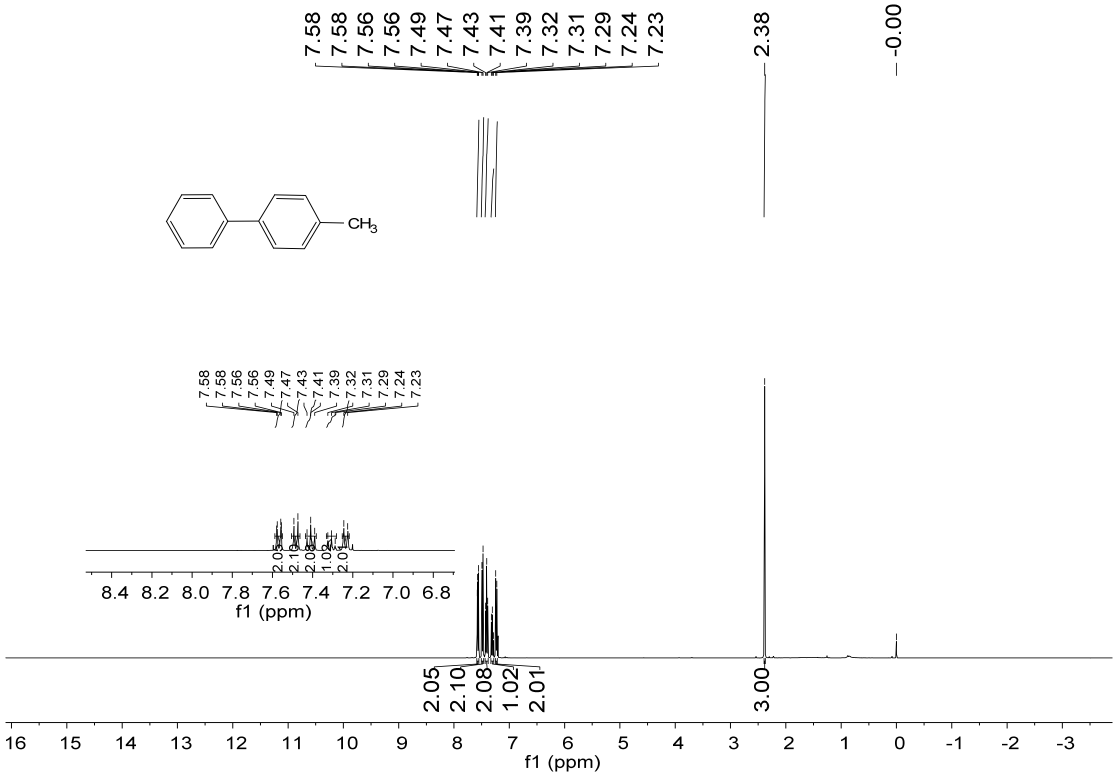

Supplement: Supplementary file 1 [file molecules-23-00433-s001.pdf]
